# eQTL discovery and their association with severe equine asthma in European Warmblood horses

**DOI:** 10.1186/s12864-018-4938-9

**Published:** 2018-08-02

**Authors:** Victor C. Mason, Robert J. Schaefer, Molly E. McCue, Tosso Leeb, Vinzenz Gerber

**Affiliations:** 1 Department of Clinical Veterinary Medicine, Swiss Institute of Equine Medicine, Vetsuisse Faculty, University of Bern, and Agroscope, Länggassstrasse 124, 3012 Bern, Switzerland; 2 Department of Veterinary Population Medicine, University of Minnesota, 1365 Gortner Ave, Saint Paul, MN 55108 USA; 3 Department of Clinical Research and Veterinary Public Health, Institute of Genetics, Vetsuisse Faculty, University of Bern, Bremgartenstrasse 109A, 3012 Bern, Switzerland

**Keywords:** *cis* eQTL, *trans* eQTL, RAO, Horses, PBMCs, Trans regulatory hotspot, GWAS

## Abstract

**Background:**

Severe equine asthma, also known as recurrent airway obstruction (RAO), is a debilitating, performance limiting, obstructive respiratory condition in horses that is phenotypically similar to human asthma. Past genome wide association studies (GWAS) have not discovered coding variants associated with RAO, leading to the hypothesis that causative variant(s) underlying the signals are likely non-coding, regulatory variant(s). Regions of the genome containing variants that influence the number of expressed RNA molecules are expression quantitative trait loci (eQTLs). Variation associated with RAO that also regulates a gene’s expression in a disease relevant tissue could help identify candidate genes that influence RAO if that gene’s expression is also associated with RAO disease status.

**Results:**

We searched for eQTLs by analyzing peripheral blood mononuclear cells (PBMCs) from two half-sib families and one unrelated cohort of 82 European Warmblood horses that were previously treated in vitro with: no stimulation (MCK), lipopolysaccharides (LPS), recombinant cyathostomin antigen (RCA), and hay-dust extract (HDE). We identified high confidence eQTLs that did not violate linear modeling assumptions and were not significant due to single outlier individuals. We identified a mean of 4347 high confidence eQTLs in four treatments of PBMCs, and discovered two *trans* regulatory hotspots regulating genes involved in related biological pathways. We corroborated previous RAO associated single nucleotide polymorphisms (SNPs), and increased the resolution of past GWAS by analyzing 1,056,195 SNPs in 361 individuals. We identified four RAO-associated SNPs that only regulate gene expression of dexamethasone-induced protein (*DEXI*), however we found no significant association between *DEXI* gene expression and presence of RAO.

**Conclusions:**

Thousands of genetic variants regulate gene expression in PBMCs of European Warmblood horses in *cis* and *trans*. Most high confidence eSNPs are significantly enriched near the transcription start sites of their target genes. Two *trans* regulatory hotspots on chromosome 11 and 13 regulate many genes involved in transmembrane cell signaling and neurological development respectively when PBMCs are treated with HDE. None of the top fifteen RAO associated SNPs strongly influence disease status through gene expression regulation.

**Electronic supplementary material:**

The online version of this article (10.1186/s12864-018-4938-9) contains supplementary material, which is available to authorized users.

## Background

Severe equine asthma (also known as recurrent airway obstruction i.e. RAO) is a chronic, potentially debilitating airway disease affecting 10–15% of horses housed in conventional management systems in temperate climates [1–3]. It has many important parallels to human asthma, including genetic effects that predispose affected horses to exaggerated mixed T helper cell (T_h_1, T_h_2, T_h_17) responses to common allergens and irritants [2–4]. Environmental irritants from dry hay or dust from various bedding materials contain antigens that elicit severe equine asthma as a delayed hypersensitivity disorder in the domestic horse *Equus ferus caballus* [3, 4]. RAO is maintained until the animal is removed from the offending environment for an extended period [5–8]. RAO is reversible, such that when sources of dust are eliminated from the environment clinical signs abate [4]. To simulate a systemic response to these antigens our group previously treated peripheral blood mononuclear cells (PBMCs) with hay dust extract (HDE), a mixture containing mold spores, mites, inorganic dust particles, and plant fragments [8–10]. Differential expression and genome wide association (GWAS) studies that included samples used in this study, have identified differentially expressed genes and genomic regions suggestively associated (*p* ≤ 1e-5) with RAO [9, 11–13]. However, no RAO associated coding variants have been found that explain the GWAS signals, so it was hypothesized that the causative variant(s) underlying these signals were likely non-coding regulatory variant(s) [12]. Non-coding variants often have poorly defined or unknown functions, especially in non-model organisms. However, regulatory function can be inferred for genetic variants associated with gene expression levels.

Regions of the genome containing variants that influence the number of expressed RNA molecules are expression quantitative trait loci (eQTLs) [14]. eQTLs have been central to our understanding of global transcriptional regulation by genotype [14]. eQTL studies can be powerful extensions to GWAS by identifying disease associated single nucleotide polymorphisms (SNPs) that overlap, or are in linkage disequilibrium with, eQTL SNPs (eSNPs) that regulate gene expression of a gene in disease relevant tissues [15–19]. An eSNP that is also a disease-associated SNP can influence the disease phenotype through gene expression regulation, or the SNP can have pleiotropic effects that independently influence the disease phenotype and gene expression regulation [20, 21]. Therefore, additional testing for association between gene expression and disease status is required to determine if gene expression regulation could contribute to the disease phenotype. A mediation analysis can identify the genetic effect on disease risk specifically mediated through gene expression [22]. These analyses could be especially important for identifying candidate genes regulated by SNPs that lie outside of genic or functionally annotated genomic regions. However, eQTLs remain undiscovered for many species with previously identified disease associated SNPs.

A typical eQTL analysis predicts additive changes in gene expression by genotype. eQTLs are generally described in two contexts, local eQTLs and distant eQTLs. Local eQTLs are genomic regions containing a variant that is associated with expression of a gene ‘close’ to the variant (within a specified distance). Local eQTLs can be caused through two mechanisms: 1) local *cis* eQTLs which affect gene expression on the same strand as the variant and cause allele specific expression (ASE), or 2) local *trans* acting eQTLs that affect gene expression indirectly by regulating diffusible regulatory elements, such as microRNA (miRNA) or transcription factors (TFs), which subsequently influence gene expression of alleles on both homologous chromosomes equally [14]. The regulatory variants responsible for local *cis* eQTLs are most often near the transcriptional start site (TSS) [15, 16]. Distant eQTLs are genomic regions containing a variant that are associated with expression of a gene outside of the specified local window far away on the same chromosome or on a different chromosome. Distant variants that regulate gene expression often act in *trans* by structurally altering the diffusible regulatory molecules, or by altering the diffusible element’s gene expression in *cis* that then affect the target gene’s expression in *trans* [15]. Here, we do not search for ASE and therefore can only describe eQTLs as local or distant. However, we assume that the majority of local and distant eQTLs are *cis* acting and *trans* acting eQTLs respectively, and therefore describe all eQTLs as *cis* or *trans*. Single variants have been identified that regulate many different genes in *trans*. These *trans*-regulatory hotspots can regulate many genes significantly enriched for similar biological pathways such as maintaining cell homeostasis [23]. This indicates that *trans* regulatory hotspots might co-regulate many genes by a common mechanism [23, 24].

eQTLs can be cell type or treatment specific [17]. To represent gene expression regulation under multiple treatments, we analyzed peripheral blood mononuclear cells (PBMCs) from European Warmblood horses in vitro under four different conditions: no treatment (MCK) to represent baseline RNA expression, lipopolysaccharides (LPS) to mimic an inflammatory response, recombinant cyathostomin antigen (RCA) to mimic response to parasitic antigens, and hay-dust extract (HDE) to mimic RAO exacerbation in susceptible horses [8, 9]. We performed *cis* and *trans* eQTL analyses on PBMCs, and GWAS on imputed genotypes in healthy and RAO affected European Warmblood horses to investigate: 1) how *cis* and trans eQTLs are associated with gene expression, 2) if *trans* regulatory hotspots regulate genes in related biological pathways, 3) if suggestively significant RAO associated SNPs regulate expression of biologically relevant candidate genes, and 4) if RNA expression predicts disease status for genes regulated by disease associated eSNPs. To the best of our knowledge, this is the first genome wide eQTL study in horses. Our results improve understanding of transcriptional regulation in horses, and utilize GWAS and eQTL analyses to attempt to identify candidate genes that contribute to RAO through gene expression regulation.

## Results

We performed eQTL analyses on PBMCs under four conditions: MCK, LPS, RCA, and HDE with *Matrix eQTL* utilizing tag SNPs and aligned RNAseq data for 42 RAO-affected and 40 control European Warmblood horses [8, 9, 25]. Tag SNPs are a selected subset of SNPs chosen to represent the variation in haplotypes and increase power of eQTL detection by reducing the burden of multiple testing (Methods). However, we also used a filtered subset of imputed SNPs (1,056,195) to increase resolution in GWAS and eQTL analyses when we searched for eSNPs that are also RAO associated SNPs. We filtered data to remove eQTLs driven by outliers, and searched for differentially expressed genes regulated by eSNPs that were also RAO associated SNPs (Methods).

### Gene and individual filtration

**Fig. 1:**
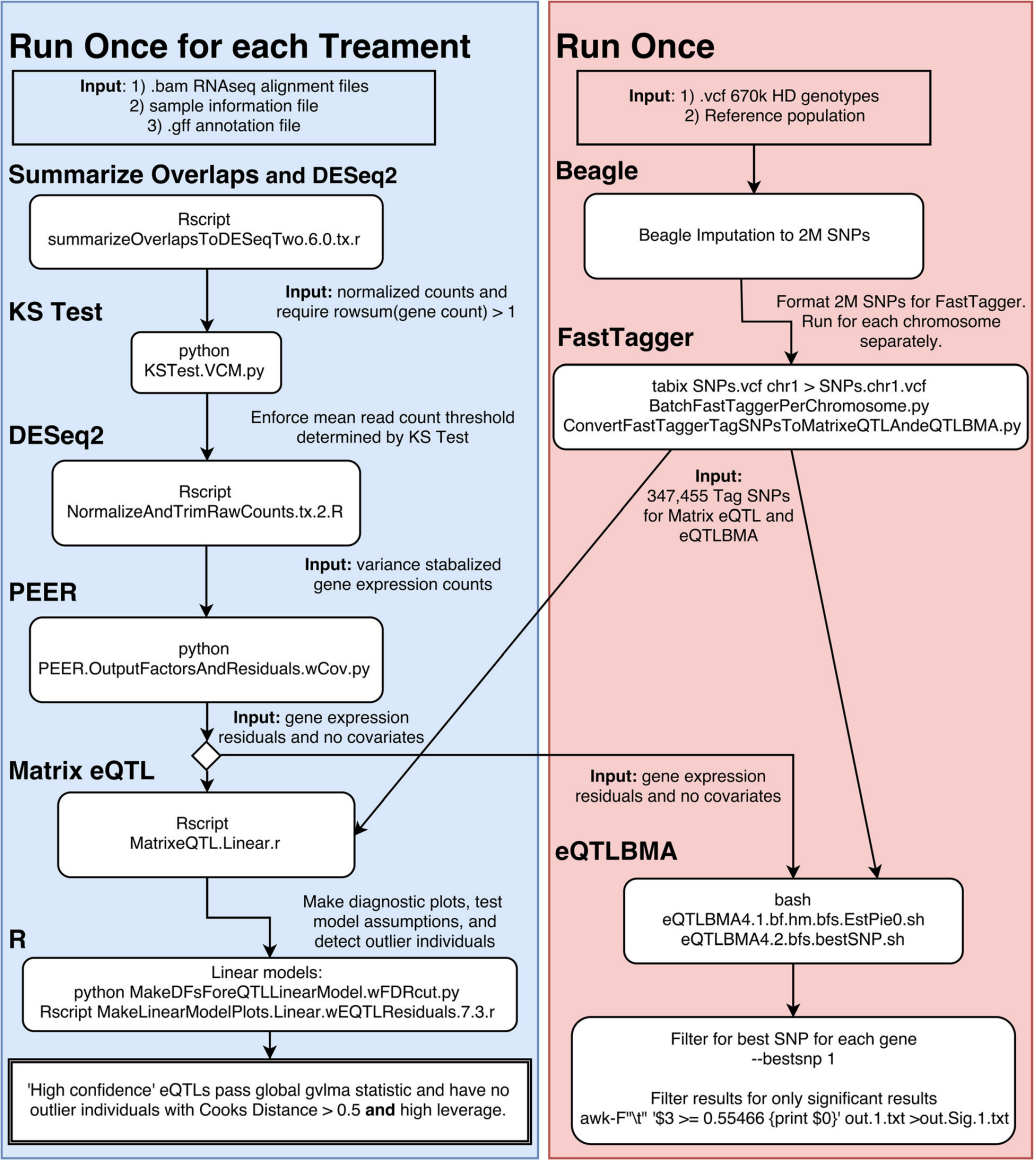
Methods flow chart. Describes the sequence of analyses and programs used for tag SNP eQTL analyses in this study. Code available: https://github.com/VCMason

Prior to eQTL analysis, we removed genes with mean read counts below a read count threshold determined using the Kolmogorov-Smirnov (KS) test (Table 1) (Fig. 1, Additional file 1: Figure S1) [26, 27]. This reduced the number of NCBI annotated genes analyzed to a mean value of 12,736 genes across the four treatments of PBMCs, and RNAseq counts were normalized and variance stabilized for each treatment (Additional file 1: Figure S2). One sample was removed prior to all analyses based upon a principle component analysis (PCA) of genotypes where it did not cluster with our defined family groups (Additional file 1: Figure S3). No additional individuals were removed based on PCAs of genotypes, PCAs of RNA expression, or additional filtering criterion (Additional file 1: Figures S2 and S3) (Methods).

**Table 1:** KS Test trimmed and normalized read count cutoffs and numbers of genes after each filter

Treatment	MCK	LPS	RCA	HDE	Mean
All NCBI Genes	26,707	26,707	26,707	26,707	26,707
Number of genes with > 1 read mapped	23,804	23,855	23,869	23,899	23,857
Number of genes after mean cutoff	13,058	12,520	12,792	12,574	12,736
Number of genes after mean cutoff and common to all four treatments				12,254	
KS-Test: Mean Read Count Cutoff	20	23	22	24	22.25
KS-Test: Median Read Count Cutoff	18	20	20	24	20.5

Four in vitro treatments of PBMCs from European Warmblood horses: no treatment (MCK), lipopolysaccharide (LPS), recombinant cyathostomin antigen (RCA), and hay dust extract (HDE) [9]

### Identification of tag SNPs

SNPs were genotyped on the MNEc670 array (*n* = 636,897) then imputed to a higher density (*n* = 1,926,709) using a reference population of 485 horses [28–30]. We calculated 98.6% concordance between imputed variants and genome wide sequencing variants for the two sires of the family groups (Fam1 & Fam2) (Methods). From the imputed and phased SNP dataset we calculated 347,455 tag SNPs for the 82 individuals used in eQTL analyses [31].

### Matrix eQTL: Single treatment analyses

**Fig. 2:**
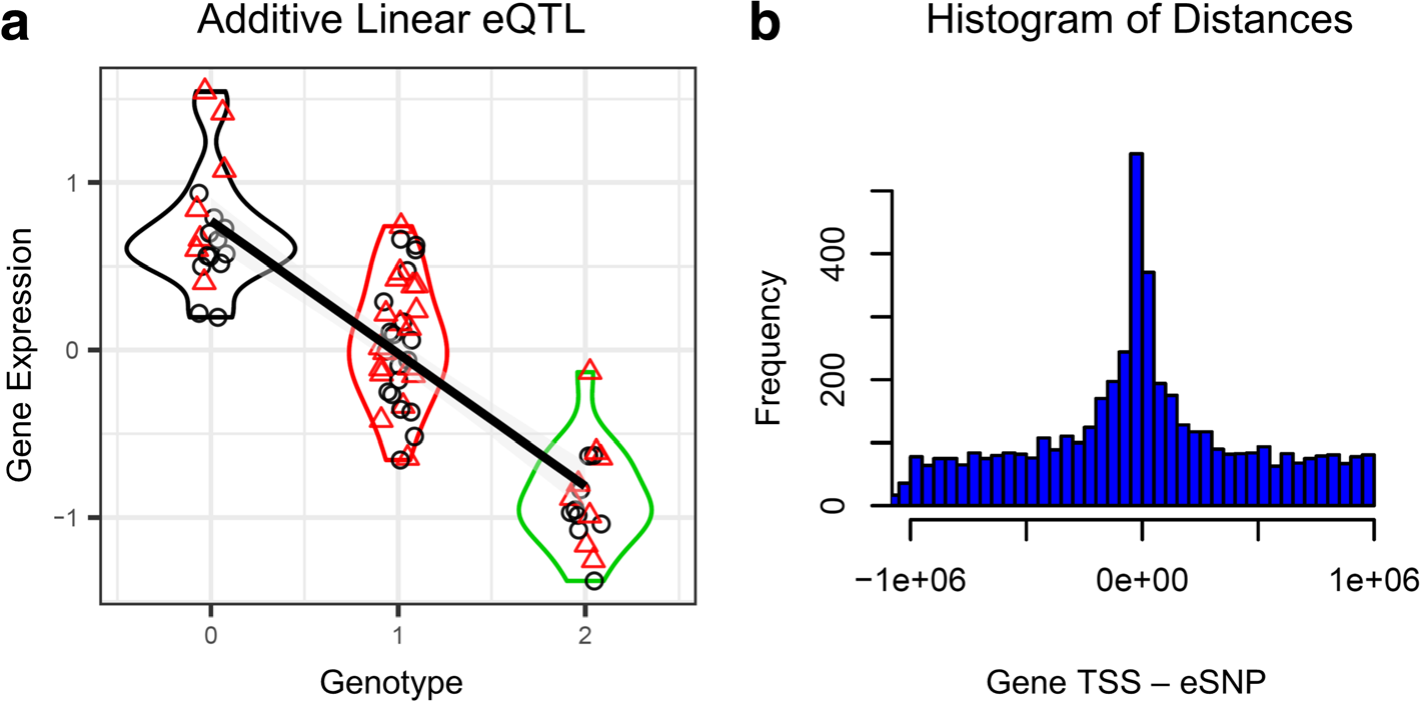
eQTLs. **a** Linear regression for the effect of genotype (homozygous reference = 0, heterozygous = 1, and homozygous alternative = 2) on gene expression for gene glucosidase alpha (*GAA*) in treatment MCK1. The line was fitted to all individuals and grey shading is the standard error. Red triangles are cases, and black circles are controls. Density functions surround plot points: black for genotype 0, red for genotype 1, and green for genotype 2. Here, one unit change in genotype is a good predictor for an additive change in gene expression. This eQTL implies that some variant inside the QTL (surrounding the significant SNP) is regulating gene expression. **b** Histogram showing the frequency of distances between a genes’ transcription start site (TSS) and the eSNP that is associated with that genes’ expression in the HDE treatment

Additive linear *cis*- and *trans*- eQTLs for tag SNPs with the lowest false discovery rate (FDR) value for each gene, and for each treatment (MCK, LPS, RCA, and HDE) are summarized in Table 2 and listed in Additional file 1: Tables S1-S16 (Fig. 2a, Additional file 1: Figure S4) (Methods). We detected a mean of 5535 statistically significant (FDR < 0.05) linear *cis* eQTLs (one eSNP per gene) across all four treatments with *Matrix eQTL* (Table 2). We determined, a mean of 1219 *cis* eQTLs (one eSNP per gene) across all four treatments to be unreliable and therefore low confidence eQTLs, while the remaining 4316 *cis* eQTLs were classified as high confidence (Table 2, Additional file 1: Figure S5) (Methods). On average across all four treatments, we detected high confidence *cis* linear eQTLs in 33.8% of the 12,736 genes analyzed with the linear model in *Matrix eQTL* (Table 1, Table 2). *Cis* eSNPs were significantly enriched near gene transcription start sites (*p* < 2.2e-16) (Fig. 2b). We found a significant difference (*p* < 1.0e-6) in the proportion of eSNPs in genic (5’-UTR, exons, introns, 3’-UTR) regions between *cis* (0.346) and *trans* (0.483) eSNPs (Additional file 1: Table S17).

**Table 2:** Number of significant eQTLs identified and sorted according to confidence level in all treatments and all models

Treatment:	MCK	LPS	RCA	HDE	Mean
	Linear Model, *Cis* eQTLs, FDR < 0.05				
Matrix eQTL: Number of eQTLs, All	5045	5750	5218	6127	5535
Matrix eQTL: Number of eQTLs, Low Confidence	1207	1250	1153	1266	1219
Matrix eQTL: Number of eQTLs, High Confidence	3838	4500	4065	4861	4316
	Linear Model, *Trans* eQTLs, FDR < 0.05				
Matrix eQTL: Number of eQTLs, All	1244	1463	1088	3496	1823
Matrix eQTL: Number of eQTLs, Low Confidence	397	379	294	800	468
Matrix eQTL: Number of eQTLs, High Confidence	847	1084	794	2696	1244

### eQTLs shared across treatments

**Fig. 3:**
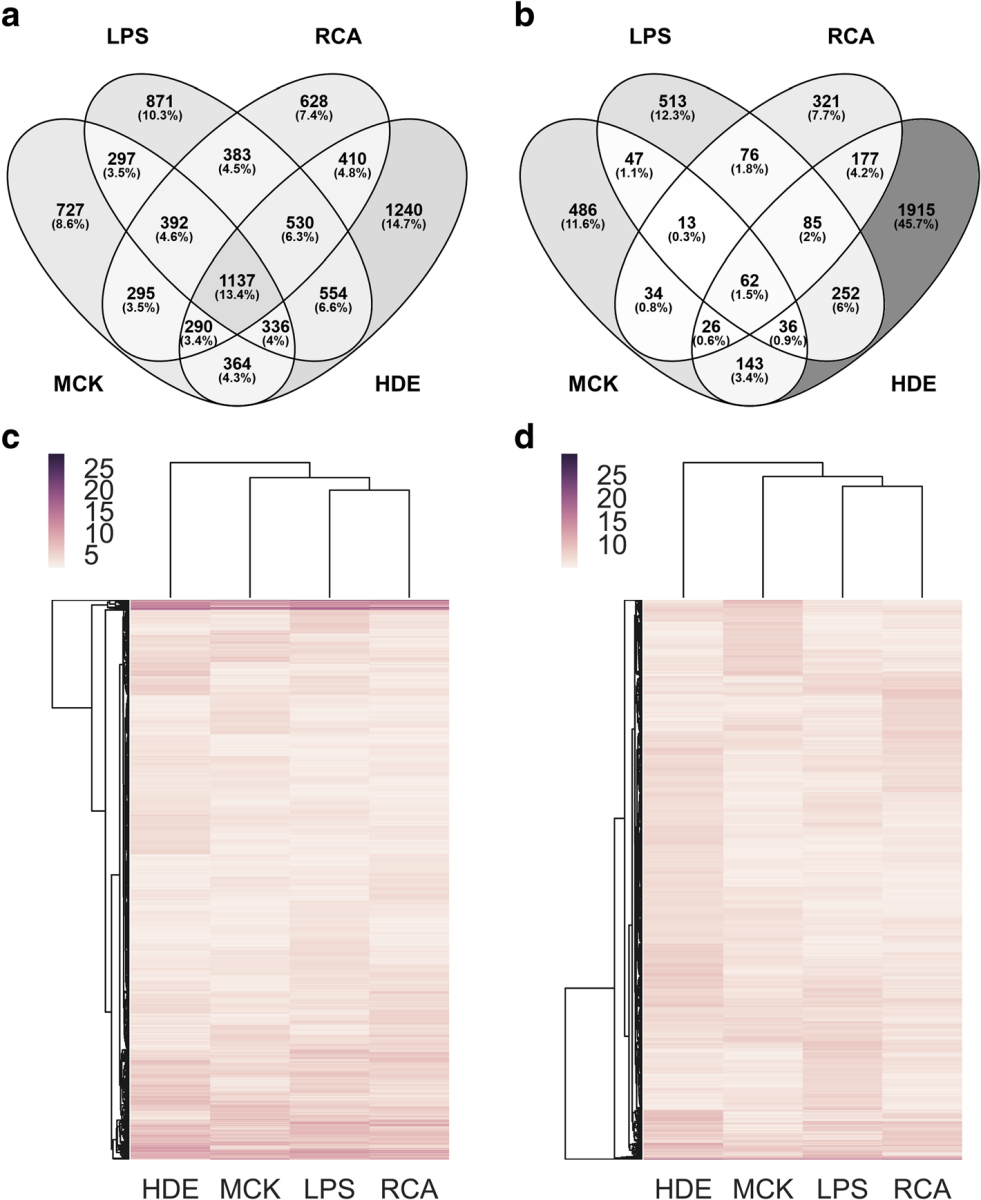
Number of genes associated with a *cis*-eSNP shared across all four treatments and comparison of eQTL *p*-values between the four treaments. eQTLs were calculated with Matrix eQTL. Venn diagram shows the number of genes shared and unique to all treatments of PBMCs for **a**) *cis* and **b**) *trans* eQTLs identified from Matrix eQTL analyses. *P*-values were compared across all four treatments for eQTLs that were significant in at least one treatment and all eQTLs must have had a raw p-value <1e-2 in **c**) *cis* and **d**) *trans*. Each p-value was transformed with –log10(pvalue) and hierarchically clustered. We compared 4066 eQTLs in *cis* 4582 eQTLs in *trans*

Separate eQTL analyses of treatments in *Matrix eQTL* indicate that the majority of genes regulated by eSNPs in *cis* are specific to one treatment (41.0%) or shared between two treatments (27.7%), while only 18.3% were shared across three treatments and 13.4% of all genes were shared across four treatments (Fig. 3a). To visualize how significance of eQTLs changed between treatments, we compared *p*-values for high confidence *cis* and *trans* eQTLs that were significant in at least one treatment in a heatmap with hierarchical clustering (Fig. 3c–d). We also jointly modeled the four treatments with eQTL by Bayesian Model Averaging (*eQTLBMA*), which identified 3990 significant eQTLs (one eSNP per gene) almost all of which were shared across all treatments (Additional file 1: Figure S6. Additional file 1: Table S18, and S1 Text). More *cis* eSNPs and genes were shared in different treatments in the *eQTLBMA* analysis than in the *Matrix eQTL* analysis (Fig. 3a, Additional file 1: Figure S6A). However, 94.2% of the same genes were significantly associated with an eSNP in both analyses. With *Matrix eQTL*, the large discrepancy between the number of genes shared across all treatments regulated by any eSNP (1137) (one eSNP per gene with lowest FDR) and the number of genes regulated by the same eSNP (140) indicated that the majority of high confidence eSNPs with lowest FDR differ across treatments. However, this result could be influenced by stochastic noise, or ‘best’ eSNP selection procedures.

To test if the genes shared across all treatments regulated by different eSNPs identified by *Matrix eQTL* are regulated by the same genomic regions, we constructed a distribution of the distances between SNP chromosomal coordinates for all six pairwise combinations of treatments for the 997 genes shared across all treatments that have different eSNPs (Additional file 1: Figure S7). The distribution is heavily skewed towards zero indicating that eSNPs with lowest FDR values for each treatment, although different, often are not distant from eSNPs with lowest FDR values identified in another treatment. We constructed a null distribution of distances between all SNPs within 100 random 2 Mb windows on chromosome 1. We compared the two distributions of distances and found them to be significantly different (*p* < 2.2e-16) from a uniform distribution based upon a two-sided KS test (D = 0.48) (Additional file 1: Figure S7).

### *Trans* regulatory hotspots

**Fig. 4:**
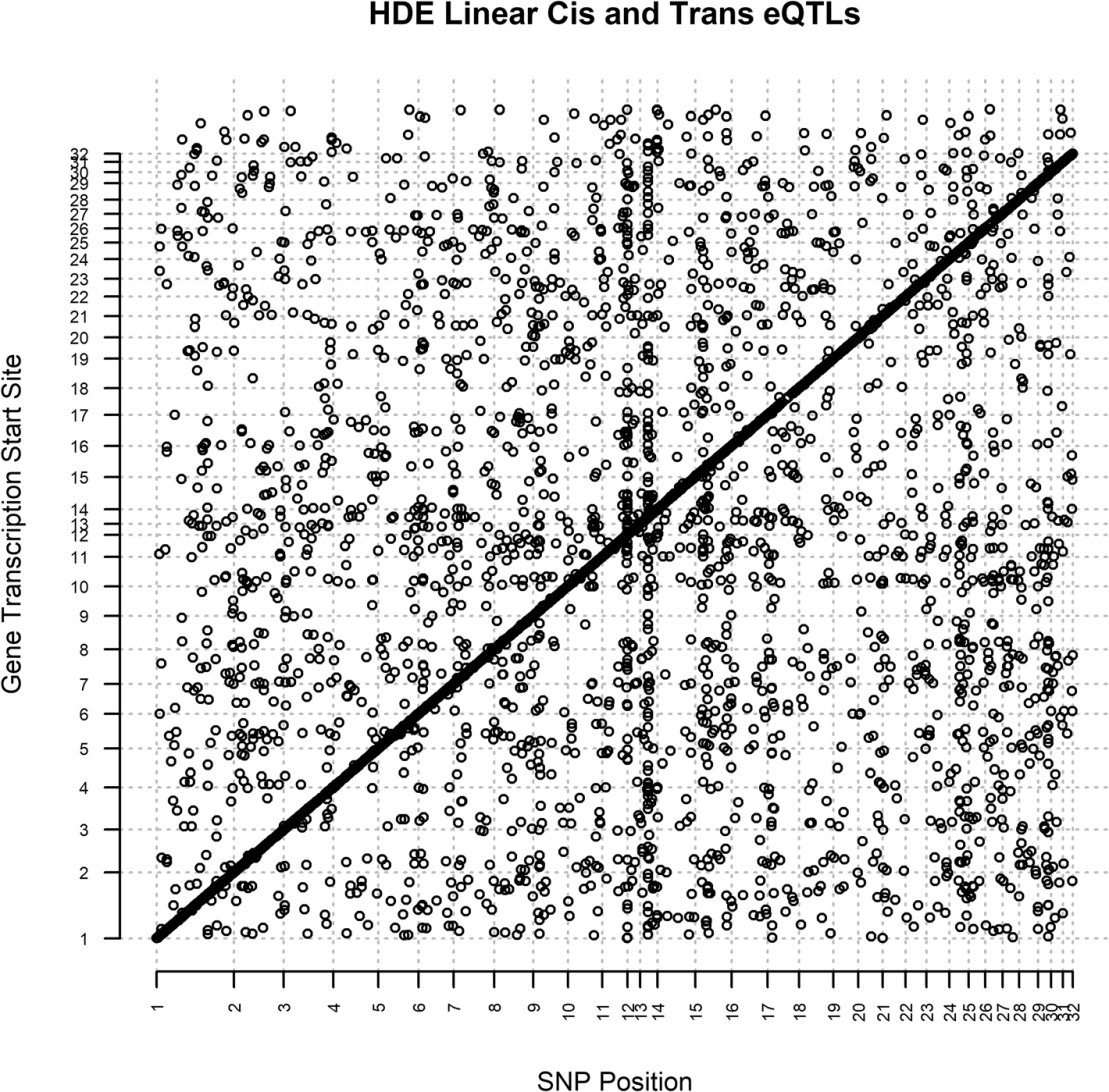
HDE9 *cis*- and *trans*- eQTLs from Matrix eQTL. X-axis is the genomic position of eSNPs while the y-axis is the genomic position of genes. Points were plotted for all eSNP/gene pairs for all high confidence significant eQTLs identified by Matrix eQTL for the HDE9 treatment. *Cis* eQTLs are present along the diagonal, while *trans* eQTLs are off the diagonal

**Fig. 5:**
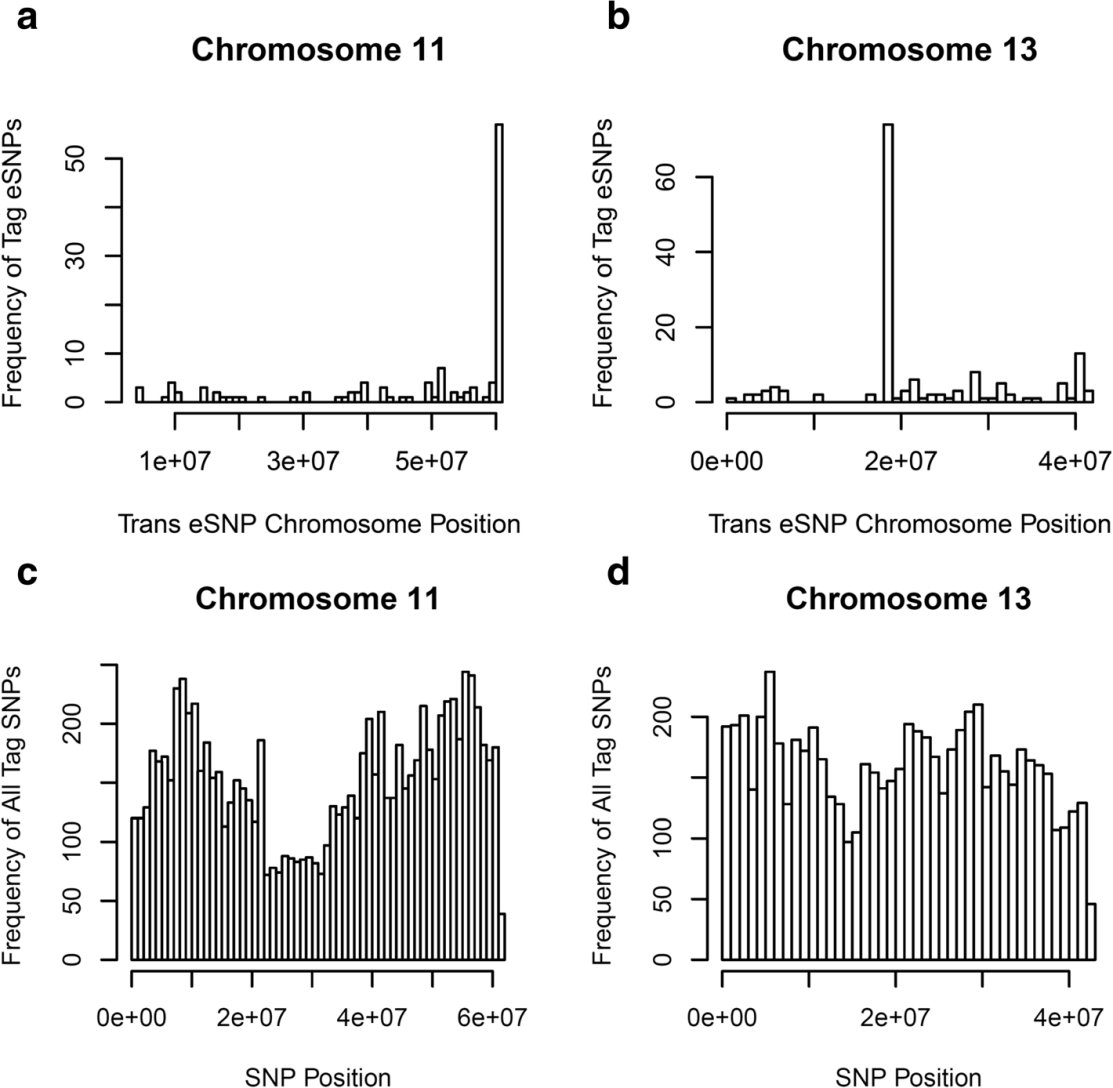
*Trans* regulatory hotspots have many genes regulated by one SNP (QTL). **a** & **b** Histograms show how often each SNP regulates a gene as a high confidence eQTL in *trans* for chromosomes 11 and 13. C) & D) Histograms show the frequency of tag SNPs across chromosomes 11 and 13 that were included in eQTL analyses. Only the eSNP with the lowest FDR for each gene was included in these analyses

The relationship between genomic positions of eSNPs and genes of high confidence eQTLs is summarized for treatment HDE in a *cis*-*trans* eQTL plot (Fig. 4). High densities of high confidence eSNPs can be visualized on chromosomes 11 and 13 indicating the presence of *trans* regulatory hotspots (Figs. 4, 5, Additional file 1: Figure S8). This evidence suggests that eSNPs including one SNP at position 60,892,596 on chromosome 11 (SNP: MNEc.2.11.60892596.PC) and one SNP at position 18,333,037 on chromosome 13 (SNP: MNEc.2.13.18333037.PC) regulate 44 and 74 genes respectively across the genome (Additional file 1: Tables S19-S20). These two SNPs were represented in significant *trans* eQTLs much more often than other SNPs throughout the genome, and on their respective chromosomes (Figs 4, 5, Additional file 1: Figure S7). These two SNPs were enriched in eQTLs relative to background distribution of all tag SNPs that were included in analyses (Fig. 5). SNP MNEc.2.11.60892596.PC lies within an intron of gene ubiquitin specific peptidase 22 (*USP22*). SNP MNEc.2.13.18333037.PC is intergenic lying 30,803 bps away from its nearest gene septin 14 (*SEPT14*).

Panther gene enrichment analysis for the *trans* regulatory hotspot on chromosome 11 found significant enrichment for GO processes involved with regulation of ion transmembrane transport (FDR = 3.16e-3), regulation of synapse assembly (FDR = 5.23e-3), and others when projected onto humans (Additional file 1: Table S21) [32]. Panther gene enrichment analysis of the 74 genes regulated in *trans* by SNP MNEc.2.13.18333037.PC on chromosome 13 shows significant enrichment for GO processes involving generation of neurons (FDR = 7.1e-8), regulation of nervous system development (FDR = 7.5e-8), regulation of neuron differentiation and projection development (FDR = 8.5e-7, and FDR = 1.31e-6), and others when genes names were projected onto humans (Additional file 1: Table S22).

### RAO associated SNPs and genes they regulate

We performed a GWAS on a filtered subset of (1,056,195) SNPs in 361 individuals (168 healthy and 193 with RAO) (Additional file 1: Table S23) (Methods). Similar to previous studies, we observed two RAO associated genomic regions on chromosome 13 (positions 23,001,364 and 32,843,309 – 33,525,948), however no SNPs were significant after genome wide multiple testing correction (Additional file 1: Figure S9) [12, 13]. We focused on MCK and HDE as these represent gene expression differences between baseline and RAO exacerbation. We report all significant *cis* eQTLs (FDR < 0.05) for the same 1,056,195 SNPs used in the GWAS for each treatment (Additional file 1: Tables S24-S27). Of the top fifteen RAO associated SNPs only four were also *cis* eSNPs in MCK (chr13.33502488, chr21.52625145, chr13.32844446, and chr13.32843309), and two in HDE (chr13.33502488, and chr28.3692072).

Three of the four MCK disease associated eSNPs resided on chromosome 13 from positions 32,843,309–33,502,488 in introns of Thioredoxin domain containing protein 11 (*TXNDC11*) and Class II Major Histocompatibility Complex Transactivator (*CIITA*), and were associated with gene expression of dexamethasone induced protein (*DEXI*). Two of these eSNPs (chr13.32843309, chr13.32844446) were in strong linkage disequilibrium (r^2^ = 0.87, D’ = 0.97), while the other SNPs were not in strong linkage disequilibrium (r^2^ < 0.75, D’ < 0.75) with another RAO associated eSNP (Additional file 1: Table S28). Therefore, a minimum of two RAO associated eQTLs regulate *DEXI* in MCK. We observed a significant loss of eQTL regulation of *DEXI* between MCK and HDE for SNPs chr13.32843309 (*p*-value = 3e-4) and chr13.32844446 (p-value = 5e-4), however the change in gene expression regulation was not significantly different between healthy and RAO horses (Additional file 1: Figure S10, Additional file 2). In HDE, one of the two eSNPs were the same as in MCK (chr13.33502488), however all eSNPs regulate different genes (*ATF7IP2*, and *GLIPR1L2*). We found no significant association between gene expression and disease status for the genes regulated by RAO associated eSNPs: *DEXI* (*p*-value > 0.83), or HDE: *ATF7IP2* (*p*-value = 0.53), or *GLIPR1L2* (*p*-value = 0.35) (Additional files 3 & 4).

## Discussion

Our results uncover thousands of SNPs, representing eQTLs, that have a significant linear association with gene expression in *cis* and *trans* in PBMCs from European Warmblood horses. We filtered results to improve reproducibility for future studies by implementing iterative KS tests, testing linear modeling assumptions, and detecting outlier individuals (S1 Text). We identified two *trans* regulatory hotspots in the HDE treatment. To identify RAO associated SNPs, we performed a GWAS with the highest density of SNPs (1,056,195) to date. Some of the top fifteen RAO associated SNPs were also eSNPs in treatments of PBMCs most relevant to RAO (MCK and HDE). However, none of the gene expression distributions (for genes regulated by these eSNPs) were significantly associated with RAO disease status. Therefore, we found no RAO associated SNPs that strongly influence disease status by regulating gene expression.

### *Trans* regulatory hotspots

*Trans* regulatory hotspots can regulate genes that are significantly enriched for one biological process [23]. This suggests that one variant could influence the regulation of gene expression of many genes from a common pathway. We identified two *trans* regulatory hotspots on chromosome 11 (SNP: MNEc.2.11.60892596.PC) and chromosome 13 (MNEc.2.13.18333037.PC) (Fig. 4, Additional file 1: Figure S4). We hypothesize that these two eQTLs each harbor variants regulating diffusible elements in *cis* that subsequently regulate many genes involved in transmembrane ion transport and neurological development.

### RAO associated SNPs and eQTLs

Current and previously published GWAS for RAO in horses have been underpowered (no SNPs reached genome wide significance after multiple testing correction) however these GWAS have discovered the strongest known associations to RAO in horses. Therefore, it could be valuable to identify genes these SNPs regulate, and test if the regulated gene expression is also associated to disease status in disease relevant tissues (ex: PBMCs containing lymphocytes and monocytes) after disease relevant treatments (MCK and HDE).

Four of the top fifteen disease-associated SNPs (representing a minimum of two eQTLs) were also eSNPs in MCK that regulated gene expression of DEXI and were within the same genomic region (chromosome 13: positions 32,843,309–33,502,488). This suggests that regulation of *DEXI* gene expression by these three SNPs could influence RAO in horses, or these SNPs have pleiotropic effects that independently influence RAO and gene expression regulation. In humans, eSNPs within the same homologous genomic region in introns of *CLEC16A* linked to asthma associated SNPs only regulate gene expression of *DEXI* which was also differentially expressed in human monocytes and bronchiolar lavage fluid (BALF) [18, 19, 33]. However, we found that in European Warmblood horses *DEXI* gene expression is a poor predictor of RAO disease status in PBMCs treated with MCK and HDE. *DEXI* is also not differentially expressed in horse PBMCs and the bronchial epithelium [9, 34]. Additionally, when PBMCs were treated with HDE we observed a treatment-induced loss of gene expression regulation of *DEXI*, however loss of regulation was observed both in healthy and RAO horses (Additional file 1: Figure S10 and Additional file 2). With the available evidence it appears that these SNPs might have pleiotropic effects that independently influence disease status and regulation of gene expression. Therefore, we could not explain the cause of the disease-associated signals to SNPs within this genomic region.

### Limitations

We studied a population of cell types (PBMCs), not a single cell type, therefore RNA expression could be influenced by inconsistent proportions of cell types between individuals. Our methods were capable of detecting thousands of high confidence eQTLs in horses. However, our methodology was not optimal for determining which eQTLs were shared across treatments. Single treatment analyses followed by intersecting lists of significant eQTLs underestimates the number of eQTLs that are shared across treatments [17, 35, 36]. A more accurate list of eQTLs shared across multiple treatments or unique to one treatment might be possible by jointly modeling treatments during eQTL analyses [17, 35]. However when our four treatments were jointly modeled, we observed possible inflation of shared eQTLs across all treatments (Additional file 1: S1 Text and Figure S6). Our methods determine if an eQTL is ‘significant’ and ‘present’ in a particular treatment of PBMCs. However, recently published tools can better leverage shared information (i.e. consistent or inconsistent weak eQTL signals that are individually not significant) across treatments, and can define how eQTLs differ across treatments by focusing on describing effect size differences rather than significance [36, 37]. These methods would be valuable in future analyses to quantify local polygenic effects on gene expression variation, or detect if a particular variant has major or minor effects on gene expression in eQTLs shared across treatments.

Many of the results reported here were based upon analyzing tag SNPs (Additional file 1: Tables S1–S22). Tag SNPs can represent the variation of multiple other SNPs with similar patterns of variation within a 100 kb window. Calling tag SNPs reduces the number of comparisons and increases statistical power, however during the selection process biologically important variants were excluded, including past RAO associated SNPs. Additionally, the genomic position of each associated tag SNP does not represent the location of other SNPs (removed from analyses) that it represents within the window.

Gene expression profiles are cell type and treatment specific [16, 17]. Therefore, analyzing other tissues might reveal enlightening results. It is also possible that the strongest RAO associated SNPs could change in future studies if higher density marker sets or different horse breeds are analyzed. The sample size for future GWAS must be increased to find significant associations to SNPs.

## Conclusions

Thousands of genetic variants regulate gene expression in PBMCs of European Warmblood horses in *cis* and *trans*. Most high confidence *cis* eSNPs are significantly enriched near the transcription start sites of their target genes. Two *trans* regulatory hotspots on chromosome 11 and 13 are significantly enriched for genes involved in transmembrane cell signaling and neurological development respectively when PBMCs are treated with HDE. We could not explain the cause of the disease associations to the top fifteen RAO associated SNPs. RAO associated SNPs that were also eSNPs in PBMCs treated with MCK and HDE in European Warmblood horses likely have pleiotropic effects that independently influence disease status and regulation of gene expression.

## Methods

### Samples, RNA extraction, and DNA extraction, PBMC treatment

Samples used in this study were previously collected, isolated, treated, and extracted as described in earlier publications [8, 11, 12, 38, 39]. Horses were kept in “low dust” environments before sample collection so that the RAO affected horses were in partial or full remission of RAO [8]. Horses were kept in stables with daily access to pasture all over Switzerland [8]. RAO horses received no prior treatment for RAO and a clinical exam was performed to rule out other systemic or localized infections [8].

DNA was previously extracted from PBMCs for two different studies [11, 12]. PBMCs were previously treated, RNA extracted, and RNA sequenced (RNAseq) by Pacholewska et al. [39]. Pacholewska et al. followed the density gradient centrifugation procedure from Hamza et al. to isolate PBMCs and followed the treatment of PBMCs and RNA extraction method from Lanz et al. [8, 38, 39]. European Warmblood horses were selected as the breed to study because of the two warmblood families (Fam1 and Fam2) with high incidences of RAO [40]. eQTL analyses used DNA and RNA from 82 European Warmblood horses (40 with RAO, and 42 healthy). Information on the independent variables (covariates) used is provided (Additional file 1: Tables S29-S30). Ages of RAO (mean = 16.7, min = 10, max = 24, units = years) and healthy controls (mean = 17.8, min = 6, max = 32, units = years) were comparable. These 82 horses belong to three familial cohorts, two half-sibling (half-sib) families with 17 individuals (Fam1) and 15 individuals (Fam2) respectively, and 50 unrelated horses (Additional file 1: Tables S29-S30). The sires of Fam1 and Fam2 both had RAO. Unrelated horses are not part of Fam1 or Fam2, and do not show strong patterns of population structure within the group (Additional file 1: Figure S3). Unrelated horses were defined as not having a common ancestor for at least two generations [12]. These 82 horses with SNP and RNAseq data represent a subsample of 379 European Warmblood horses with SNP data (Additional file 1: Table S30). 361 individuals (excluding HOARSI 2 individuals, and individuals with missing covariate information) from this larger cohort were used for imputation and GWAS (168 healthy, 193 RAO horses) (Additional file 1: Table S30). Additional file 1: Table S30 identifies which individuals were used in the GWAS and all eQTL analyses on the MCK, LPS, RCA, and HDE treatments.

### RAO phenotyping

The severity of the RAO phenotype was classified with the Horse Owner Assessed Respiratory Signs Index (HOARSI) index previously described by Ramseyer et al. and validated by Laumen et al. [41, 42]. Phenotype data was collected with a survey completed by horse owners. HOARSI indices range from one to four, representing horses with the following clinical signs: 1) no episodes of coughing or nasal discharge, 2) mucous nasal discharge and/or coughing, 3) abnormal breathing and/or regular or frequent coughing, and 4) abnormal breathing, and/or regular or frequent coughing, and poor performance [41]. Control horses were horses with a HOARSI index of one or two while case horses were horses with a HOARSI index of three or four. In the GWAS we excluded HOARSI 2 individuals to be consistent with past publications [13].

### RNA library preparation, RNA sequencing, and DNA 670 k SNP chip

RNA library preparation and RNA sequencing were previously described [39]. DNA was previously run on the Affymetrix Axiom Equine HD 670 k SNP chip [13]. Details of the Affymetrix® Axiom® Equine HD 670 k array design (MNEc670k) and imputation to 2 M SNPs (MNEc2M) was described by Schaefer et al. [30].

### SNP filtration and SNP imputation

Prior to imputation, SNPs with > 10% missing genotypes (7532 SNPs) and SNPs that deviated strongly from Hardy-Weinberg equilibrium (HWE) (HWE, *p* ≤ 0.0001; 29,720 SNPs) were removed [13]. SNPs were imputed with *Beagle* 4.1. The effective population size was set to 1000 while the rest of the parameters remained at their default values to impute individuals from 636,897 SNPs to 1,926,709 SNPs [28–30].

Accuracy of SNP imputation was measured by comparing 1,542,103 variants from the imputed dataset to variants overlapping the same genomic coordinates from genome wide sequence variants for two individuals (short read archive (SRA) Biosample IDs: SAMEA4351933 and SAMEA4351934). We measured 98.5% concordance for SAMEA4351933, and 98.6% for SAMEA4351934. Whole genome variants for individuals SAMEA4351933 and SAMEA4351934 were called with *Genome Analysis Toolkit (GATK)* best practices version 4. Genotypes were called with *VCFtools* v0.1.1.4 using option --012 [43]. Resulting genotypes were compared and concordance was calculated with a python script.

Tag SNPs were calculated on the imputed MNEc2M SNP set for the 82 individuals in this study using *FastTagger* v.1.0 [31]. We required tag SNPs to have a minor allele frequency (MAF) greater than 0.05, and the variation in the tag SNP must represent 99% of the genotypic diversity within each haplotype. Input files for *FastTagger* were created by converting the variant call format (vcf) file with python code, and chromosomes were analyzed one at a time (Fig. 1).

We obtained the filtered subset of imputed SNPs (1,056,195 SNPs) for the high resolution GWAS and eQTL analyses by filtering SNPs for the 379 individuals in the VCF file we deposited in the European Nucleotide Archive (ENA) (see Availability of data and material) with *VCFtools* v0.1.14 [43]. We required SNPs to be biallelic, and removed SNPs if MAF < 0.05, or SNPs (from control individuals only) deviated strongly from HWE (*p* ≤ 1e-6). Subsequently, we called genotypes with the --012 argument in VCFtools.

### Individual filtration

PCA analysis of the imputed SNP matrix revealed one outlier individual that did not cluster with our defined cohorts (unrelated horses, FAM1, and FAM2) and was removed from all downstream analyses. The biplots of the first three principal components based upon the genotype matrix shows the family structure, and that our covariate labels (Fam1, Fam2, and Unrelated) correctly identify the three cohorts (Additional file 1: Figure S3). The prcomp() function in R was used for PCA analyses. We used the check.marker() function in R library *GenABEL* (version 1.8–0) to determine if additional individuals should be removed from the analysis, due to incorrect covariate labels for sex, due to poor microarray genotype calling rates, or due to DNA contamination [44]. The check.marker() function did not remove any individuals based upon the following criterion: no individuals were identified as the incorrect sex (odds > 1000), no individuals had an excessively low genotyping call rate (< 0.1), no individuals had an excessively high identity by state (IBS, > = 0.95), or no individuals had an excessively high autosomal heterozygosity (FDR < 0.01).

### RNA sequence alignments

RNA sequences were previously aligned with *GEM* mapper (v1.6.2). Details of alignment settings are described by Pacholewska et al. [39]. Alignment files (BAMs) are available from the ENA database (http://www.ebi.ac.uk/ena/data/view/PRJEB7497).

### RNAseq counts: Gene features, counting, filtration, normalization, and variance stabilization

We defined gene features with the NCBI annotation (release 102) of the horse reference genome sequence EquCab2.0 (Assembly accession: GCF_000002305.2). We specified desired features to be all transcripts of genes with the transcriptsBy() function in the Bioconductor R library *GenomicFeatures* [45]. We counted the number of RNA reads that aligned to all transcripts of each gene with the summarizeOverlaps() function in the Bioconductor R library *GenomicAlignments* [45]. We simplified the count matrix to have one feature per gene, making genes (not transcripts of genes) the RNAseq count feature. In the summarizeOverlaps function we specified ‘mode = “Union”, singleEnd = FALSE, ignore.strand = TRUE, fragments = TRUE’. When any mode is specified in the summarizeOverlaps function each read is assigned to at most one feature. Therefore, mapping of a single read to multiple genes does not influence our read counts. We counted each treatment separately, and required genes to have at least one RNAseq read aligned to the gene in one individual which reduced the initial 26,707 genes to ~ 23,800 genes (Table 1). After this initial light filtration we normalized the reads with the counts(dds, normalized = TRUE) function in *DESeq2*. We normalized read counts to make them comparable across individuals, and then exported them to calculate a mean read count cutoff with the KS test statistic (described below). Genes with mean normalized read counts below this mean count threshold were removed from analysis for each treatment separately (Table 1). After trimming the number of genes, the gene expression raw counts were again normalized and then variance stabilized with the varianceStabilizingTransformation() in *DESeq2* once for each treatment separately (Table 1) [46].

### Kolmogorov-Smirnov (KS) test

We used the KS test to determine a read count threshold to remove lowly expressed genes (Text S1). We compared the trimmed and normalized gene expression profiles for all pairs of individuals for each treatment separately (described above). For each pair of individual in each treatment, we iteratively removed genes if the mean read counts were below a specified value (ranging from 0 to 300 with a step of 2), and stopped iterating if three criterion were satisfied (described below). Therefore, the gene set was reduced up to a maximum of 150 (300/2) times and up to a maximum of 150 separate KS tests were calculated (from which we collected D-statistics) for each pair of individuals. The total number of pairs of individuals were: MCK: 2628, LPS: 3240, RCA: 3160, HDE: 3321. For each iteration, we tested that the current D-statistic was lower than the starting (first) D-statistic, that the current D-statistic was lower than the next D-statistic, and that the current D-statistic is less than 0.0001 less than the starting (first) D-statistic. If these three criteria were satisfied then the iterative process stopped and the current cutoff value for one pair of individuals was recorded. After the iterative process completed for all pairs of samples we calculated the mean and median of all cutoff values (one for each pair of individuals).

We calculated KS statistics on the trimmed and normalized gene expression counts with the python module ks_2samp contained within python module *scipy*. We modified Python code that iteratively computes KS statistics for all pairs of normalized gene expression profiles of individuals from Farrell et al. (https://github.com/dmnfarrell/mirnaseq) to accept a gene expression count matrix as input [27]. We used this modified code to produce all cutoff values.

### KS test determines significantly different distribution

The KS test was applied twice more: 1) for distances between a genes transcription start site and associated eSNP from the HDE treatment, and 2) for each of the 993 eQTLs (with lowest FDR values) with a different eSNP but the same gene, we calculated the distance between each pair of differing eSNPs for each gene. We then used the ks.test() function in R to calculate two-sided KS test, and compare the distribution of distances to that of a uniform distribution.

### Accounting for unknown batch effects

*Probabilistic estimation of expression residuals* (*PEER*) (v1.3) was run with default parameters and adding model.setAdd_mean(True) separately for each treatment of PBMCs to account for batch effects in gene expression matrices for the linear additive model analysis in Matrix eQTL and our independent linear modeling of additive eQTLs in R (Text S1) [47, 48]. We included known sources of possible confounding covariation as covariates (sex, age, Fam1 status, and Fam2 status, case/control status) and input the *DESeq2* trimmed, normalized, and variance stabilized RNAseq counts to calculate the residuals of gene expression in *PEER*.

We calculated surrogate variables with *Surrogate Variable Analysis* (*SVA*) (v3.22.0) to account for batch effects prior to testing if gene expression predicted RAO disease status [49, 50]. Surrogate variables are covariates estimated from the gene expression matrix that can be included in downstream analyses to account for common sources of latent (unwanted) variation, while protecting variation between specified categorical variables [49, 50]. Surrogate variables can represent biased variation in the gene expression matrix that were introduced through changes in methodological procedures, date of sample processing, or changes to reagents between subsets of samples included in the linear model. We included known sources of possible confounding covariation as covariates (sex, age, Fam1 status, and Fam2 status), and protected variation in the disease status covariate. We input the *DESeq2* normalized and variance stabilized RNAseq counts and ran the sva() function (to avoid the log transformation in the svaseq() function) to calculate surrogate variables [46]. These surrogate variables were then included as independent variables in downstream analyses to account for batch effects.

### Additive linear model

Linear model equation for the Linear models in *Matrix eQTL* and our independent linear modeling of additive eQTLs in R:

1 $$ \mathbf{y}=\mu +\mathbf{m}u+\varepsilon $$

In equation one, **y** is the dependent variable representing the residuals of the trimmed, normalized, and variance stabilized gene expression counts with known and unknown sources of confounding variation regressed out. In equation one, *μ* is the intercept, **m** is a vector of SNP marker genotypes (0, 1, or 2 for each individual: 0 is homozygous reference allele, 1 is heterozygous, and 2 is homozygous alternative allele), *u* is the SNP marker effect, and ε is the residuals.

### *Cis* and *trans* eQTL discovery with *Matrix eQTL*

eQTLs were *cis* if the SNP location was within 1 Mb upstream or downstream of a genes’ transcription start site by setting cisDist = 1e6 (resulting in a 2 Mb *cis* window). We input the 347,455 tag SNPs into *Matrix eQTL* and excluded the X-chromosome. For ‘modelLINEAR’ we input the residuals of gene expression calculated in *PEER* (see above). Each treatment of PBMCs (MCK, LPS, RCA, HDE) was run separately. *Matrix eQTL* was run with ‘modelLINEAR’ for *cis* and *trans* eQTLs. False discovery rates (FDRs) following the Benjamini Hochberg method were calculated by *Matrix eQTL* [25, 51]. We set a FDR threshold at 0.05, and therefore eQTLs with FDR < 0.05 were considered statistically significant. We generated histograms and QQ-plots for *cis* and *trans* eQTLs with *Matrix eQTL* prior to high confidence eQTL filtration. We selected the eQTL with the lowest FDR for each gene to represent the best gene/eSNP pair. We classified eQTLs as significant or not significant, however we additionally classified each eQTL as ‘high confidence’ or ‘low confidence’ (see below).

### Reproducing linear models in R, diagnostic plots, outlier detection, and validating linear model assumptions

Multiple linear regressions of *Matrix eQTL* were reproduced in R (v3.3.3) for each significant eQTL identified from *Matrix eQTL* using the lm() function. The linear regression model to reproduce ‘modelLINEAR’ is shown as eq. (1). We used the PEER gene expression residuals as the dependent variable and genotype as the independent variable (eq. 1).

### Detecting outlier individuals and validating linear model assumptions

If an eQTL had outlier individuals, or violated linear modeling assumptions (see below) the eQTL was placed in a separate ‘low confidence’ category (Additional file 1: Figure S5 and S11) (Additional file 1: Tables S2, S4, S6, S8, S10, S12, S14, and S16). We applied outlier detection for all significant *cis* and *trans* eQTL (gene/SNP pair) results from *Matrix eQTL*. Individuals were detected as outliers in R by calculating Cook’s Distance and leverage of all individuals for each eQTL. Individuals with high Cook’s distance > 0.5 and *leverage* > 2(*p*/*n*) (p = number of linear model coefficients (parameters) and n = sample size) were determined to be outliers [52, 53].

### Checking linear model assumptions

Linear model assumptions were tested with the r package *Global Validation of Linear Models Assumptions* (*gvlma*) (v1.0.0.2) and using the gvlma() function (alphalevel = 0.05) [54]. We categorized eQTLs as ‘low confidence’ if the global statistic was not accepted. The global test statistic combines separate tests assessing skewness, kurtosis, link function, and heteroscedasticity as a global omnibus statistic [54].

### Cis eQTL discovery with *eQTLBMA*

The four treatments of PBMCs were jointly modeled with Bayesian methods for eQTL detection in *eQTLBMA* (v1.3.1) (14). The gene expression residuals calculated by *PEER* (see above) were used for each treatment. In the eqtlbma_bf command we set ‘--error hybrid’ to designate that some sample treatments were from the same individuals, but some individuals were not shared across all treatments. This option assumes that the error covariance matrix is correlated between treatments, but calculates this matrix between all pairs of treatments with only individuals common to each treatment (*eQTLBMA* manual). We designated cis eQTLs as SNPs within 1000,000 bases upstream or downstream of gene transcription start sites by setting ‘--cis 1000000’ (resulting in a 2 Mb *cis* window). We followed the EBF procedure in the *eQTLBMA* manual to estimate the probability for a gene to have no eQTL in any treatment (π0) and then calculated the posterior probabilities for each different configuration with *eQTLBMA*. Our code used for *eQTLBMA* is provided at: https://github.com/VCMason and an outline of how *eQTLBMA* fits into the overall workflow is shown in Additional file 1: Figure S1.

### Two proportion Z-test

Using the two-proportion z-test we calculated a significant difference in the proportion of eSNPs in genic (5’-UTR, exons, introns, 3’-UTR) regions between *cis* and *trans* genic eSNPs (Additional file 1: Table S17). We limited the analysis to only high confidence eSNPs (with the lowest FDR) for each gene. We calculated the numbers of *cis* and *trans* eSNPs lying in genic regions (5’ UTR, exons, introns, 3’ UTR) with a python script and divided them by the total numbers of *cis* or *trans* eSNPs in in the HDE treatment to obtain the proportions.

### GWAS

Using 1,056,195 SNPs we ran a mixed-effects logistic regression model in R with the glmer() function from the *lme4* library (version 1.1–14) to search for the genotypic association to disease status. Disease status was coded as a binary variable (0 == control, and 1 == case). We included genotype (0 == homozygous reference, 1 == heterozygous, or 2 == homozygous alternative), sex (0 == male, 1 == female), Fam1 (0 == Fam2 or unrelated, 1 == Fam1) and Fam2 (0 == Fam1 or unrelated, 1 == Fam2) to account for population structure, and age (continuous numeric values in years) as covariates in equation two.

2 $$ \mathbf{y}\sim \mathbf{X}\beta +\mathbf{m}u+\varepsilon $$

In equation two, **y** represents the dependent variable disease status, **X** is an incidence matrix for fixed effects intercept, age, sex, Fam1, and Fam2, *β* is the solution for the fixed effects intercept, age (in years), sex, Fam1, and Fam2, **m** is a vector of SNP marker genotypes, *u* is the SNP marker effect, and *ε* are the residuals. We removed individuals with missing phenotype data for any covariate. We ranked GWAS results by *p*-values for fixed effect genotype.

### Quantifying linkage disequilibrium between SNPs

We extracted all SNPs imputed and phased from an interval 1 Mb upstream and downstream (positions: 31843309–33,843,309 on chromosome 13) from RAO associated SNP MNEc.2.13.32843309.PC with *VCFtools* v0.1.14. We calculated the haplotype r^2^, D, and D’ (r^2^ = correlation between haplotypes, D = linkage coefficient, D’ = normalized linkage coefficient) for pairwise combinations of RAO associated with the --hap-r2 argument for this genomic interval for the 361 individuals analyzed in the GWAS. We filtered the results to SNPs associated with RAO that were also eSNPs in MCK (Additional file 1: Table S28).

## Additional files

Additional file 1: **Figure S1.** Minimum D-statistics determine mean read count cutoffs. **Figure S2.** PCA plots of normalized variance stabilized RNAseq counts after KS test filter. **Figure S3.** PCA plots of 1,056,195 SNP genotypes and colored by cohort. **Figure S4.** Matrix eQTL histograms and QQ-plots for all p-values for all *cis* and *trans* eQTL analyses using tag SNPs for the MCK1 treatment. **Figure S5.** Low confidence *cis* eQTLs. **Figure S6.** Joint modeling with eQTLBMA with possible overestimation of shared eQTLs across all PBMC treatments. **Figure S7.** Distance between eSNPs with the lowest FDR values per gene is small. **Figure S8.**. Enrichment of SNPs in *trans* regulatory hotspots genome wide. **Figure S9.** GWAS for RAO. **Figure S10.** Loss of *DEXI* gene expression regulation in HDE. **Figure S11.** *Cis* trans eQTL plot for all eQTLs for treatment HDE9. **Table S1.** High confidence additive linear *cis* eQTLs from the MCK treatment. **Table S2.** Low confidence additive linear *cis* eQTLs from the MCK treatment. **Table S3.** High confidence additive linear *trans* eQTLs from the MCK treatment. **Table S4.** Low confidence additive linear *trans* eQTLs from the MCK treatment. **Table S5.** High confidence additive linear *cis* eQTLs from the LPS treatment. **Table S6.** Low confidence additive linear *cis* eQTLs from the LPS treatment. **Table S7.** High confidence additive linear *trans* eQTLs from the LPS treatment. **Table S8.** Low confidence additive linear *trans* eQTLs from the LPS treatment. **Table S9.** High confidence additive linear *cis* eQTLs from the RCA treatment. **Table S10.** Low confidence additive linear *cis* eQTLs from the RCA treatment. **Table S11.** High confidence additive linear *trans* eQTLs from the RCA treatment. The eQTLs reported are limited to one eQTL per gene, representing the eSNP with the lowest FDR value for each gene. **Table S12.** Low confidence additive linear *trans* eQTLs from the RCA treatment. **Table S13.** High confidence additive linear *cis* eQTLs from the HDE treatment. **Table S14.** Low confidence additive linear *cis* eQTLs from the HDE treatment. **Table S15.** High confidence additive linear *trans* eQTLs from the HDE treatment. **Table S16.** Low confidence additive linear *trans* eQTLs from the HDE treatment. **Table S17.** Two proportion z-test calculation. **Table S18.** 4157 significant eQTLs discovered with eQTLBMA. **Table S19.** *Trans* eQTL results for the *trans* regulatory hotspot on chromosome 11 (SNP MNEc.2.11.60892596.PC). **Table S20.** *Trans* eQTL results for the *trans* regulatory hotspot on chromosome 13 (SNP MNEc.2.13.18333037.PC). **Table S21.** Panther gene enrichment GO process results for genes regulated by the *trans* regulatory hotpot on chromosome 11 (MNEc.2.11.60892596.PC). **Table S22.** Panther gene enrichment GO process results for genes regulated by the *trans* regulatory hotpot on chromosome 13 (MNEc.2.13.18333037.PC). **Table S23.** GWAS results. **Table S24.** All significant *cis* eQTLs for the MCK treatment. **Table S25.** All significant *cis* eQTLs for the LPS treatment. **Table S26.** All significant *cis* eQTLs for the RCA treatment. **Table S27.** All significant *cis* eQTLs for the HDE treatment. **Table S28.** Linkage disequilibrium and allele frequencies between RAO associated SNPs on chromosome 13 positions 32,843,309 – 33,502,488. **Table S29**. Sample information for 82 individuals used in eQTL analyses. **Table S30.** Sample information for all 379 individuals. (ZIP 51963 kb)

Additional file 2: Linear mixed models jointly modeling MCK and HDE. Linear mixed models with random intercepts for each individual model the association between the top fifteen RAO associated SNPs that were also eSNPs in either MCK or HDE (chr13.32843309, chr13.32844446, chr13.33460982, chr13.33502488, chr28.3692072, chr21.52625145) and the gene expression of the genes they regulated (*DEXI*, *NSUN2*, *ATF7IP2*, *GLIPR1L2*) with reduced maximum likelihood (REML) and maximum likelihood (ML). An R markdown document that generated this html file is available on GitHub: https://github.com/VCMason. (HTML 5545 kb)

Additional file 3: Association of *DEXI* and *NSUN2* gene expression to RAO disease status. Html output of an R markdown document. The file contains two multiple logistic regressions and one simple logistic regression showing the association between *DEXI* gene expression and disease status. Multiple logistic regression with known confounders as independent variables, and the simple logistic regression only has the independent variable of interest (*DEXI* or *NSUN2* gene expression) as the single covariate. An R markdown document that generated this html file is available on GitHub: https://github.com/VCMason. (HTML 844 kb)

Additional file 4: Association of *ATF7IP2*, and *GLIPR1L2* gene expression to RAO disease status. Html output of an R markdown document. The file contains one multiple logistic regression for each gene *ATF7IP2*, and *GLIPR1L2*. These models quantify the association between gene expression in *ATF7IP2*, or *GLIPR1L2* and disease status. Four significant surrogate variables were calculated for the HDE treatment by SVA and therefore none were included in the model. An R markdown document that generated this html file is available on GitHub: https://github.com/VCMason. (HTML 866 kb)

## Abbreviations

ASE: Allele specific expression
ENA: European Nucleotide Archive
eQTL: Expression quantitative trait loci
*eQTLBMA*: *eQTL by Bayesian Model Averaging*
eSNP: eQTL SNP
EVA: European Variant Archive
FDR: False discovery rate
*GATK*: *Genome Analysis Toolkit*
GWAS: Genome wide association study
HDE: Hay dust extract treatment
HOARSI: Horse Owner Assessed Respiratory Signs Index
HWE: Hardy-Weinberg equilibrium
KS: Kolmogorov-Smirnov
LPS: Lipopolysaccharide treatment
MAF: Minor allele frequency
MCK: No treatment, (i.e. mock treatment)
miRNA: Micro-ribonucleic acid
PBMCs: Peripheral blood mononuclear cells
PCA: Principle component analysis
*PEER*: *Probabilistic estimation of expression residuals*
RAO: Recurrent airway obstruction
RCA: Recombinant cyathostomin antigen treatment
SNP: Single nucleotide polymorphism
SRA: Short Read Archive
*SVA*: *Surrogate Variable Analysis*
TFs: Transcription factors
VCF: Variant call format

## References

[1] Hotchkiss JW, Reid SWJ, Christley RM (2007). A survey of horse owners in Great Britain regarding horses in their care. Part 1: horse demographic characteristics and management. Equine Vet J.

[2] Bullone M, Lavoie JP. Asthma “of horses and men”-how can equine heaves help us better understand human asthma immunopathology and its functional consequences? Mol Immunol. 2015;66:97–105. 10.1016/j.molimm.2014.12.005. 10.1016/j.molimm.2014.12.005 25547716

[3] Gerber V, Tessier C, Marti E (2015). Genetics of upper and lower airway diseases in the horse. Equine Vet J.

[4] Bullone M, Lavoie JP (2016). Recurrent airway obstruction and summer pasture-associated obstructive pulmonary disease. Equine Clinical Immunology.

[5] Leclere M, Lavoie-Lamoureux A, Gélinas-Lymburner É, David F, Martin JG, Lavoie JP (2011). Effect of antigenic exposure on airway smooth muscle remodeling in an equine model of chronic asthma. Am J Respir Cell Mol Biol.

[6] Leclere M, Lavoie-Lamoureux A, Lavoie JP (2011). Heaves, an asthma-like disease of horses. Respirology.

[7] Leclere M, Lavoie-Lamoureux A, Joubert P, Relave F, Setlakwe EL, Beauchamp G (2012). Corticosteroids and antigen avoidance decrease airway smooth muscle mass in an equine asthma model. Am J Respir Cell Mol Biol.

[8] Lanz S, Gerber V, Marti E, Rettmer H, Klukowska-Rötzler J, Gottstein B, et al. Effect of hay dust extract and cyathostomin antigen stimulation on cytokine expression by PBMC in horses with recurrent airway obstruction. Vet Immunol Immunopathol. 2013;155:229–37. 10.1016/j.vetimm.2013.07.005. 10.1016/j.vetimm.2013.07.005 23972861

[9] Pacholewska A, Jagannathan V, Drögemüller M, Klukowska-Rötzler J, Lanz S, Hamza E, et al. Impaired cell cycle regulation in a natural equine model of asthma. PLoS One. 2015;10:1–23. 10.1371/journal.pone.0136103. 10.1371/journal.pone.0136103 PMC4546272 26292153

[10] Pirie RS (2014). Recurrent airway obstruction: a review. Equine Vet J.

[11] Swinburne JE, Bogle H, Klukowska-Rötzler J, Drögemüller M, Leeb T, Temperton E (2009). A whole-genome scan for recurrent airway obstruction in warmblood sport horses indicates two positional candidate regions. Mamm Genome.

[12] Shakhsi-Niaei M, Klukowska-Rötzler J, Drögemüller C, Swinburne J, Ehrmann C, Saftic D (2012). Replication and fine-mapping of a QTL for recurrent airway obstruction in European warmblood horses. Anim Genet.

[13] Schnider D, Rieder S, Leeb T, Gerber V, Neuditschko M. A genome-wide association study for equine recurrent airway obstruction in European warmblood horses reveals a suggestive new quantitative trait locus on chromosome 13. Anim Genet. 2017;48:691–3. 10.1111/age.12583. 10.1111/age.12583 28737212

[14] Albert FW, Kruglyak L. The role of regulatory variation in complex traits and disease. Nat Rev Genet. 2015;16:197–212. 10.1038/nrg3891. 10.1038/nrg3891 25707927

[15] Thibodeau SN, French AJ, McDonnell SK, Cheville J, Middha S, Tillmans L, et al. Identification of candidate genes for prostate cancer-risk SNPs utilizing a normal prostate tissue eQTL data set. Nat Commun. 2015;6:8653. 10.1038/ncomms9653. 10.1038/ncomms9653 PMC4663677 26611117

[16] Nica AC, Dermitzakis ET. Expression quantitative trait loci: present and future. Philos Trans R Soc B Biol Sci. 2013;368:20120362. 10.1098/rstb.2012.0362. 10.1098/rstb.2012.0362 PMC3682727 23650636

[17] Peters JE, Lyons PA, Lee JC, Richard AC, Fortune MD, Newcombe PJ, et al. Insight into genotypephenotype associations through eQTL mapping in multiple cell types in health and immune-mediated disease. PLoS Genet. 2016;12. 10.1371/journal.pgen.1005908. 10.1371/journal.pgen.1005908 PMC4807835 27015630

[18] Ferreira MAR, Matheson MC, Tang CS, Granell R, Wei Ang JH, Kiefer AK (2014). Genome-wide association analysis identifies 11 risk variants associated with the asthma with hay fever phenotype. J Allergy Clin Immunol.

[19] Li X, Hastie AT, Hawkins GA, Moore WC, Ampleford EJ, Milosevic J (2015). eQTL of bronchial epithelial cells and bronchial alveolar lavage deciphers GWAS-identified asthma genes. Allergy.

[20] Zhu Z, Zhang F, Hu H, Bakshi A, Robinson MR, Powell JE, et al. Integration of summary data from GWAS and eQTL studies predicts complex trait gene targets. Nat Genet. 2016;48:481–7. 10.1038/ng.3538. 10.1038/ng.3538 27019110

[21] Yu C-H, Pal LR, Moult J. Consensus genome-wide expression quantitative trait loci and their relationship with human complex trait disease. Omi A J Integr Biol. 2016;20:400–14. 10.1089/omi.2016.0063. 10.1089/omi.2016.0063 PMC4960478 27428252

[22] Huang Y-T, Liang L, Moffatt MF, Cookson WOCM, Lin X. iGWAS: Integrative Genome-Wide Association Studies of Genetic and Genomic Data for Disease Susceptibility Using Mediation Analysis. 2015;28:1304–1314. 10.1002/gepi.21905 PMC4544880 25997986

[23] Brynedal B, Choi JM, Raj T, Bjornson R, Stranger BE, Neale BM (2017). Large-scale trans-eQTLs affect hundreds of transcripts and mediate patterns of transcriptional co-regulation. Am J Hum Genet.

[24] Yao C, Joehanes R, Johnson AD, Huan T, Liu C, Freedman JE, et al. Dynamic role of trans regulation of gene expression in relation to complex traits. Am J Hum Genet. 2017;100:571–80. 10.1016/j.ajhg.2017.02.003. 10.1016/j.ajhg.2017.02.003 PMC5384035 28285768

[25] Shabalin AA (2012). Matrix eQTL: ultra fast eQTL analysis via large matrix operations. Bioinformatics.

[26] Koh W, Sheng CT, Tan B, Lee QY, Kuznetsov V, Kiang LS, et al. Analysis of deep sequencing microRNA expression profile from human embryonic stem cells derived mesenchymal stem cells reveals possible role of let-7 microRNA family in downstream targeting of hepatic nuclear factor 4 alpha. BMC Genomics. 2010;11(Suppl 1):S6. 10.1186/1471-2164-11-S1-S6. 10.1186/1471-2164-11-S1-S6 PMC2822534 20158877

[27] Farrell D, Shaughnessy RG, Britton L, MacHugh DE, Markey B, Gordon SV. The identification of circulating MiRNA in bovine serum and their potential as novel biomarkers of early mycobacterium avium subsp paratuberculosis infection. PLoS One. 2015;10:1–22. 10.1371/journal.pone.0134310. 10.1371/journal.pone.0134310 PMC4517789 26218736

[28] Browning SR, Browning BL. Rapid and accurate haplotype phasing and missing-data inference for whole-genome association studies by use of localized haplotype clustering. Am J Hum Genet. 2007;81:1084–97. 10.1086/521987. 10.1086/521987 PMC2265661 17924348

[29] Browning BL, Browning SR (2016). Genotype imputation with millions of reference samples. Am J Hum Genet.

[30] Schaefer RJ, Schubert M, Bailey E, Bannasch DL, Barrey E, Bar-Gal GK (2017). Developing a 670k genotyping array to tag ~2M SNPs across 24 horse breeds. BMC Genomics.

[31] Liu G, Wang Y, Wong L, Johnson G, Patil N, Gabriel S, et al. FastTagger: an efficient algorithm for genome-wide tag SNP selection using multi-marker linkage disequilibrium. BMC Bioinformatics. 2010;11:66. 10.1186/1471-2105-11-66. 10.1186/1471-2105-11-66 PMC3098109 20113476

[32] Mi H, Huang X, Muruganujan A, Tang H, Mills C, Kang D (2017). PANTHER version 11: expanded annotation data from gene ontology and Reactome pathways, and data analysis tool enhancements. Nucleic Acids Res.

[33] Davison LJ, Wallace C, Cooper JD, Cope NF, Wilson NK, Smyth DJ (2012). Long-range DNA looping and gene expression analyses identify DEXI as an autoimmune disease candidate gene. Hum Mol Genet.

[34] Tessier L, Côté O, Clark ME, Viel L, Diaz-Méndez A, Anders S, et al. Impaired response of the bronchial epithelium to inflammation characterizes severe equine asthma. BMC Genomics. 2017;18:708. 10.1186/s12864-017-4107-6. 10.1186/s12864-017-4107-6 PMC5591550 28886691

[35] Flutre T, Wen X, Pritchard J, Stephens M (2013). A statistical framework for joint eQTL analysis in multiple tissues. PLoS Genet.

[36] Urbut SM, Wang G, Stephens M. Flexible statistical methods for estimating and testing effects in genomic studies with multiple conditions. bioRxiv. 2016. 10.1101/096552. 10.1038/s41588-018-0268-8 PMC6309609 30478440

[37] Casale FP, Horta D, Rakitsch B, Stegle O, Abecasis G, Salem R. Joint genetic analysis using variant sets reveals polygenic gene-context interactions. PLoS Genet. 2017;13:e1006693. 10.1371/journal.pgen.1006693. 10.1371/journal.pgen.1006693 PMC5398484 28426829

[38] Hamza E, Doherr MG, Bertoni G, Jungi TW, Marti E (2007). Modulation of allergy incidence in icelandic horses is associated with a change in IL-4-producing T cells. Int Arch Allergy Immunol.

[39] Pacholewska A, Drögemüller M, Klukowska-Rötzler J, Lanz S, Hamza E, Dermitzakis ET (2015). The transcriptome of equine peripheral blood mononuclear cells. PLoS One.

[40] Gerber V, Baleri J, Klukowska-Rötzler J, Swinburne JE, Dolf G. Mixed Inheritance of Equine Recurrent Airway Obstruction. 2009;23:626–630. 10.1111/j.1939-1676.2009.0292.x 19645845

[41] Ramseyer A, Gaillard C, Burger D, Straub R, Jost U, Boog C, et al. Effects of genetic and environmental factors on chronic lower airway disease in horses. J Vet Intern Med. 2007;21:149. 10.1892/0891-6640(2007)21[149:EOGAEF]2.0.CO;2. 17338163

[42] Laumen E, Doherr MG, Gerber V (2010). Relationship of horse owner assessed respiratory signs index to characteristics of recurrent airway obstruction in two warmblood families. Equine Vet J.

[43] Danecek P, Auton A, Abecasis G, Albers CA, Banks E, DePristo MA (2011). The variant call format and VCFtools. Bioinformatics.

[44] Aulchenko YS, Ripke S, Isaacs A, van Duijn CM (2007). GenABEL: an R library for genome-wide association analysis. Bioinformatics.

[45] Lawrence M, Huber W, Pagès H, Aboyoun P, Carlson M, Gentleman R (2013). Software for computing and annotating genomic ranges. PLoS Comput Biol.

[46] Love MI, Huber W, Anders S (2014). Moderated estimation of fold change and dispersion for RNA-seq data with DESeq2. Genome Biol.

[47] Stegle O, Parts L, Durbin R, Winn J (2010). A bayesian framework to account for complex non-genetic factors in gene expression levels greatly increases power in eQTL studies. PLoS Comput Biol.

[48] Stegle O, Parts L, Piipari M, Winn J, Durbin R (2012). Using probabilistic estimation of expression residuals (PEER) to obtain increased power and interpretability of gene expression analyses. Nat Protoc.

[49] Leek JT, Johnson WE, Parker HS, Jaffe AE, Storey JD (2012). The SVA package for removing batch effects and other unwanted variation in high-throughput experiments. Bioinformatics.

[50] Leek JT (2014). Svaseq: removing batch effects and other unwanted noise from sequencing data. Nucleic Acids Res.

[51] Benjamin Y, Hochberg Y. Controlling the false discovery rate: a practical and powerful approach to multiple testing. J R Stat Soc Ser B. 1995;57:289–300. http://www.jstor.org/stable/2346101.

[52] Cook RD (1977). Detection of influential observation in linear regression. Technometrics.

[53] Crawley MJ (2012). Statistical Modelling. The R book.

[54] Peña EA, Slate EH (2006). Global validation of linear model assumptions. J fo Am Stat Assoc.

