# Supplementary material for: eQTL discovery and their association with severe equine asthma in European Warmblood horses
Source: BMC Genomics. 2018 Aug 2;19:581. doi: 10.1186/s12864-018-4938-9 (PMC6090848; doi:10.1186/s12864-018-4938-9)
Supplement: Supplementary file 1 — Figure S1. Minimum D-statistics determine mean read count cutoffs. Figure S2. PCA plots of normalized variance stabilized RNAseq counts after KS test filter. Figure S3. PCA plots of 1,056,195 SNP genotypes and colored by cohort. Figure S4. Matrix eQTL histograms and QQ-plots for all p-values for all cis and trans eQTL analyses using tag SNPs for the MCK1 treatment. Figure S5. Low confidence cis eQTLs. Figure S6. Joint modeling with eQTLBMA with possible overestimation of shared eQTLs across all PBMC treatments. Figure S7. Distance between eSNPs with the lowest FDR values per gene is small. Figure S8.. Enrichment of SNPs in trans regulatory hotspots genome wide. Figure S9. GWAS for RAO. Figure S10. Loss of DEXI gene expression regulation in HDE. Figure S11. Cis trans eQTL plot for all eQTLs for treatment HDE9. Table S1. High confidence additive linear cis eQTLs from the MCK treatment. Table S2. Low confidence additive linear cis eQTLs from the MCK treatment. Table S3. High confidence additive linear trans eQTLs from the MCK treatment. Table S4. Low confidence additive linear trans eQTLs from the MCK treatment. Table S5. High confidence additive linear cis eQTLs from the LPS treatment. Table S6. Low confidence additive linear cis eQTLs from the LPS treatment. Table S7. High confidence additive linear trans eQTLs from the LPS treatment. Table S8. Low confidence additive linear trans eQTLs from the LPS treatment. Table S9. High confidence additive linear cis eQTLs from the RCA treatment. Table S10. Low confidence additive linear cis eQTLs from the RCA treatment. Table S11. High confidence additive linear trans eQTLs from the RCA treatment. The eQTLs reported are limited to one eQTL per gene, representing the eSNP with the lowest FDR value for each gene. Table S12. Low confidence additive linear trans eQTLs from the RCA treatment. Table S13. High confidence additive linear cis eQTLs from the HDE treatment. Table S14. Low confidence additive linear cis eQTLs from the HDE [file 12864_2018_4938_MOESM1_ESM.zip › S1_Text.Rev1.NoTrackChanges.docx]

**Supplemental Material:**

**eQTL Discovery and their Association with Severe Equine Asthma in European Warmblood Horses**

Victor C. Mason^1^, Robert Schaefer^2^, Molly McCue^2^, Tosso Leeb^3^, Vinzenz Gerber^1^

**1** Department of Clinical Veterinary Medicine, Swiss Institute of Equine Medicine, Vetsuisse Faculty, University of Bern, and Agroscope, Länggassstrasse 124, 3012 Bern, Switzerland **2** Department of Veterinary Population Medicine, University of Minnesota, 1365 Gortner Ave, Saint Paul, MN, 55108, USA **3** Department of Clinical Research and Veterinary Public Health, Institute of Genetics, Vetsuisse Faculty, University of Bern, Bremgartenstrasse 109A, 3012 Bern, Switzerland

**Increasing reproducibility**

**KS Test Gene Filtration.** Poisson noise affects genes with low RNAseq counts more than at higher counts [1]. We removed genes with mean trimmed normalized gene expression values less than a cutoff value for each treatment (Table 1). Cutoffs were determined by iterative testing of the Kolmogorov-Smirnov (KS) statistic between all pairs of individuals [2, 3]. A cutoff value between one pair of individuals represents the point at which the distribution of gene expression counts are most similar between individuals, and therefore likely represents the set of genes that would be more easily reproducible in subsequent studies. When cutoffs are averaged across all pairs of individuals this represents the mean cutoff value that selects for a set of genes with most similar gene count distributions across all individuals. Removal of genes with low RNAseq counts, that have higher variance in RNA seq count matrices, makes the RNAseq count distributions more comparable across samples to improve reproducibility of results.

**High Confidence eQTL filtration.** Outlier individuals can have a large impact on detecting an eQTL as significant or not (S5 Fig) [4]. Therefore, it is reasonable to query linear model assumptions and detect influential outlier individuals that heavily influence the significance of the model especially when making associations to disease phenotypes. We classified eQTLs as high confidence or low confidence. Low confidence eQTLs either violated linear modeling assumptions or had results largely dependent on one influential outlier individual (S5 Fig). It is possible that low confidence eQTLs are still biologically relevant, however they will likely be difficult to reproduce in follow up studies or might require independent validation. Yu et al. (2016) highlighted the importance of increasing confidence for disease associated eQTLs, and classifies eQTLs as 'high-confidence' if the eQTL is present across multiple studies [5]. We are not capable of comparing across studies as we are presenting the first genome wide eQTL study in horses. Therefore, to avoid presenting spurious results we calculated Cooks Distance and leverage to detect outlier individuals that have large effects on the slope of the fitted line, and tested linear model assumptions for all significant eQTLs. These basic calculations could increase reproducibility of results when no additional eQTL results are available for comparison.

***Trans* Regulatory hotspot filtration.** Prior to filtering out low confidence eQTLs, we observed three additional *trans* regulatory hotspots on chromosomes 24, 28, 29 in the HDE9 treatment (S11 Fig). However, after filtering out low confidence eQTLs the *trans* regulatory hotspots were no longer observed on chromosome 28 and 29, and the number of eQTLs supporting the *trans* regulatory hotspot on chromosome 24 was reduced in the HDE9 treatment (Fig 4, S11 Fig). This indicates that filtering for outliers and linear modeling assumptions are important to avoid spurious results.

**Batch effects**

**Confounding variation.** Confounding variation in the gene expression matrix reduces power to detect correlations between genotype and gene expression variation in eQTL analyses [6]. Some variables known to introduce confounding variation are age, sex, or sequencing batch. These factors can influence the number of expressed RNA molecules independently of genetic regulators of gene expression. Incorporating these known confounders into the linear model will account for a portion of the confounding variation. However, it is becoming increasingly apparent that unknown factors contribute to broad patterns of confounding variation, i.e. batch effects, in the gene expression profile. If unaccounted for, these batch effects reduce the sensitivity of analyses. Programs such as Probabilistic Estimation of Expression Residuals (PEER) searches for variables that have an effect size in all genes and therefore have a broad effect on gene expression patterns across all quantified genes [6, 7]. Batch effects can be introduced during sample processing in the laboratory, or when a subgroup of samples are processed independently or differently than other samples. Batch effects are accounted for by either including covariates (factors or surrogate variables) in downstream analyses, or regressing out the factors from the gene expression matrix [7].

**eQTL Software**

Two key types of data are required to detect eQTLs, DNA sequence variants and quantification of RNA expression. These data are collected genome wide with next generation sequencing technologies and/or microarrays. Programs such as Matrix eQTL discover eQTLs by modeling relationships between DNA variants and the number of aligned RNA sequences to genomic features [8, 9]. These sequencing and analytical tools have helped streamline genome wide eQTL identification.

**Matrix eQTL.** Matrix eQTL is a fast and efficient *cis* and *trans* eQTL discovery tool that models additive changes in gene expression by genotype [8]. Matrix eQTL can account for small levels of population structure, however it can not accept dependent data from the same individuals across multiple treatments or tissues. To increase computational efficiency, Matrix eQTL calculates only essential information to determine significance of an eQTL [10, 11]. However, Matrix eQTL does not calculate the residuals of a linear model. Residuals quantify unexplained variation in a linear model, and are used to detect outliers as well as test if linear model assumptions are violated. Gene expression outliers for samples or genes can have a large impact on the reproducibility of results of an eQTL analysis as mentioned in the eQTL 'gold standards' [4]. In addition, eQTLs are to some degree cell type, treatment, and population specific, and eQTLs can be difficult to reproduce in replication experiments due to plastic RNA expression profiles [5, 12]. To address these issues, researchers now classify eQTLs as 'high confidence' eQTLs if the eQTL was identified in more than one study, while others have expanded eQTL detection methodologies to jointly model multiple cell types in one analysis [5, 9, 13, 14]. Therefore, detecting influential outliers is important for eQTL analyses to produce robust results, replication experiments are important to increase confidence in eQTL signals, and joint modeling of multiple cell types, treatments of cells, or populations help to identify shared eQTLs across multiple categories.

**eQTLBMA.** eQTLBMA jointly models samples from multiple tissue types, different environmental conditions, or different cell treatments to detect eQTLs fitting an additive linear model [9, 12]. eQTLBMA was initially described by jointly modeling different cell types. However, here we jointly modeled PBMCs that were treated *in vitro* with four different treatments (therefore we will refer to joint modeling of different treatments of cells for this paper rather than joint modeling of different cell types) [9]. Joint modeling in eQTLBMA increases power of eQTL detection, while Matrix eQTL must analyze each treatment separately due to the assumption of sample independence in the linear model. Different sample sizes between treatments results in a loss of power in treatments with fewer samples when analyzed separately. eQTLBMA's hierarchical model also learns from the data itself to identify patterns of sharing across treatments [9, 13]. eQTLBMA leverages shared information across treatments to aid in eQTL detection across treatments, while single treatment analyses are biased against sharing of eQTLs across treatments. Differences in power and methods has caused the proportion of eQTLs shared across treatments to vary widely between different eQTL analytical methods [12]. Therefore, the method of analysis used for eQTL detection impacts the proportion of shared eQTLs reported. This complicates interpretation of eQTL results making it difficult to differentiate artifactual results introduced by methodology from true results with a biological origin.

**Joint modeling with eQTLBMA.** We discovered 3990 significant linear *cis* eQTLs by jointly modeling all four treatments in eQTLBMA (S18 Table). 1071 (94.2%) of the genes shared across all four treatments in Matrix eQTL are also identified in eQTLBMA (S6B Fig). 3758 (94.2%) of genes present in significant eQTLs in eQTLBMA were also identified to be regulated by a genetic variant in Matrix eQTL. eQTLBMA reported a very high percentage of eQTLs to be shared across all treatments at ~99%, however this signal may or may not be artificial (Personal communication google group: https://groups.google.com/forum/#!topic/eqtlbma-users/ooueJnKUZ3A (S6A Fig).

**Supplemental Bibliography**

1. Love MI, Anders S, Huber W. Differential analysis of count data - the DESeq2 package. 2014. doi:110.1186/s13059-014-0550-8.

2. Koh W, Sheng CT, Tan B, Lee QY, Kuznetsov V, Kiang LS, et al. Analysis of deep sequencing microRNA expression profile from human embryonic stem cells derived mesenchymal stem cells reveals possible role of let-7 microRNA family in downstream targeting of Hepatic Nuclear Factor 4 Alpha. BMC Genomics. 2010;11 Suppl 1:S6. doi:10.1186/1471-2164-11-S1-S6.

3. Farrell D, Shaughnessy RG, Britton L, MacHugh DE, Markey B, Gordon S V. The identification of circulating MiRNA in bovine serum and their potential as novel biomarkers of early mycobacterium avium subsp paratuberculosis infection. PLoS One. 2015;10:1–22. doi:10.1371/journal.pone.0134310.

4. Ellis SE, Gupta S, Ashar FN, Bader JS, West AB, Arking DE. RNA-Seq optimization with eQTL gold standards. BMC Genomics. 2013;14:892. doi:10.1186/1471-2164-14-892.

5. Yu C-H, Pal LR, Moult J. Consensus Genome-Wide Expression Quantitative Trait Loci and Their Relationship with Human Complex Trait Disease. Omi A J Integr Biol. 2016;20:400–14. doi:10.1089/omi.2016.0063.

6. Stegle O, Parts L, Durbin R, Winn J. A bayesian framework to account for complex non-genetic factors in gene expression levels greatly increases power in eQTL studies. PLoS Comput Biol. 2010;6:1–11.

7. Stegle O, Parts L, Piipari M, Winn J, Durbin R. Using probabilistic estimation of expression residuals (PEER) to obtain increased power and interpretability of gene expression analyses. Nat Protoc. 2012;7:500–7. doi:10.1038/nprot.2011.457.

8. Shabalin AA. Matrix eQTL: Ultra fast eQTL analysis via large matrix operations. Bioinformatics. 2012;28:1353–8.

9. Flutre T, Wen X, Pritchard J, Stephens M. A Statistical Framework for Joint eQTL Analysis in Multiple Tissues. PLoS Genet. 2013;9.

10. Benjamin, Y., Hochberg Y. Controlling the False Discovery Rate : A Practical and Powerful Approach to Multiple Testing Author ( s ): Yoav Benjamini and Yosef Hochberg Source : Journal of the Royal Statistical Society . Series B ( Methodological ), Vol . 57 , No . 1 Published by : J Roy Stat Soc. 1995;57:289–300.

11. Xia K, Shabalin AA, Huang S, Madar V, Zhou YH, Wang W, et al. SeeQTL: A searchable database for human eQTLs. Bioinformatics. 2012;28:451–2.

12. Peters JE, Lyons PA, Lee JC, Richard AC, Fortune MD, Newcombe PJ, et al. Insight into Genotype-Phenotype Associations through eQTL Mapping in Multiple Cell Types in Health and Immune-Mediated Disease. PLoS Genet. 2016;12. doi:10.1371/journal.pgen.1005908.

13. Urbut SM, Wang G, Stephens M. Flexible statistical methods for estimating and testing effects in genomic studies with multiple conditions. 2016.

14. Casale FP, Horta D, Rakitsch B, Stegle O, Abecasis G, Salem R. Joint genetic analysis using variant sets reveals polygenic gene-context interactions. PLOS Genet. 2017;13:e1006693. doi:10.1371/journal.pgen.1006693.
