# Supplementary figures and images for: eQTL discovery and their association with severe equine asthma in European Warmblood horses
Source: BMC Genomics. 2018 Aug 2;19:581. doi: 10.1186/s12864-018-4938-9 (PMC6090848; doi:10.1186/s12864-018-4938-9)

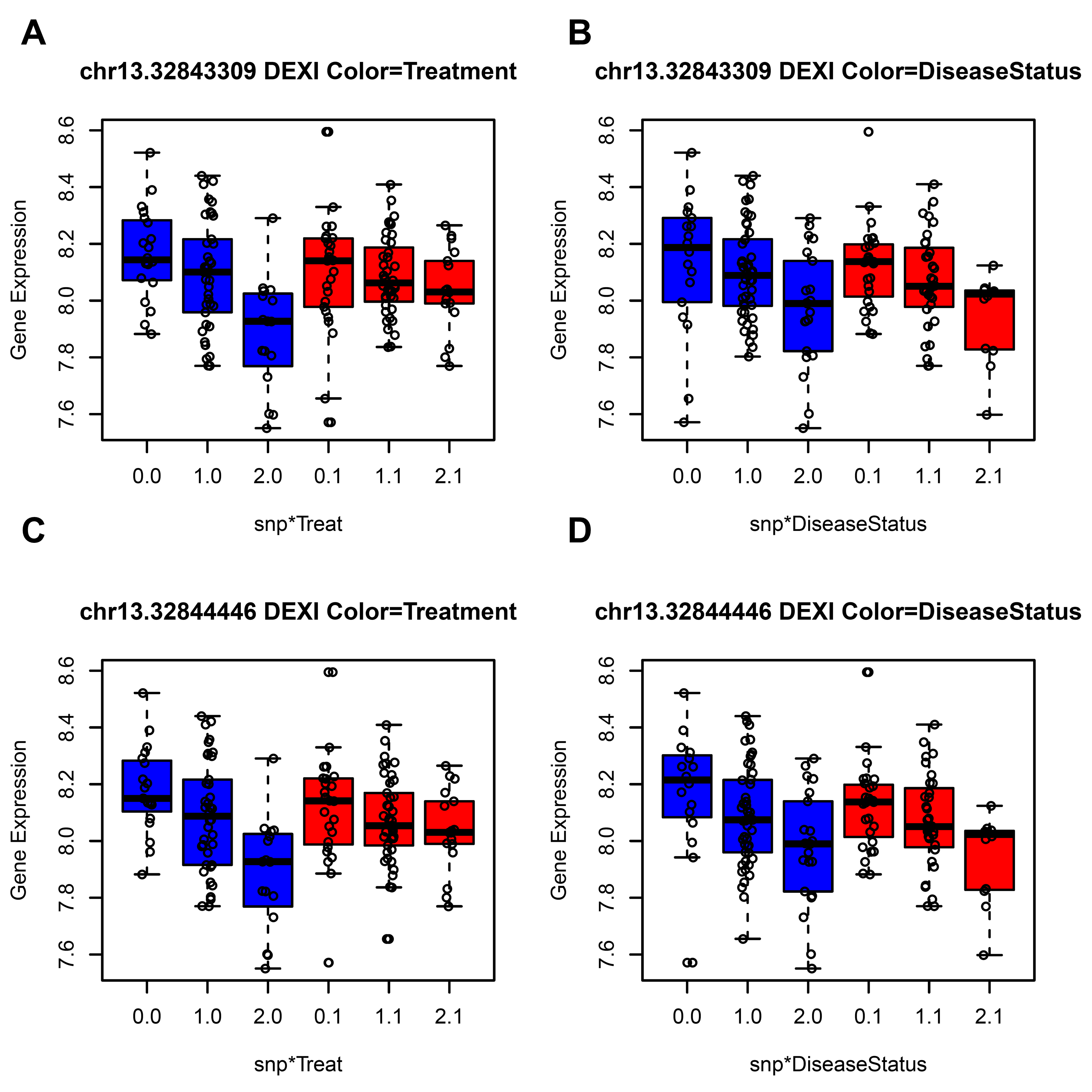

Supplement: Supplementary file 1 — Figure S1. Minimum D-statistics determine mean read count cutoffs. Figure S2. PCA plots of normalized variance stabilized RNAseq counts after KS test filter. Figure S3. PCA plots of 1,056,195 SNP genotypes and colored by cohort. Figure S4. Matrix eQTL histograms and QQ-plots for all p-values for all cis and trans eQTL analyses using tag SNPs for the MCK1 treatment. Figure S5. Low confidence cis eQTLs. Figure S6. Joint modeling with eQTLBMA with possible overestimation of shared eQTLs across all PBMC treatments. Figure S7. Distance between eSNPs with the lowest FDR values per gene is small. Figure S8.. Enrichment of SNPs in trans regulatory hotspots genome wide. Figure S9. GWAS for RAO. Figure S10. Loss of DEXI gene expression regulation in HDE. Figure S11. Cis trans eQTL plot for all eQTLs for treatment HDE9. Table S1. High confidence additive linear cis eQTLs from the MCK treatment. Table S2. Low confidence additive linear cis eQTLs from the MCK treatment. Table S3. High confidence additive linear trans eQTLs from the MCK treatment. Table S4. Low confidence additive linear trans eQTLs from the MCK treatment. Table S5. High confidence additive linear cis eQTLs from the LPS treatment. Table S6. Low confidence additive linear cis eQTLs from the LPS treatment. Table S7. High confidence additive linear trans eQTLs from the LPS treatment. Table S8. Low confidence additive linear trans eQTLs from the LPS treatment. Table S9. High confidence additive linear cis eQTLs from the RCA treatment. Table S10. Low confidence additive linear cis eQTLs from the RCA treatment. Table S11. High confidence additive linear trans eQTLs from the RCA treatment. The eQTLs reported are limited to one eQTL per gene, representing the eSNP with the lowest FDR value for each gene. Table S12. Low confidence additive linear trans eQTLs from the RCA treatment. Table S13. High confidence additive linear cis eQTLs from the HDE treatment. Table S14. Low confidence additive linear cis eQTLs from the HDE [file 12864_2018_4938_MOESM1_ESM.zip › S10_Fig.tif]

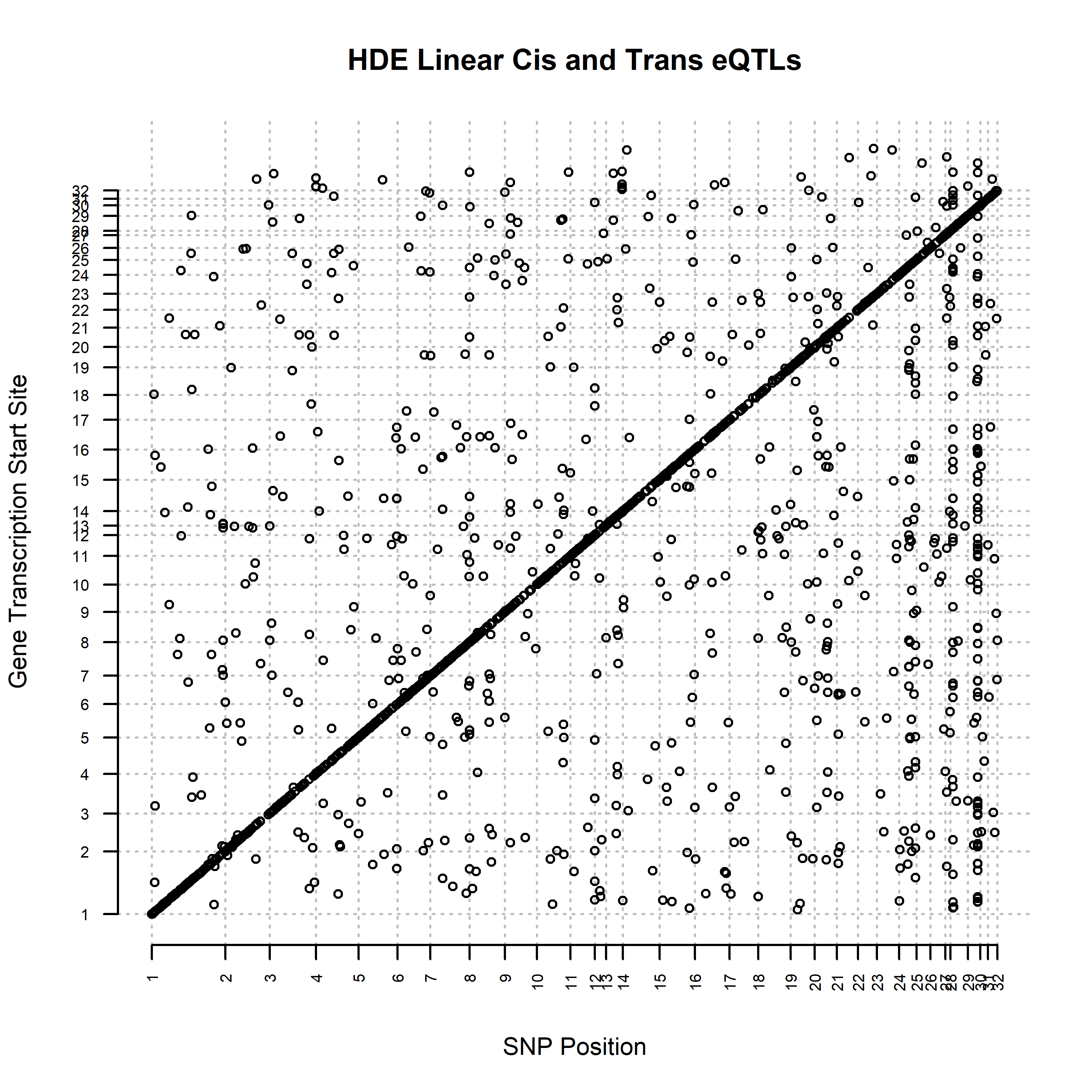

Supplement: Supplementary file 1 — Figure S1. Minimum D-statistics determine mean read count cutoffs. Figure S2. PCA plots of normalized variance stabilized RNAseq counts after KS test filter. Figure S3. PCA plots of 1,056,195 SNP genotypes and colored by cohort. Figure S4. Matrix eQTL histograms and QQ-plots for all p-values for all cis and trans eQTL analyses using tag SNPs for the MCK1 treatment. Figure S5. Low confidence cis eQTLs. Figure S6. Joint modeling with eQTLBMA with possible overestimation of shared eQTLs across all PBMC treatments. Figure S7. Distance between eSNPs with the lowest FDR values per gene is small. Figure S8.. Enrichment of SNPs in trans regulatory hotspots genome wide. Figure S9. GWAS for RAO. Figure S10. Loss of DEXI gene expression regulation in HDE. Figure S11. Cis trans eQTL plot for all eQTLs for treatment HDE9. Table S1. High confidence additive linear cis eQTLs from the MCK treatment. Table S2. Low confidence additive linear cis eQTLs from the MCK treatment. Table S3. High confidence additive linear trans eQTLs from the MCK treatment. Table S4. Low confidence additive linear trans eQTLs from the MCK treatment. Table S5. High confidence additive linear cis eQTLs from the LPS treatment. Table S6. Low confidence additive linear cis eQTLs from the LPS treatment. Table S7. High confidence additive linear trans eQTLs from the LPS treatment. Table S8. Low confidence additive linear trans eQTLs from the LPS treatment. Table S9. High confidence additive linear cis eQTLs from the RCA treatment. Table S10. Low confidence additive linear cis eQTLs from the RCA treatment. Table S11. High confidence additive linear trans eQTLs from the RCA treatment. The eQTLs reported are limited to one eQTL per gene, representing the eSNP with the lowest FDR value for each gene. Table S12. Low confidence additive linear trans eQTLs from the RCA treatment. Table S13. High confidence additive linear cis eQTLs from the HDE treatment. Table S14. Low confidence additive linear cis eQTLs from the HDE [file 12864_2018_4938_MOESM1_ESM.zip › S11_Fig.tif]

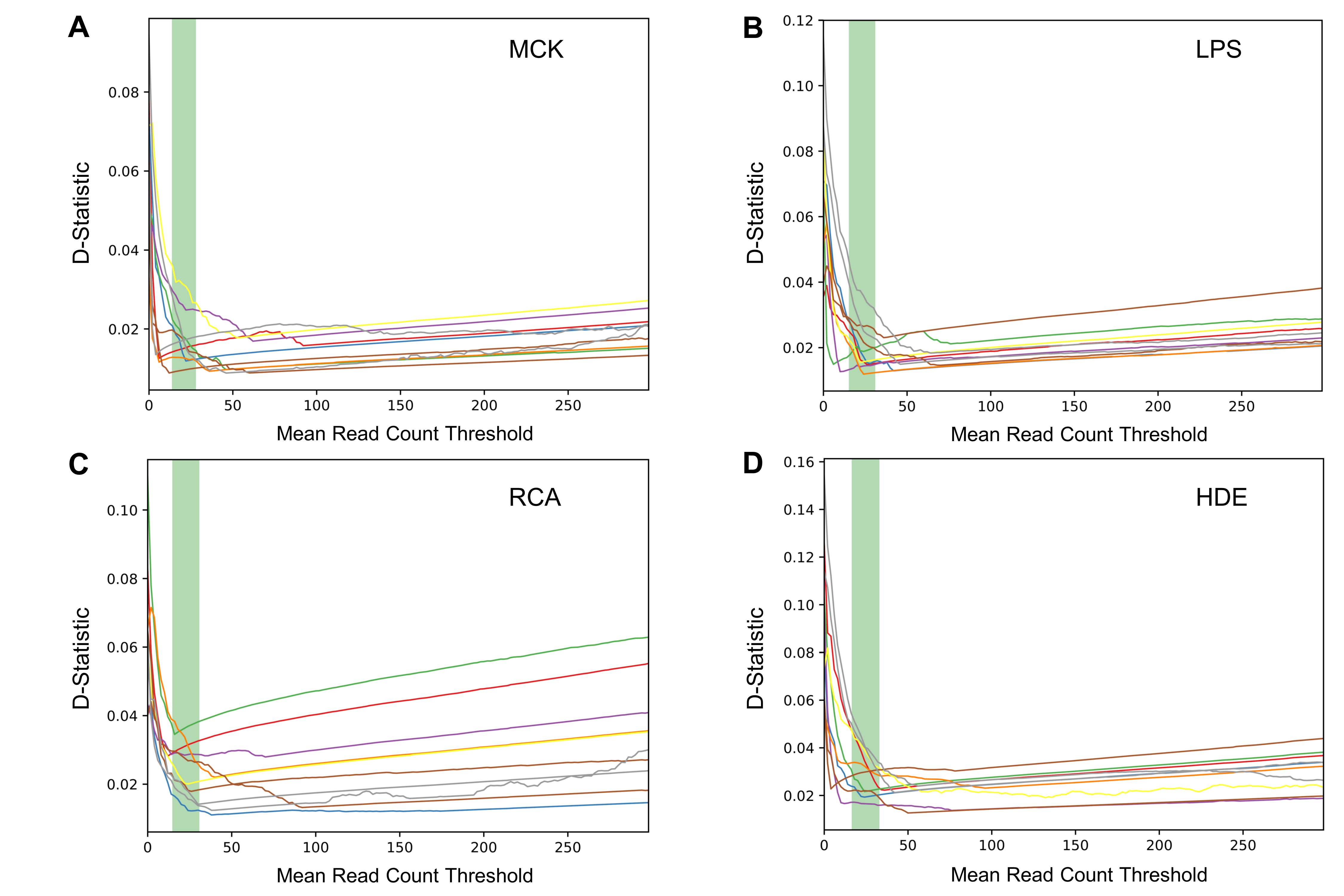

Supplement: Supplementary file 1 — Figure S1. Minimum D-statistics determine mean read count cutoffs. Figure S2. PCA plots of normalized variance stabilized RNAseq counts after KS test filter. Figure S3. PCA plots of 1,056,195 SNP genotypes and colored by cohort. Figure S4. Matrix eQTL histograms and QQ-plots for all p-values for all cis and trans eQTL analyses using tag SNPs for the MCK1 treatment. Figure S5. Low confidence cis eQTLs. Figure S6. Joint modeling with eQTLBMA with possible overestimation of shared eQTLs across all PBMC treatments. Figure S7. Distance between eSNPs with the lowest FDR values per gene is small. Figure S8.. Enrichment of SNPs in trans regulatory hotspots genome wide. Figure S9. GWAS for RAO. Figure S10. Loss of DEXI gene expression regulation in HDE. Figure S11. Cis trans eQTL plot for all eQTLs for treatment HDE9. Table S1. High confidence additive linear cis eQTLs from the MCK treatment. Table S2. Low confidence additive linear cis eQTLs from the MCK treatment. Table S3. High confidence additive linear trans eQTLs from the MCK treatment. Table S4. Low confidence additive linear trans eQTLs from the MCK treatment. Table S5. High confidence additive linear cis eQTLs from the LPS treatment. Table S6. Low confidence additive linear cis eQTLs from the LPS treatment. Table S7. High confidence additive linear trans eQTLs from the LPS treatment. Table S8. Low confidence additive linear trans eQTLs from the LPS treatment. Table S9. High confidence additive linear cis eQTLs from the RCA treatment. Table S10. Low confidence additive linear cis eQTLs from the RCA treatment. Table S11. High confidence additive linear trans eQTLs from the RCA treatment. The eQTLs reported are limited to one eQTL per gene, representing the eSNP with the lowest FDR value for each gene. Table S12. Low confidence additive linear trans eQTLs from the RCA treatment. Table S13. High confidence additive linear cis eQTLs from the HDE treatment. Table S14. Low confidence additive linear cis eQTLs from the HDE [file 12864_2018_4938_MOESM1_ESM.zip › S1_Fig.tif]

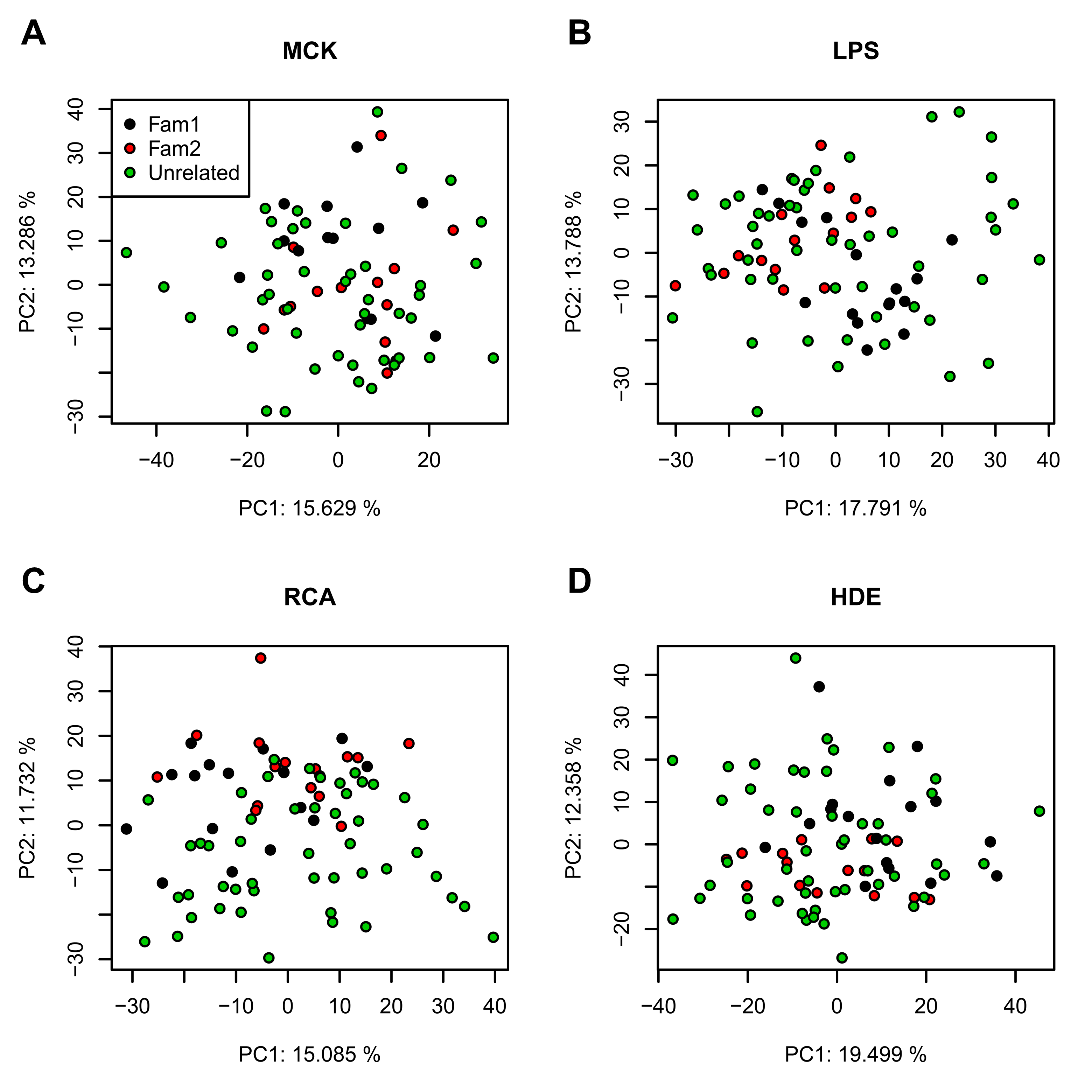

Supplement: Supplementary file 1 — Figure S1. Minimum D-statistics determine mean read count cutoffs. Figure S2. PCA plots of normalized variance stabilized RNAseq counts after KS test filter. Figure S3. PCA plots of 1,056,195 SNP genotypes and colored by cohort. Figure S4. Matrix eQTL histograms and QQ-plots for all p-values for all cis and trans eQTL analyses using tag SNPs for the MCK1 treatment. Figure S5. Low confidence cis eQTLs. Figure S6. Joint modeling with eQTLBMA with possible overestimation of shared eQTLs across all PBMC treatments. Figure S7. Distance between eSNPs with the lowest FDR values per gene is small. Figure S8.. Enrichment of SNPs in trans regulatory hotspots genome wide. Figure S9. GWAS for RAO. Figure S10. Loss of DEXI gene expression regulation in HDE. Figure S11. Cis trans eQTL plot for all eQTLs for treatment HDE9. Table S1. High confidence additive linear cis eQTLs from the MCK treatment. Table S2. Low confidence additive linear cis eQTLs from the MCK treatment. Table S3. High confidence additive linear trans eQTLs from the MCK treatment. Table S4. Low confidence additive linear trans eQTLs from the MCK treatment. Table S5. High confidence additive linear cis eQTLs from the LPS treatment. Table S6. Low confidence additive linear cis eQTLs from the LPS treatment. Table S7. High confidence additive linear trans eQTLs from the LPS treatment. Table S8. Low confidence additive linear trans eQTLs from the LPS treatment. Table S9. High confidence additive linear cis eQTLs from the RCA treatment. Table S10. Low confidence additive linear cis eQTLs from the RCA treatment. Table S11. High confidence additive linear trans eQTLs from the RCA treatment. The eQTLs reported are limited to one eQTL per gene, representing the eSNP with the lowest FDR value for each gene. Table S12. Low confidence additive linear trans eQTLs from the RCA treatment. Table S13. High confidence additive linear cis eQTLs from the HDE treatment. Table S14. Low confidence additive linear cis eQTLs from the HDE [file 12864_2018_4938_MOESM1_ESM.zip › S2_Fig.tif]

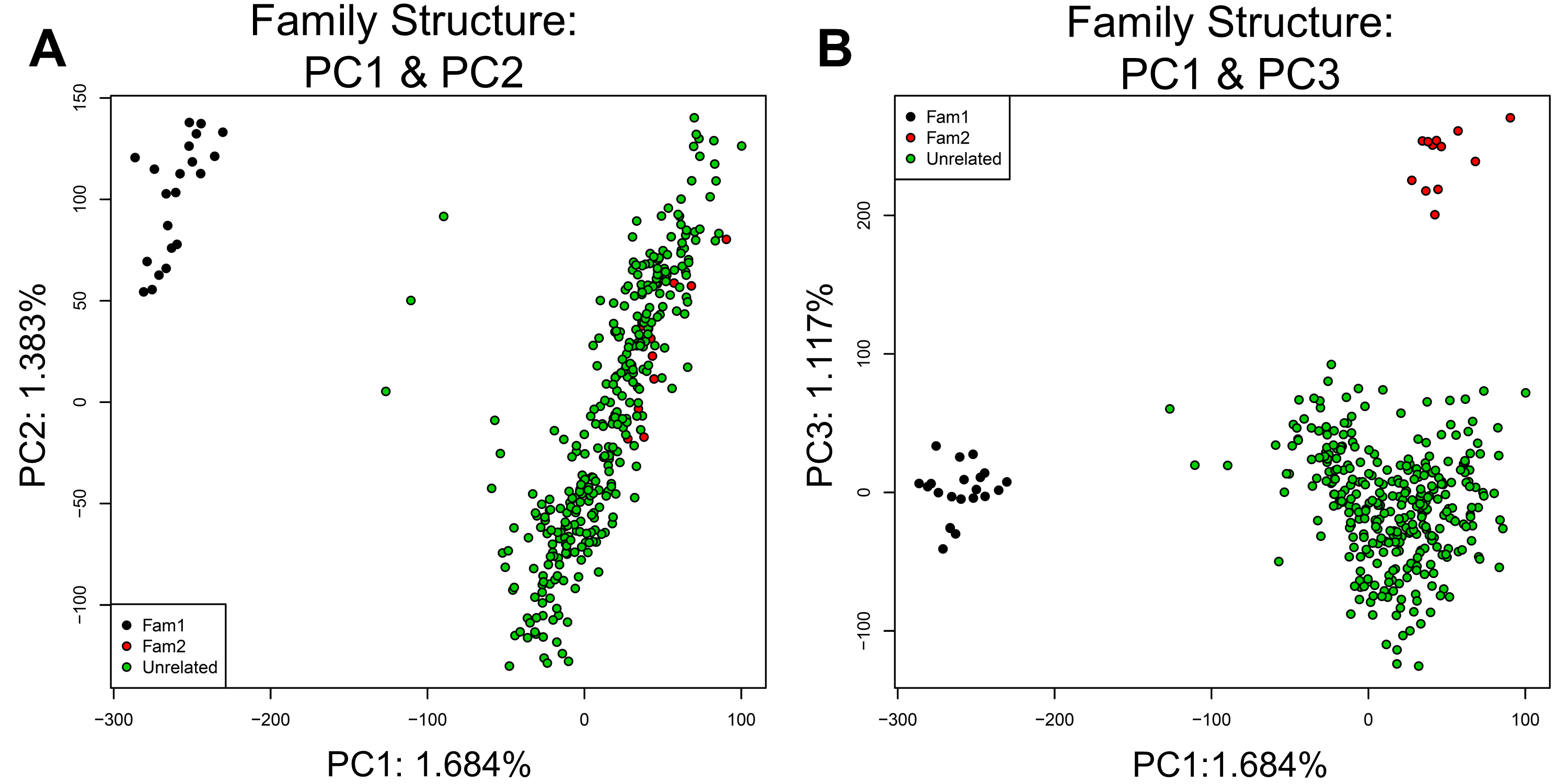

Supplement: Supplementary file 1 — Figure S1. Minimum D-statistics determine mean read count cutoffs. Figure S2. PCA plots of normalized variance stabilized RNAseq counts after KS test filter. Figure S3. PCA plots of 1,056,195 SNP genotypes and colored by cohort. Figure S4. Matrix eQTL histograms and QQ-plots for all p-values for all cis and trans eQTL analyses using tag SNPs for the MCK1 treatment. Figure S5. Low confidence cis eQTLs. Figure S6. Joint modeling with eQTLBMA with possible overestimation of shared eQTLs across all PBMC treatments. Figure S7. Distance between eSNPs with the lowest FDR values per gene is small. Figure S8.. Enrichment of SNPs in trans regulatory hotspots genome wide. Figure S9. GWAS for RAO. Figure S10. Loss of DEXI gene expression regulation in HDE. Figure S11. Cis trans eQTL plot for all eQTLs for treatment HDE9. Table S1. High confidence additive linear cis eQTLs from the MCK treatment. Table S2. Low confidence additive linear cis eQTLs from the MCK treatment. Table S3. High confidence additive linear trans eQTLs from the MCK treatment. Table S4. Low confidence additive linear trans eQTLs from the MCK treatment. Table S5. High confidence additive linear cis eQTLs from the LPS treatment. Table S6. Low confidence additive linear cis eQTLs from the LPS treatment. Table S7. High confidence additive linear trans eQTLs from the LPS treatment. Table S8. Low confidence additive linear trans eQTLs from the LPS treatment. Table S9. High confidence additive linear cis eQTLs from the RCA treatment. Table S10. Low confidence additive linear cis eQTLs from the RCA treatment. Table S11. High confidence additive linear trans eQTLs from the RCA treatment. The eQTLs reported are limited to one eQTL per gene, representing the eSNP with the lowest FDR value for each gene. Table S12. Low confidence additive linear trans eQTLs from the RCA treatment. Table S13. High confidence additive linear cis eQTLs from the HDE treatment. Table S14. Low confidence additive linear cis eQTLs from the HDE [file 12864_2018_4938_MOESM1_ESM.zip › S3_Fig.tif]

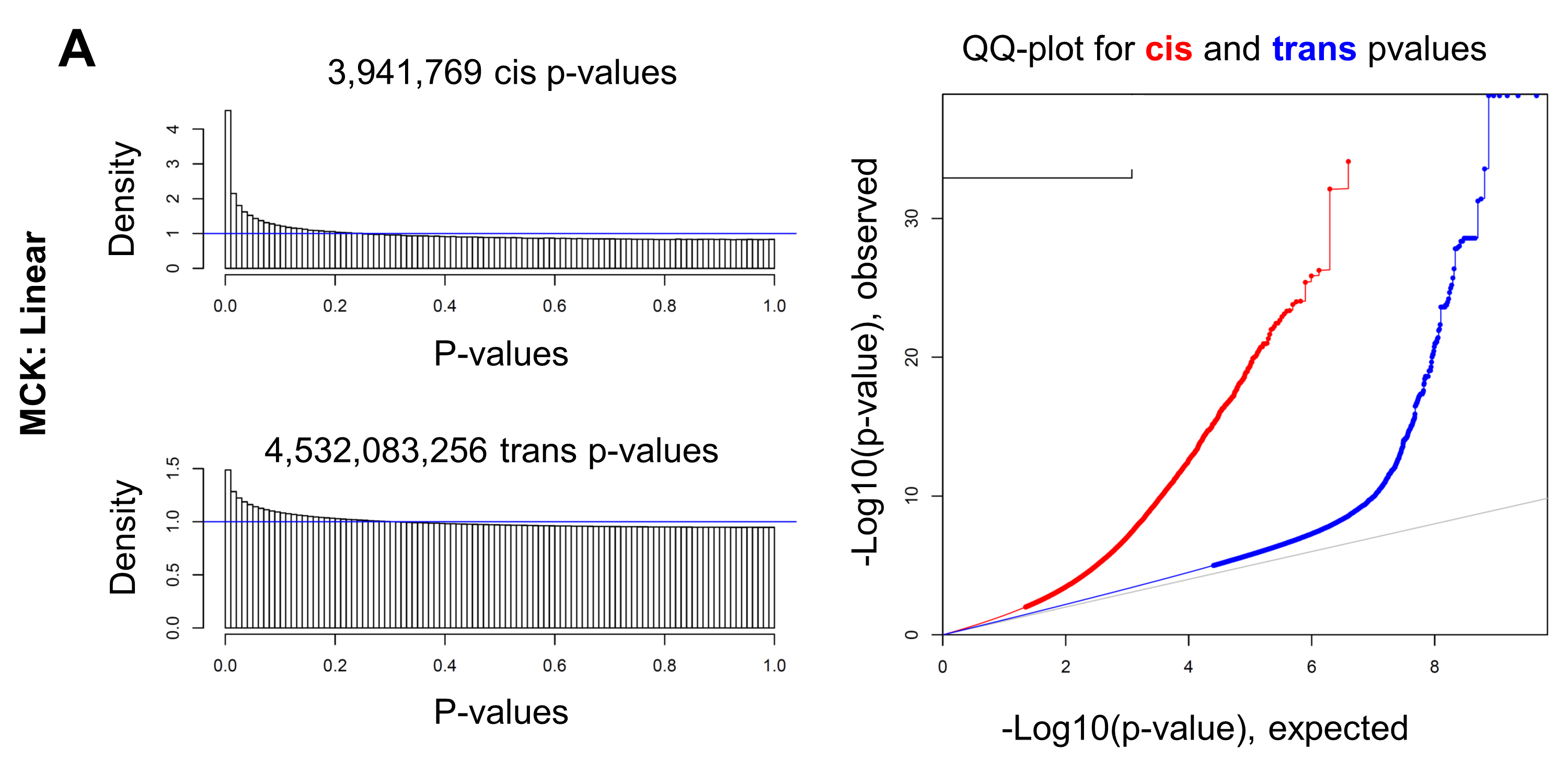

Supplement: Supplementary file 1 — Figure S1. Minimum D-statistics determine mean read count cutoffs. Figure S2. PCA plots of normalized variance stabilized RNAseq counts after KS test filter. Figure S3. PCA plots of 1,056,195 SNP genotypes and colored by cohort. Figure S4. Matrix eQTL histograms and QQ-plots for all p-values for all cis and trans eQTL analyses using tag SNPs for the MCK1 treatment. Figure S5. Low confidence cis eQTLs. Figure S6. Joint modeling with eQTLBMA with possible overestimation of shared eQTLs across all PBMC treatments. Figure S7. Distance between eSNPs with the lowest FDR values per gene is small. Figure S8.. Enrichment of SNPs in trans regulatory hotspots genome wide. Figure S9. GWAS for RAO. Figure S10. Loss of DEXI gene expression regulation in HDE. Figure S11. Cis trans eQTL plot for all eQTLs for treatment HDE9. Table S1. High confidence additive linear cis eQTLs from the MCK treatment. Table S2. Low confidence additive linear cis eQTLs from the MCK treatment. Table S3. High confidence additive linear trans eQTLs from the MCK treatment. Table S4. Low confidence additive linear trans eQTLs from the MCK treatment. Table S5. High confidence additive linear cis eQTLs from the LPS treatment. Table S6. Low confidence additive linear cis eQTLs from the LPS treatment. Table S7. High confidence additive linear trans eQTLs from the LPS treatment. Table S8. Low confidence additive linear trans eQTLs from the LPS treatment. Table S9. High confidence additive linear cis eQTLs from the RCA treatment. Table S10. Low confidence additive linear cis eQTLs from the RCA treatment. Table S11. High confidence additive linear trans eQTLs from the RCA treatment. The eQTLs reported are limited to one eQTL per gene, representing the eSNP with the lowest FDR value for each gene. Table S12. Low confidence additive linear trans eQTLs from the RCA treatment. Table S13. High confidence additive linear cis eQTLs from the HDE treatment. Table S14. Low confidence additive linear cis eQTLs from the HDE [file 12864_2018_4938_MOESM1_ESM.zip › S4_Fig.tif]

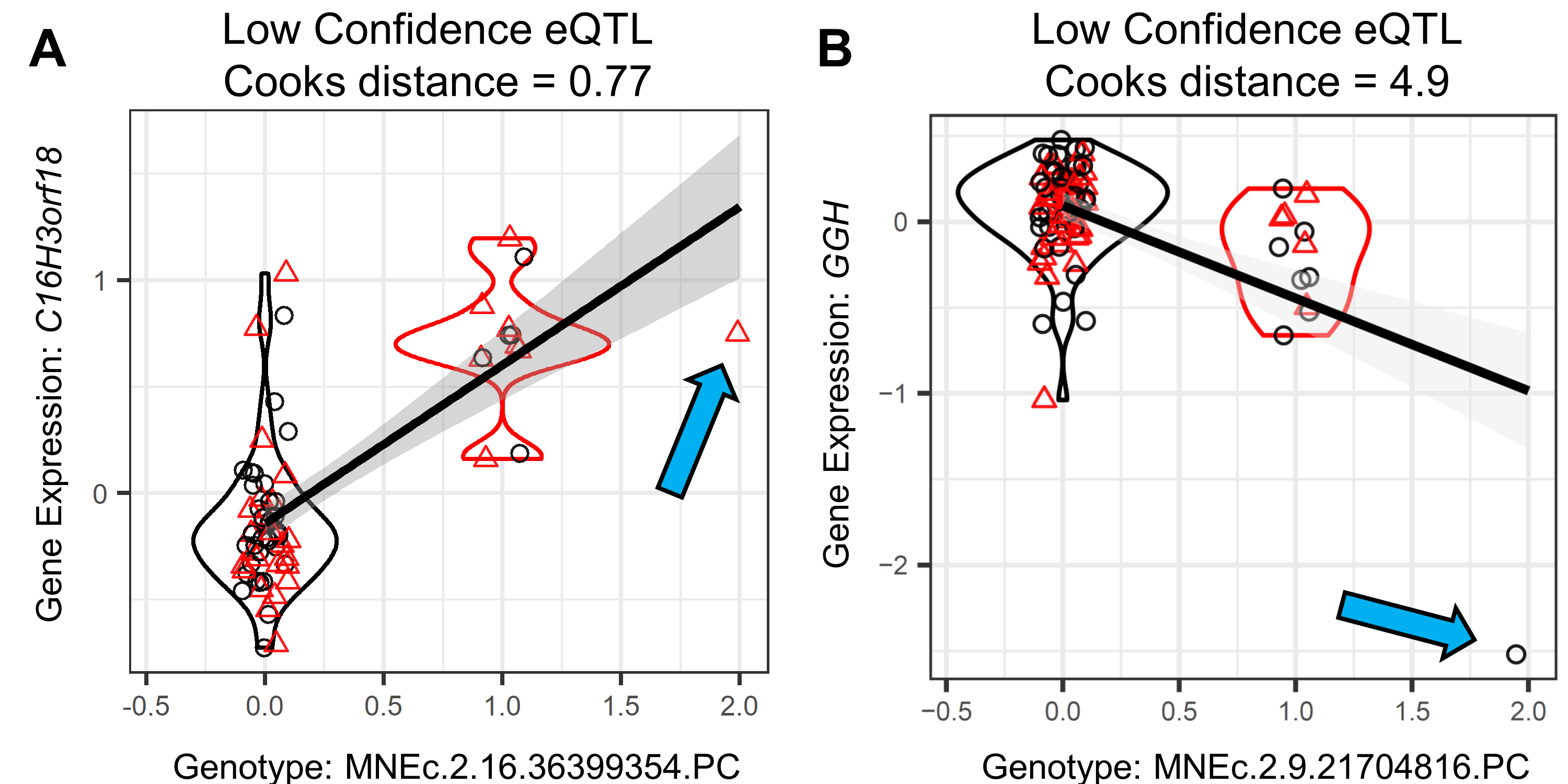

Supplement: Supplementary file 1 — Figure S1. Minimum D-statistics determine mean read count cutoffs. Figure S2. PCA plots of normalized variance stabilized RNAseq counts after KS test filter. Figure S3. PCA plots of 1,056,195 SNP genotypes and colored by cohort. Figure S4. Matrix eQTL histograms and QQ-plots for all p-values for all cis and trans eQTL analyses using tag SNPs for the MCK1 treatment. Figure S5. Low confidence cis eQTLs. Figure S6. Joint modeling with eQTLBMA with possible overestimation of shared eQTLs across all PBMC treatments. Figure S7. Distance between eSNPs with the lowest FDR values per gene is small. Figure S8.. Enrichment of SNPs in trans regulatory hotspots genome wide. Figure S9. GWAS for RAO. Figure S10. Loss of DEXI gene expression regulation in HDE. Figure S11. Cis trans eQTL plot for all eQTLs for treatment HDE9. Table S1. High confidence additive linear cis eQTLs from the MCK treatment. Table S2. Low confidence additive linear cis eQTLs from the MCK treatment. Table S3. High confidence additive linear trans eQTLs from the MCK treatment. Table S4. Low confidence additive linear trans eQTLs from the MCK treatment. Table S5. High confidence additive linear cis eQTLs from the LPS treatment. Table S6. Low confidence additive linear cis eQTLs from the LPS treatment. Table S7. High confidence additive linear trans eQTLs from the LPS treatment. Table S8. Low confidence additive linear trans eQTLs from the LPS treatment. Table S9. High confidence additive linear cis eQTLs from the RCA treatment. Table S10. Low confidence additive linear cis eQTLs from the RCA treatment. Table S11. High confidence additive linear trans eQTLs from the RCA treatment. The eQTLs reported are limited to one eQTL per gene, representing the eSNP with the lowest FDR value for each gene. Table S12. Low confidence additive linear trans eQTLs from the RCA treatment. Table S13. High confidence additive linear cis eQTLs from the HDE treatment. Table S14. Low confidence additive linear cis eQTLs from the HDE [file 12864_2018_4938_MOESM1_ESM.zip › S5_Fig.tif]

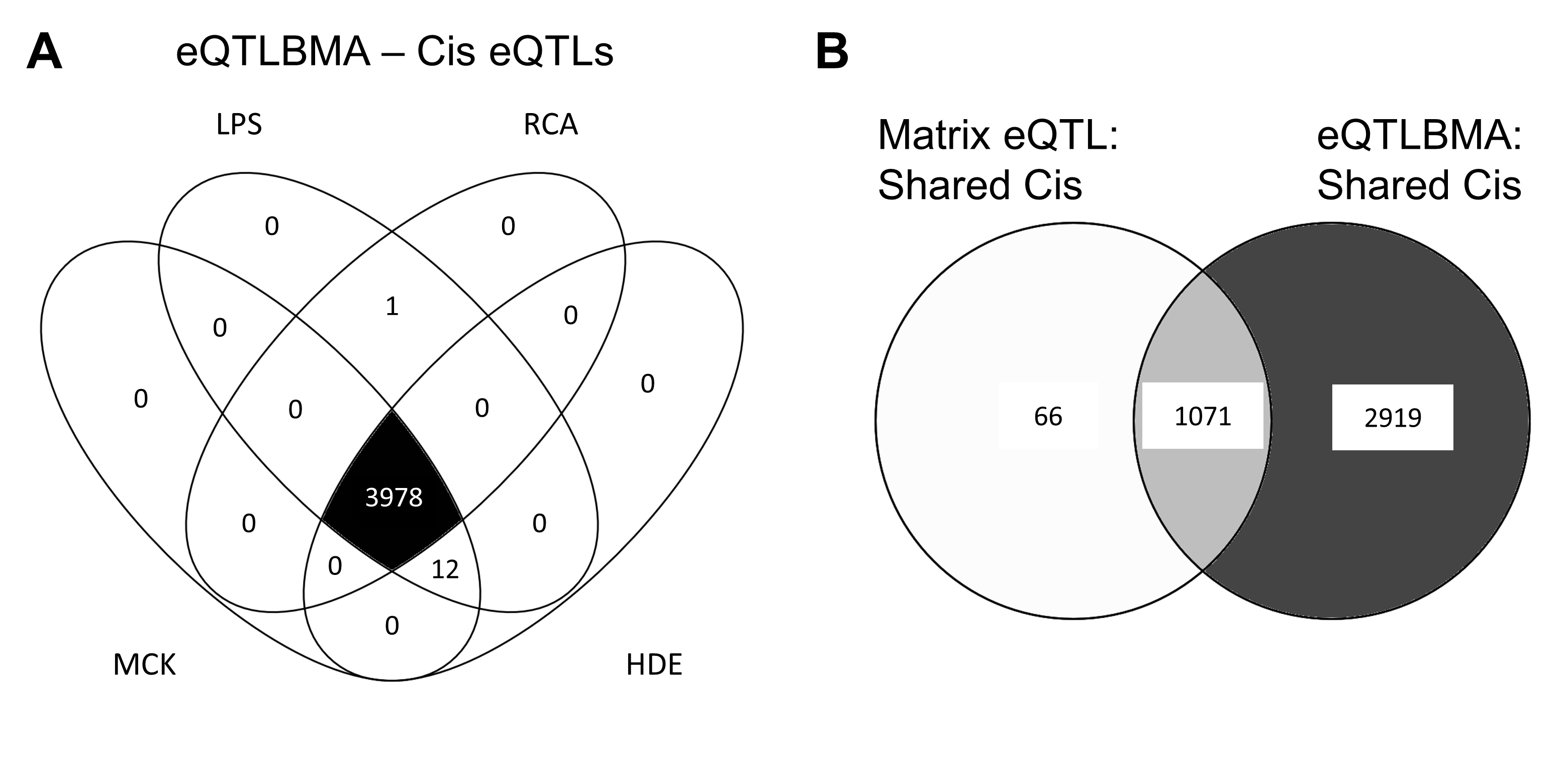

Supplement: Supplementary file 1 — Figure S1. Minimum D-statistics determine mean read count cutoffs. Figure S2. PCA plots of normalized variance stabilized RNAseq counts after KS test filter. Figure S3. PCA plots of 1,056,195 SNP genotypes and colored by cohort. Figure S4. Matrix eQTL histograms and QQ-plots for all p-values for all cis and trans eQTL analyses using tag SNPs for the MCK1 treatment. Figure S5. Low confidence cis eQTLs. Figure S6. Joint modeling with eQTLBMA with possible overestimation of shared eQTLs across all PBMC treatments. Figure S7. Distance between eSNPs with the lowest FDR values per gene is small. Figure S8.. Enrichment of SNPs in trans regulatory hotspots genome wide. Figure S9. GWAS for RAO. Figure S10. Loss of DEXI gene expression regulation in HDE. Figure S11. Cis trans eQTL plot for all eQTLs for treatment HDE9. Table S1. High confidence additive linear cis eQTLs from the MCK treatment. Table S2. Low confidence additive linear cis eQTLs from the MCK treatment. Table S3. High confidence additive linear trans eQTLs from the MCK treatment. Table S4. Low confidence additive linear trans eQTLs from the MCK treatment. Table S5. High confidence additive linear cis eQTLs from the LPS treatment. Table S6. Low confidence additive linear cis eQTLs from the LPS treatment. Table S7. High confidence additive linear trans eQTLs from the LPS treatment. Table S8. Low confidence additive linear trans eQTLs from the LPS treatment. Table S9. High confidence additive linear cis eQTLs from the RCA treatment. Table S10. Low confidence additive linear cis eQTLs from the RCA treatment. Table S11. High confidence additive linear trans eQTLs from the RCA treatment. The eQTLs reported are limited to one eQTL per gene, representing the eSNP with the lowest FDR value for each gene. Table S12. Low confidence additive linear trans eQTLs from the RCA treatment. Table S13. High confidence additive linear cis eQTLs from the HDE treatment. Table S14. Low confidence additive linear cis eQTLs from the HDE [file 12864_2018_4938_MOESM1_ESM.zip › S6_Fig.tif]

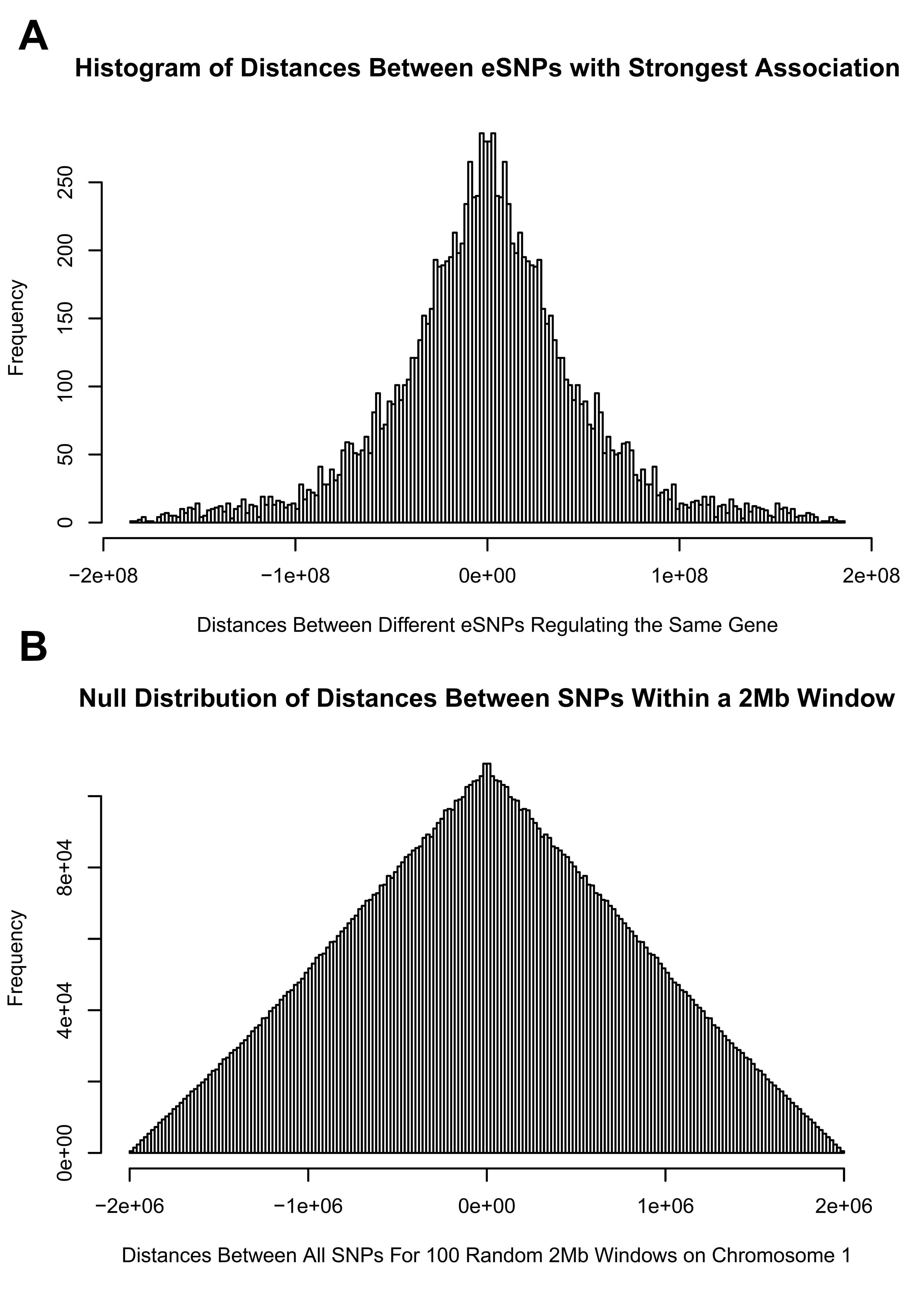

Supplement: Supplementary file 1 — Figure S1. Minimum D-statistics determine mean read count cutoffs. Figure S2. PCA plots of normalized variance stabilized RNAseq counts after KS test filter. Figure S3. PCA plots of 1,056,195 SNP genotypes and colored by cohort. Figure S4. Matrix eQTL histograms and QQ-plots for all p-values for all cis and trans eQTL analyses using tag SNPs for the MCK1 treatment. Figure S5. Low confidence cis eQTLs. Figure S6. Joint modeling with eQTLBMA with possible overestimation of shared eQTLs across all PBMC treatments. Figure S7. Distance between eSNPs with the lowest FDR values per gene is small. Figure S8.. Enrichment of SNPs in trans regulatory hotspots genome wide. Figure S9. GWAS for RAO. Figure S10. Loss of DEXI gene expression regulation in HDE. Figure S11. Cis trans eQTL plot for all eQTLs for treatment HDE9. Table S1. High confidence additive linear cis eQTLs from the MCK treatment. Table S2. Low confidence additive linear cis eQTLs from the MCK treatment. Table S3. High confidence additive linear trans eQTLs from the MCK treatment. Table S4. Low confidence additive linear trans eQTLs from the MCK treatment. Table S5. High confidence additive linear cis eQTLs from the LPS treatment. Table S6. Low confidence additive linear cis eQTLs from the LPS treatment. Table S7. High confidence additive linear trans eQTLs from the LPS treatment. Table S8. Low confidence additive linear trans eQTLs from the LPS treatment. Table S9. High confidence additive linear cis eQTLs from the RCA treatment. Table S10. Low confidence additive linear cis eQTLs from the RCA treatment. Table S11. High confidence additive linear trans eQTLs from the RCA treatment. The eQTLs reported are limited to one eQTL per gene, representing the eSNP with the lowest FDR value for each gene. Table S12. Low confidence additive linear trans eQTLs from the RCA treatment. Table S13. High confidence additive linear cis eQTLs from the HDE treatment. Table S14. Low confidence additive linear cis eQTLs from the HDE [file 12864_2018_4938_MOESM1_ESM.zip › S7_Fig.tif]

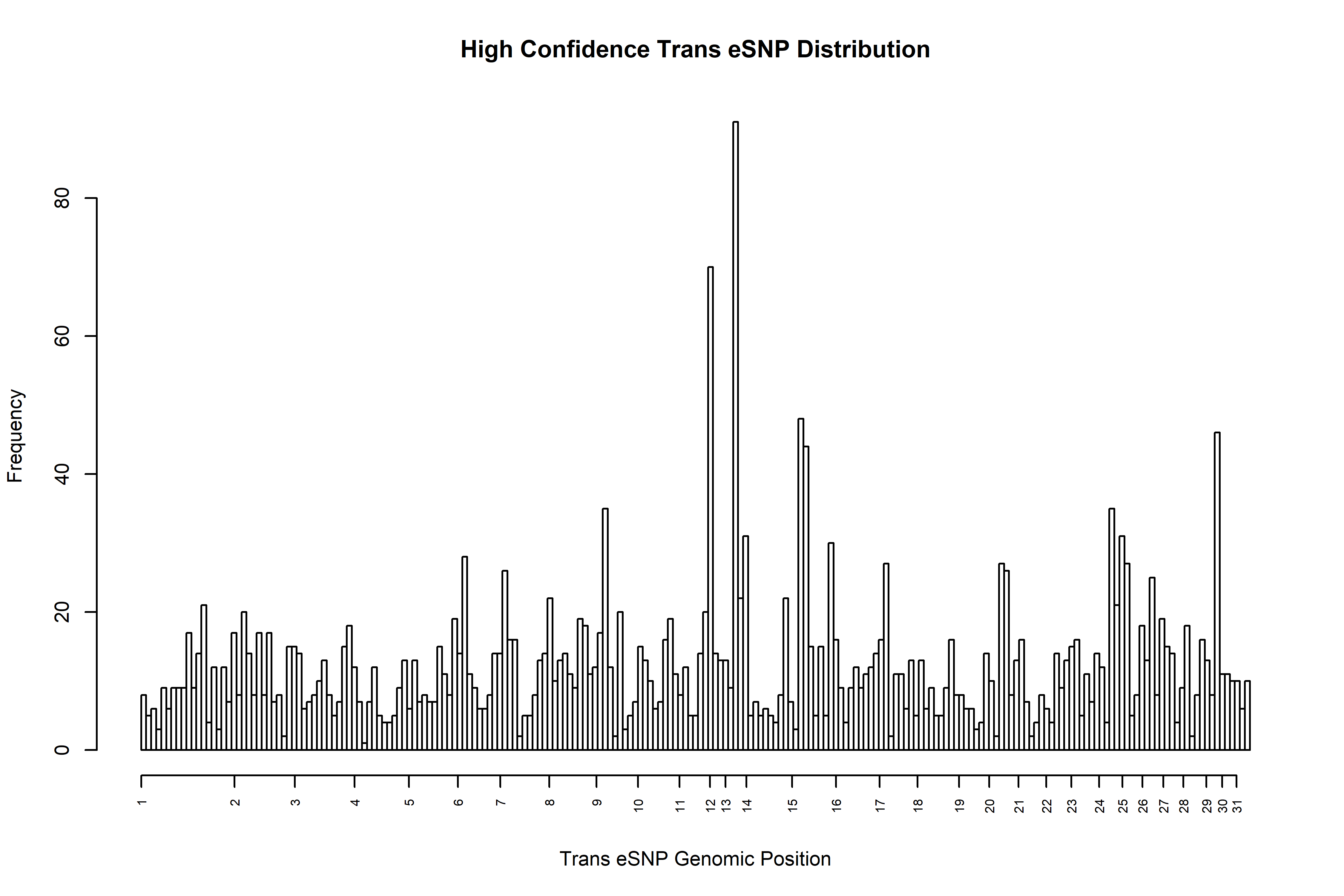

Supplement: Supplementary file 1 — Figure S1. Minimum D-statistics determine mean read count cutoffs. Figure S2. PCA plots of normalized variance stabilized RNAseq counts after KS test filter. Figure S3. PCA plots of 1,056,195 SNP genotypes and colored by cohort. Figure S4. Matrix eQTL histograms and QQ-plots for all p-values for all cis and trans eQTL analyses using tag SNPs for the MCK1 treatment. Figure S5. Low confidence cis eQTLs. Figure S6. Joint modeling with eQTLBMA with possible overestimation of shared eQTLs across all PBMC treatments. Figure S7. Distance between eSNPs with the lowest FDR values per gene is small. Figure S8.. Enrichment of SNPs in trans regulatory hotspots genome wide. Figure S9. GWAS for RAO. Figure S10. Loss of DEXI gene expression regulation in HDE. Figure S11. Cis trans eQTL plot for all eQTLs for treatment HDE9. Table S1. High confidence additive linear cis eQTLs from the MCK treatment. Table S2. Low confidence additive linear cis eQTLs from the MCK treatment. Table S3. High confidence additive linear trans eQTLs from the MCK treatment. Table S4. Low confidence additive linear trans eQTLs from the MCK treatment. Table S5. High confidence additive linear cis eQTLs from the LPS treatment. Table S6. Low confidence additive linear cis eQTLs from the LPS treatment. Table S7. High confidence additive linear trans eQTLs from the LPS treatment. Table S8. Low confidence additive linear trans eQTLs from the LPS treatment. Table S9. High confidence additive linear cis eQTLs from the RCA treatment. Table S10. Low confidence additive linear cis eQTLs from the RCA treatment. Table S11. High confidence additive linear trans eQTLs from the RCA treatment. The eQTLs reported are limited to one eQTL per gene, representing the eSNP with the lowest FDR value for each gene. Table S12. Low confidence additive linear trans eQTLs from the RCA treatment. Table S13. High confidence additive linear cis eQTLs from the HDE treatment. Table S14. Low confidence additive linear cis eQTLs from the HDE [file 12864_2018_4938_MOESM1_ESM.zip › S8_Fig.tif]

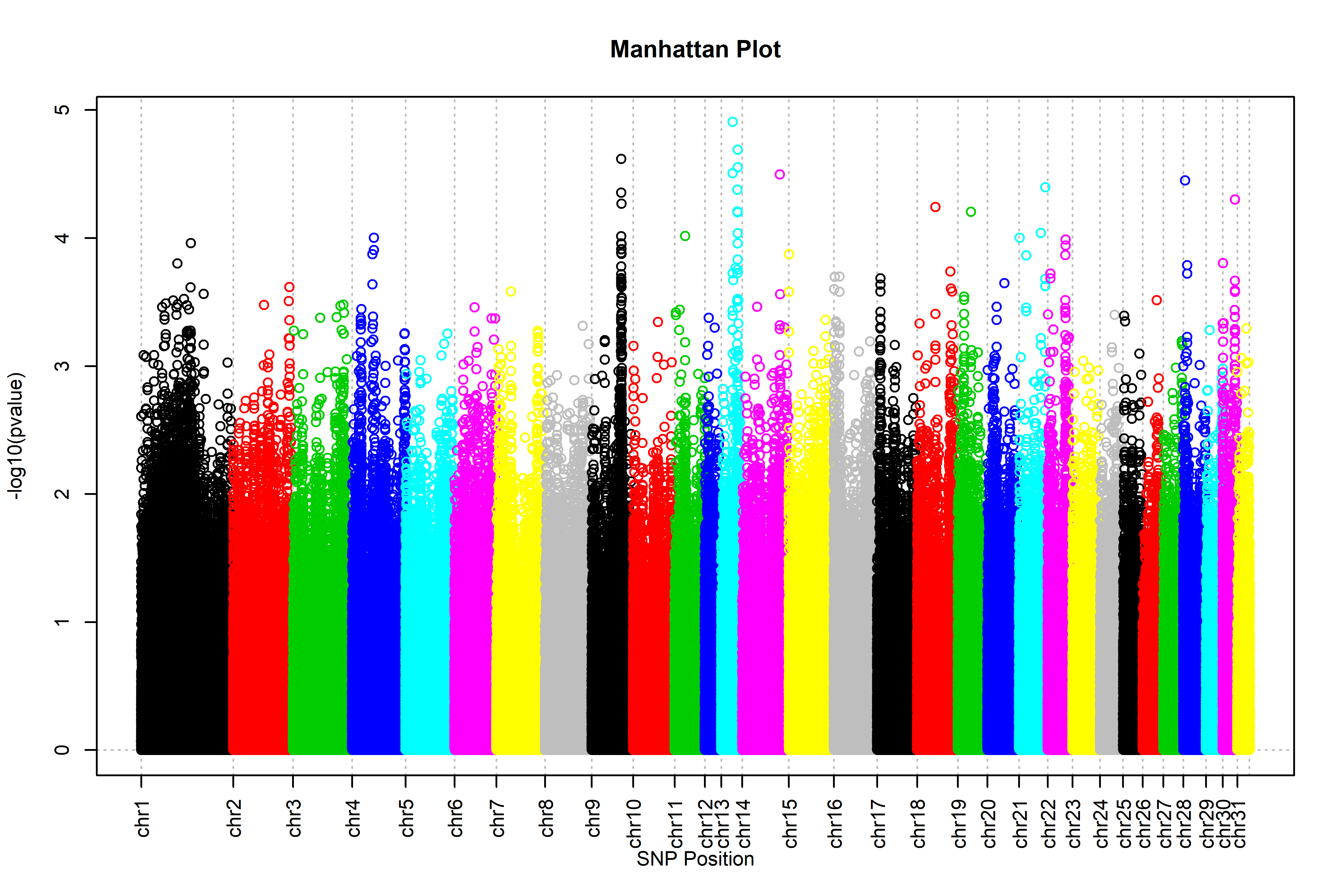

Supplement: Supplementary file 1 — Figure S1. Minimum D-statistics determine mean read count cutoffs. Figure S2. PCA plots of normalized variance stabilized RNAseq counts after KS test filter. Figure S3. PCA plots of 1,056,195 SNP genotypes and colored by cohort. Figure S4. Matrix eQTL histograms and QQ-plots for all p-values for all cis and trans eQTL analyses using tag SNPs for the MCK1 treatment. Figure S5. Low confidence cis eQTLs. Figure S6. Joint modeling with eQTLBMA with possible overestimation of shared eQTLs across all PBMC treatments. Figure S7. Distance between eSNPs with the lowest FDR values per gene is small. Figure S8.. Enrichment of SNPs in trans regulatory hotspots genome wide. Figure S9. GWAS for RAO. Figure S10. Loss of DEXI gene expression regulation in HDE. Figure S11. Cis trans eQTL plot for all eQTLs for treatment HDE9. Table S1. High confidence additive linear cis eQTLs from the MCK treatment. Table S2. Low confidence additive linear cis eQTLs from the MCK treatment. Table S3. High confidence additive linear trans eQTLs from the MCK treatment. Table S4. Low confidence additive linear trans eQTLs from the MCK treatment. Table S5. High confidence additive linear cis eQTLs from the LPS treatment. Table S6. Low confidence additive linear cis eQTLs from the LPS treatment. Table S7. High confidence additive linear trans eQTLs from the LPS treatment. Table S8. Low confidence additive linear trans eQTLs from the LPS treatment. Table S9. High confidence additive linear cis eQTLs from the RCA treatment. Table S10. Low confidence additive linear cis eQTLs from the RCA treatment. Table S11. High confidence additive linear trans eQTLs from the RCA treatment. The eQTLs reported are limited to one eQTL per gene, representing the eSNP with the lowest FDR value for each gene. Table S12. Low confidence additive linear trans eQTLs from the RCA treatment. Table S13. High confidence additive linear cis eQTLs from the HDE treatment. Table S14. Low confidence additive linear cis eQTLs from the HDE [file 12864_2018_4938_MOESM1_ESM.zip › S9_Fig.tif]
