# Supplementary material for: eQTL discovery and their association with severe equine asthma in European Warmblood horses
Source: BMC Genomics. 2018 Aug 2;19:581. doi: 10.1186/s12864-018-4938-9 (PMC6090848; doi:10.1186/s12864-018-4938-9)
Supplement: Supplementary file 2 — Linear mixed models jointly modeling MCK and HDE. Linear mixed models with random intercepts for each individual model the association between the top fifteen RAO associated SNPs that were also eSNPs in either MCK or HDE (chr13.32843309, chr13.32844446, chr13.33460982, chr13.33502488, chr28.3692072, chr21.52625145) and the gene expression of the genes they regulated (DEXI, NSUN2, ATF7IP2, GLIPR1L2) with reduced maximum likelihood (REML) and maximum likelihood (ML). An R markdown document that generated this html file is available on GitHub: https://github.com/VCMason. (HTML 5545 kb) [file 12864_2018_4938_MOESM2_ESM.html]

JointModelingOfMCKAndHDE


# JointModelingOfMCKAndHDE

#### *Victor Mason*

#### *11 Januar 2018*

## Read in data

```
.pardefault <- par(no.readonly = T) #save default graphical parameters

mck.genes <- read.table(file="D:\\LinuxShare\\Programs\\MatrixEQTL\\FormatDataIN\\MCK1_no559443_1056195_Fix111\\DESeq.NCBI.MCK1.m20.wVSD.AgeAsNumericFix111.tx.tab", sep="\t", header=TRUE, check.names = FALSE) # mock (no treatment) gene expression (normalized and variance stabalized after KS gene filter)
mck.covs <- read.table(file="D:\\LinuxShare\\Programs\\MatrixEQTL\\FormatDataIN\\MCK1_no559443_1056195_Fix111\\RAO_database.VCM.v4.MCK1.no559443.MatrixeQTL.ForR.csv", sep=",", header=TRUE, check.names = FALSE) # mock (no treatment) independent variables

hde.genes <- read.table(file="D:\\LinuxShare\\Programs\\MatrixEQTL\\FormatDataIN\\HDE9_no559443_1056195_Fix111\\DESeq.NCBI.HDE9.m24.wVSD.AgeAsNumericFix111.tx.tab", sep="\t", header=TRUE, check.names = FALSE)# HDE treatment gene expression (normalized and variance stabalized after KS gene filter)
hde.covs <- read.table(file="D:\\LinuxShare\\Programs\\MatrixEQTL\\FormatDataIN\\HDE9_no559443_1056195_Fix111\\RAO_database.VCM.v4.HDE9.no559443.MatrixeQTL.w4SVLeek.ForR.csv", sep=",", header=TRUE, check.names = FALSE) #HDE treatment independent variables

all.snps <- read.table(file="D:\\LinuxShare\\Programs\\MatrixEQTL\\FormatDataIN\\HDE9_no559443_1056195_Fix111\\82_HDE9_RAO_HD_Warmbloods.ne1000.EC2.379MAF0.05.168ControlsHWE1e6.012.1056195.gen.RNA.ForR.sort.ForR.tsv", sep="\t", header=TRUE, check.names = FALSE) # this file has SNPs for all individuals
```

## Construct dataframe for analysis

```
MakeDF <- function(snp, gene, common.covs, m.covs, h.covs)
{
  
s <- as.data.frame(all.snps[snp,])

mck.g <- as.data.frame(mck.genes[gene,]) #"DEXI"
hde.g <- as.data.frame(hde.genes[gene,]) #"DEXI"

rownames(mck.g)[1] <- "gene" #standardize gene names in model
rownames(hde.g)[1] <- "gene" #standardize gene names in model

### trim to same individuals ###
m.s <- s[,match(colnames(mck.g), colnames(s))] #match individuals with expression data in MCK to to position in all individuals with SNP data.
h.s <- s[,match(colnames(hde.g), colnames(s))] #match individuals with expression data in HDE to to position in all individuals with SNP data.
rownames(m.s)[1] <- "snp" #standardize snp names in model
rownames(h.s)[1] <- "snp" #standardize snp names in model

m.c <- mck.covs[,match(colnames(mck.g), colnames(mck.covs))] #match individuals with expression data in MCK individuals with covariate data.
h.c <- hde.covs[,match(colnames(hde.g), colnames(hde.covs))] #match individuals with expression data in HDE individuals with covariate data.


#for MCK (baseline)
dim(m.c)
m.c <- m.c[rownames(m.c) %in% common.covs,]
dim(m.c)
df <- rbind(mck.g, m.s, m.c)
dim(df)

t.df <- as.data.frame(t(df))
dim(t.df)
t.df$Treat=0
t.df$ID=rownames(t.df)
dim(t.df)

#for HDE (treated)
dim(h.c)
h.c <- h.c[rownames(h.c) %in% common.covs,]
dim(h.c)
h.df <- rbind(hde.g, h.s, h.c)
dim(h.df)

h.t.df <- as.data.frame(t(h.df))
dim(h.t.df)
h.t.df$Treat=1
h.t.df$ID=rownames(h.t.df)
dim(h.t.df)

comb.df <- rbind(as.data.frame(t.df), as.data.frame(h.t.df))
str(comb.df)
comb.df$ID <- factor(comb.df$ID)
str(comb.df)

return(comb.df)
  
}
```

## Fit logistic regression and make plots

### Model:

GeneExpression ~ Fam1 + Fam2 + Mare + Age + Genotype\*Treatment\*DiseaseStatus + (1|ID)

GeneExpression = normalized and variance stabilized gene expression value for genes not removed by the KS test for both treatments MCK and HDE Fam1 = 0 (unrelated horses) or 1 (Family 1 horses) Fam2 = 0 (unrelated horses) or 1 (Family 2 horses) Mare = 0 (male (stallion or gelding)) or1 (Mare) Age = year of sample collection - year of birth (a quantitative variable) Genotype = snp = 0,1,2 Treatment = Treat = 0 (MCK), or 1 (HDE) DiseaseStatus = RNAseq\_condition = 0 (healthy), or 1 (RAO horse) ID = individual ID (two measurements per individual, one per treatment)

```
FitModel <- function(comb.df, snp, gene)
{
print(snp)
print(gene)

#fit mixed model with random intercept for each idividual's ID using reduced maximum likelihood (REML) # No pvalues
library(lme4)
#gene is name of column with gene expression values both for MCK and HDE
mod <- lmer(gene ~ Fam1 + Fam2 + Mare + Age + snp*Treat*RNAseq_condition + (1|ID), data=comb.df)
#mod <- glmer(RNAseq_condition ~ gene*Treat + (1|ID), data=comb.df, family=binomial(link="logit"))
#mod <- glmer(DEXI ~ RNAseq_condition*Treat + (1|ID), data=comb.df)
#mod <- glmer(RNAseq_condition ~ DEXI + (1|ID) + (1|Treat), data=comb.df, family=binomial(link="logit"))
print(summary(mod))
#print(confint(mod, oldNames=FALSE))
#print(confint(mod, method="boot", nsim=100, parm=4:8))

#fit mixed model with random intercept for each idividual's ID using maximum likelihood (ML) # This also reports pvalues
library(nlme)
mod2 <- lme(gene~ Fam1 + Fam2 + Mare + Age + snp*Treat*RNAseq_condition, random=~1|ID, method="ML", data=comb.df)
print(summary(mod2))
#print(anova(mod2))

#install.packages("coda")
#install.packages("coefplot2", repos="http://www.math.mcmaster.ca/bolker/R", type="source")
library(coefplot2)
print("Fixed Effect Regression Estimates of Mixed Model with REML")
title <- paste(snp,gene,"Regression Estimates", sep=" ")
coefplot2(mod, main=title)

title <- paste(snp,gene,"eQTL","Color=Treatment", sep=" ")
plot(comb.df$snp, comb.df$gene, col=comb.df$Treat+1, main=title, xlab="Genotype", ylab="Gene Expression")
title <- paste(snp,gene,"Color=Treatment", sep=" ")
boxplot(gene ~ snp*Treat, col=(c("blue", "blue", "blue", "red", "red", "red")), data=comb.df, main=title, xlab="snp*Treat", ylab="Gene Expression") # outpch = NA
stripchart(gene ~ snp*Treat, data = comb.df, vertical = TRUE, method = "jitter", pch = 21, add = TRUE) #col = comb.df$Treat+1, bg = comb.df$Treat+1, 

title <- paste(snp,gene,"eQTL","Color=DiseaseStatus", sep=" ")
plot(comb.df$snp, comb.df$gene, col=comb.df$RNAseq_condition+1, main=title, xlab="Genotype", ylab="Gene Expression")
title <- paste(snp,gene,"Color=DiseaseStatus", sep=" ")
boxplot(gene ~ snp*RNAseq_condition, col=(c("blue", "blue", "blue", "red", "red", "red")), main=title, data=comb.df, xlab="snp*DiseaseStatus", ylab="Gene Expression")
stripchart(gene ~ snp*RNAseq_condition, data = comb.df, vertical = TRUE, method = "jitter", pch = 21, add = TRUE)
#plot(comb.df$RNAseq_condition, comb.df$DEXI, col=comb.df$Treat+1)

# fit Multiple linear regression (no random effects)
mod3 <- lm(gene ~ Fam1 + Fam2 + Mare + Age + snp*Treat*RNAseq_condition, data=comb.df)
summary(mod3)
print("Added Variable aka. Partial Regression Plots: for multiple linear regression (no random effects)")
library(car)
par(mar = rep(2, 4))
avPlots(mod3, layout = c(3,3)) #terms = ~ Fam1 + Fam2 + snp + Treat + snp:Treat

par(.pardefault)

}
```

## Make DFs for sepcific SNP, gene, and covariates

## Fit Model for each gene/SNP Pair

```
genes <- c("DEXI", "NSUN2", "ATF7IP2", "GLIPR1L2")
common.covs <- c("Fam1", "Fam2", "Mare", "Age", "RNAseq_condition") #Treat

snps <- c("chr13.32843309", "chr13.32844446", "chr13.33460982", "chr13.33502488", "chr28.3692072", "chr21.52625145") #"chr13.33525948", 
for ( snp in snps )
{
  for ( gene in genes )
  {
  
  comb.df <- MakeDF(snp, gene, common.covs, mck.covs, hde.covs)
  
  FitModel(comb.df, snp, gene)
  
  }
  
}
```

```
## 'data.frame':    155 obs. of  9 variables:
##  $ gene            : num  8.52 8.31 8.42 8.44 8.2 ...
##  $ snp             : num  0 1 1 1 1 0 0 1 1 1 ...
##  $ Fam1            : num  1 0 0 0 1 1 1 1 1 0 ...
##  $ Fam2            : num  0 1 1 1 0 0 0 0 0 1 ...
##  $ Mare            : num  1 1 1 0 0 0 0 1 1 0 ...
##  $ Age             : num  18 18 13 18 19 17 20 19 15 15 ...
##  $ RNAseq_condition: num  0 1 0 0 1 1 1 1 0 0 ...
##  $ Treat           : num  0 0 0 0 0 0 0 0 0 0 ...
##  $ ID              : chr  "26" "29" "30" "31" ...
## 'data.frame':    155 obs. of  9 variables:
##  $ gene            : num  8.52 8.31 8.42 8.44 8.2 ...
##  $ snp             : num  0 1 1 1 1 0 0 1 1 1 ...
##  $ Fam1            : num  1 0 0 0 1 1 1 1 1 0 ...
##  $ Fam2            : num  0 1 1 1 0 0 0 0 0 1 ...
##  $ Mare            : num  1 1 1 0 0 0 0 1 1 0 ...
##  $ Age             : num  18 18 13 18 19 17 20 19 15 15 ...
##  $ RNAseq_condition: num  0 1 0 0 1 1 1 1 0 0 ...
##  $ Treat           : num  0 0 0 0 0 0 0 0 0 0 ...
##  $ ID              : Factor w/ 82 levels "100","110","111",..: 5 6 7 8 10 12 13 14 16 17 ...
## [1] "chr13.32843309"
## [1] "DEXI"
```

```
## Loading required package: Matrix
```

```
## Linear mixed model fit by REML ['lmerMod']
## Formula: 
## gene ~ Fam1 + Fam2 + Mare + Age + snp * Treat * RNAseq_condition +  
##     (1 | ID)
##    Data: comb.df
## 
## REML criterion at convergence: -79.5
## 
## Scaled residuals: 
##      Min       1Q   Median       3Q      Max 
## -1.94611 -0.62213  0.05781  0.54204  2.06947 
## 
## Random effects:
##  Groups   Name        Variance Std.Dev.
##  ID       (Intercept) 0.007957 0.0892  
##  Residual             0.018214 0.1350  
## Number of obs: 155, groups:  ID, 82
## 
## Fixed effects:
##                             Estimate Std. Error t value
## (Intercept)                 8.238946   0.093374   88.24
## Fam1                        0.177845   0.040382    4.40
## Fam2                        0.147033   0.042268    3.48
## Mare                        0.005601   0.032254    0.17
## Age                        -0.006223   0.004458   -1.40
## snp                        -0.116854   0.039507   -2.96
## Treat                      -0.165701   0.054607   -3.03
## RNAseq_condition           -0.070009   0.065303   -1.07
## snp:Treat                   0.171482   0.043967    3.90
## snp:RNAseq_condition        0.043424   0.055278    0.79
## Treat:RNAseq_condition      0.118800   0.073319    1.62
## snp:Treat:RNAseq_condition -0.117842   0.063346   -1.86
## 
## Correlation of Fixed Effects:
##             (Intr) Fam1   Fam2   Mare   Age    snp    Treat  RNAsq_ snp:Tr
## Fam1        -0.112                                                        
## Fam2        -0.373  0.280                                                 
## Mare        -0.040 -0.065 -0.050                                          
## Age         -0.814 -0.070  0.200 -0.122                                   
## snp         -0.425  0.201  0.217 -0.046 -0.053                            
## Treat       -0.313 -0.002  0.027 -0.012 -0.018  0.528                     
## RNAsq_cndtn -0.470  0.007  0.135  0.174  0.064  0.601  0.465              
## snp:Treat    0.270  0.005 -0.021  0.015  0.006 -0.616 -0.839 -0.391       
## snp:RNAsq_c  0.335 -0.028 -0.077 -0.138 -0.003 -0.674 -0.372 -0.800  0.437
## Trt:RNAsq_c  0.255 -0.030 -0.047  0.010 -0.003 -0.401 -0.745 -0.630  0.625
## snp:Tr:RNA_ -0.203  0.014  0.027 -0.022  0.013  0.432  0.582  0.495 -0.694
##             s:RNA_ T:RNA_
## Fam1                     
## Fam2                     
## Mare                     
## Age                      
## snp                      
## Treat                    
## RNAsq_cndtn              
## snp:Treat                
## snp:RNAsq_c              
## Trt:RNAsq_c  0.508       
## snp:Tr:RNA_ -0.620 -0.792
```

```
## 
## Attaching package: 'nlme'
```

```
## The following object is masked from 'package:lme4':
## 
##     lmList
```

```
## Linear mixed-effects model fit by maximum likelihood
##  Data: comb.df 
##         AIC       BIC   logLik
##   -116.4335 -73.82551 72.21673
## 
## Random effects:
##  Formula: ~1 | ID
##         (Intercept)  Residual
## StdDev:  0.08152026 0.1315307
## 
## Fixed effects: gene ~ Fam1 + Fam2 + Mare + Age + snp * Treat * RNAseq_condition 
##                                Value  Std.Error DF  t-value p-value
## (Intercept)                 8.239232 0.09231602 74 89.25029  0.0000
## Fam1                        0.177389 0.03984794 74  4.45165  0.0000
## Fam2                        0.146921 0.04170079 74  3.52323  0.0007
## Mare                        0.005377 0.03181488 74  0.16900  0.8663
## Age                        -0.006193 0.00439624 74 -1.40869  0.1631
## snp                        -0.117313 0.03936412 74 -2.98019  0.0039
## Treat                      -0.166223 0.05534494 69 -3.00340  0.0037
## RNAseq_condition           -0.070882 0.06509356 74 -1.08892  0.2797
## snp:Treat                   0.171841 0.04456573 69  3.85590  0.0003
## snp:RNAseq_condition        0.044035 0.05508656 74  0.79938  0.4266
## Treat:RNAseq_condition      0.119622 0.07430609 69  1.60986  0.1120
## snp:Treat:RNAseq_condition -0.118377 0.06421922 69 -1.84332  0.0696
##  Correlation: 
##                            (Intr) Fam1   Fam2   Mare   Age    snp   
## Fam1                       -0.112                                   
## Fam2                       -0.372  0.280                            
## Mare                       -0.040 -0.065 -0.050                     
## Age                        -0.811 -0.070  0.200 -0.123              
## snp                        -0.429  0.199  0.215 -0.046 -0.053       
## Treat                      -0.320 -0.002  0.027 -0.012 -0.018  0.536
## RNAseq_condition           -0.473  0.008  0.136  0.172  0.063  0.602
## snp:Treat                   0.276  0.005 -0.021  0.015  0.006 -0.626
## snp:RNAseq_condition        0.337 -0.028 -0.078 -0.136 -0.004 -0.674
## Treat:RNAseq_condition      0.261 -0.031 -0.047  0.010 -0.003 -0.407
## snp:Treat:RNAseq_condition -0.208  0.014  0.027 -0.023  0.013  0.438
##                            Treat  RNAsq_ snp:Tr s:RNA_ T:RNA_
## Fam1                                                         
## Fam2                                                         
## Mare                                                         
## Age                                                          
## snp                                                          
## Treat                                                        
## RNAseq_condition            0.472                            
## snp:Treat                  -0.839 -0.397                     
## snp:RNAseq_condition       -0.378 -0.800  0.443              
## Treat:RNAseq_condition     -0.745 -0.640  0.625  0.516       
## snp:Treat:RNAseq_condition  0.582  0.502 -0.694 -0.630 -0.792
## 
## Standardized Within-Group Residuals:
##         Min          Q1         Med          Q3         Max 
## -2.07498384 -0.65028828  0.06784211  0.55422344  2.20555084 
## 
## Number of Observations: 155
## Number of Groups: 82
```

```
## Loading required package: coda
```

```
## [1] "Fixed Effect Regression Estimates of Mixed Model with REML"
```

```
## [1] "Added Variable aka. Partial Regression Plots: for multiple linear regression (no random effects)"
```

```
## 'data.frame':    155 obs. of  9 variables:
##  $ gene            : num  10.01 9.99 10.15 10 9.99 ...
##  $ snp             : num  0 1 1 1 1 0 0 1 1 1 ...
##  $ Fam1            : num  1 0 0 0 1 1 1 1 1 0 ...
##  $ Fam2            : num  0 1 1 1 0 0 0 0 0 1 ...
##  $ Mare            : num  1 1 1 0 0 0 0 1 1 0 ...
##  $ Age             : num  18 18 13 18 19 17 20 19 15 15 ...
##  $ RNAseq_condition: num  0 1 0 0 1 1 1 1 0 0 ...
##  $ Treat           : num  0 0 0 0 0 0 0 0 0 0 ...
##  $ ID              : chr  "26" "29" "30" "31" ...
## 'data.frame':    155 obs. of  9 variables:
##  $ gene            : num  10.01 9.99 10.15 10 9.99 ...
##  $ snp             : num  0 1 1 1 1 0 0 1 1 1 ...
##  $ Fam1            : num  1 0 0 0 1 1 1 1 1 0 ...
##  $ Fam2            : num  0 1 1 1 0 0 0 0 0 1 ...
##  $ Mare            : num  1 1 1 0 0 0 0 1 1 0 ...
##  $ Age             : num  18 18 13 18 19 17 20 19 15 15 ...
##  $ RNAseq_condition: num  0 1 0 0 1 1 1 1 0 0 ...
##  $ Treat           : num  0 0 0 0 0 0 0 0 0 0 ...
##  $ ID              : Factor w/ 82 levels "100","110","111",..: 5 6 7 8 10 12 13 14 16 17 ...
## [1] "chr13.32843309"
## [1] "NSUN2"
## Linear mixed model fit by REML ['lmerMod']
## Formula: 
## gene ~ Fam1 + Fam2 + Mare + Age + snp * Treat * RNAseq_condition +  
##     (1 | ID)
##    Data: comb.df
## 
## REML criterion at convergence: -216
## 
## Scaled residuals: 
##     Min      1Q  Median      3Q     Max 
## -1.7768 -0.4867  0.0438  0.5007  1.7597 
## 
## Random effects:
##  Groups   Name        Variance Std.Dev.
##  ID       (Intercept) 0.006507 0.08066 
##  Residual             0.005089 0.07133 
## Number of obs: 155, groups:  ID, 82
## 
## Fixed effects:
##                             Estimate Std. Error t value
## (Intercept)                10.141408   0.066256  153.06
## Fam1                       -0.038947   0.029099   -1.34
## Fam2                       -0.077626   0.030516   -2.54
## Mare                        0.001483   0.023329    0.06
## Age                        -0.002877   0.003233   -0.89
## snp                        -0.013560   0.026017   -0.52
## Treat                       0.128455   0.029172    4.40
## RNAseq_condition           -0.022118   0.042804   -0.52
## snp:Treat                   0.034502   0.023466    1.47
## snp:RNAseq_condition        0.008825   0.036394    0.24
## Treat:RNAseq_condition      0.025395   0.039189    0.65
## snp:Treat:RNAseq_condition -0.049085   0.033756   -1.45
## 
## Correlation of Fixed Effects:
##             (Intr) Fam1   Fam2   Mare   Age    snp    Treat  RNAsq_ snp:Tr
## Fam1        -0.113                                                        
## Fam2        -0.377  0.284                                                 
## Mare        -0.044 -0.072 -0.055                                          
## Age         -0.837 -0.071  0.204 -0.119                                   
## snp         -0.391  0.223  0.228 -0.046 -0.055                            
## Treat       -0.238 -0.001  0.023 -0.010 -0.016  0.437                     
## RNAsq_cndtn -0.448 -0.007  0.127  0.197  0.070  0.590  0.385              
## snp:Treat    0.207  0.004 -0.018  0.013  0.005 -0.508 -0.841 -0.325       
## snp:RNAsq_c  0.308 -0.023 -0.071 -0.162  0.002 -0.665 -0.308 -0.798  0.359
## Trt:RNAsq_c  0.196 -0.026 -0.040  0.009 -0.003 -0.333 -0.744 -0.523  0.626
## snp:Tr:RNA_ -0.158  0.012  0.023 -0.019  0.011  0.356  0.584  0.412 -0.695
##             s:RNA_ T:RNA_
## Fam1                     
## Fam2                     
## Mare                     
## Age                      
## snp                      
## Treat                    
## RNAsq_cndtn              
## snp:Treat                
## snp:RNAsq_c              
## Trt:RNAsq_c  0.420       
## snp:Tr:RNA_ -0.508 -0.795
## Linear mixed-effects model fit by maximum likelihood
##  Data: comb.df 
##         AIC       BIC   logLik
##   -263.1389 -220.5309 145.5694
## 
## Random effects:
##  Formula: ~1 | ID
##         (Intercept)   Residual
## StdDev:  0.07550055 0.06953203
## 
## Fixed effects: gene ~ Fam1 + Fam2 + Mare + Age + snp * Treat * RNAseq_condition 
##                                Value  Std.Error DF   t-value p-value
## (Intercept)                10.141796 0.06544796 74 154.95970  0.0000
## Fam1                       -0.039038 0.02871487 74  -1.35950  0.1781
## Fam2                       -0.077652 0.03010861 74  -2.57907  0.0119
## Mare                        0.001391 0.02301390 74   0.06044  0.9520
## Age                        -0.002884 0.00318892 74  -0.90452  0.3687
## snp                        -0.013896 0.02584979 74  -0.53757  0.5925
## Treat                       0.128274 0.02957997 69   4.33651  0.0000
## RNAseq_condition           -0.022438 0.04254670 74  -0.52738  0.5995
## snp:Treat                   0.034828 0.02379531 69   1.46365  0.1478
## snp:RNAseq_condition        0.009163 0.03615838 74   0.25343  0.8006
## Treat:RNAseq_condition      0.025674 0.03973534 69   0.64614  0.5203
## snp:Treat:RNAseq_condition -0.049394 0.03423400 69  -1.44285  0.1536
##  Correlation: 
##                            (Intr) Fam1   Fam2   Mare   Age    snp   
## Fam1                       -0.113                                   
## Fam2                       -0.377  0.283                            
## Mare                       -0.044 -0.072 -0.055                     
## Age                        -0.835 -0.071  0.203 -0.120              
## snp                        -0.393  0.221  0.227 -0.046 -0.055       
## Treat                      -0.244 -0.001  0.023 -0.010 -0.016  0.445
## RNAseq_condition           -0.450 -0.006  0.128  0.195  0.070  0.591
## snp:Treat                   0.212  0.004 -0.018  0.013  0.005 -0.518
## snp:RNAseq_condition        0.310 -0.024 -0.072 -0.160  0.001 -0.666
## Treat:RNAseq_condition      0.201 -0.026 -0.040  0.009 -0.003 -0.339
## snp:Treat:RNAseq_condition -0.162  0.012  0.023 -0.020  0.011  0.363
##                            Treat  RNAsq_ snp:Tr s:RNA_ T:RNA_
## Fam1                                                         
## Fam2                                                         
## Mare                                                         
## Age                                                          
## snp                                                          
## Treat                                                        
## RNAseq_condition            0.392                            
## snp:Treat                  -0.841 -0.331                     
## snp:RNAseq_condition       -0.314 -0.798  0.366              
## Treat:RNAseq_condition     -0.744 -0.533  0.626  0.428       
## snp:Treat:RNAseq_condition  0.584  0.419 -0.695 -0.518 -0.794
## 
## Standardized Within-Group Residuals:
##         Min          Q1         Med          Q3         Max 
## -1.83118019 -0.50305674  0.04440007  0.51724220  1.86152224 
## 
## Number of Observations: 155
## Number of Groups: 82 
## [1] "Fixed Effect Regression Estimates of Mixed Model with REML"
```

```
## [1] "Added Variable aka. Partial Regression Plots: for multiple linear regression (no random effects)"
```

```
## 'data.frame':    155 obs. of  9 variables:
##  $ gene            : num  7.27 6.57 6.05 6.37 6.02 ...
##  $ snp             : num  0 1 1 1 1 0 0 1 1 1 ...
##  $ Fam1            : num  1 0 0 0 1 1 1 1 1 0 ...
##  $ Fam2            : num  0 1 1 1 0 0 0 0 0 1 ...
##  $ Mare            : num  1 1 1 0 0 0 0 1 1 0 ...
##  $ Age             : num  18 18 13 18 19 17 20 19 15 15 ...
##  $ RNAseq_condition: num  0 1 0 0 1 1 1 1 0 0 ...
##  $ Treat           : num  0 0 0 0 0 0 0 0 0 0 ...
##  $ ID              : chr  "26" "29" "30" "31" ...
## 'data.frame':    155 obs. of  9 variables:
##  $ gene            : num  7.27 6.57 6.05 6.37 6.02 ...
##  $ snp             : num  0 1 1 1 1 0 0 1 1 1 ...
##  $ Fam1            : num  1 0 0 0 1 1 1 1 1 0 ...
##  $ Fam2            : num  0 1 1 1 0 0 0 0 0 1 ...
##  $ Mare            : num  1 1 1 0 0 0 0 1 1 0 ...
##  $ Age             : num  18 18 13 18 19 17 20 19 15 15 ...
##  $ RNAseq_condition: num  0 1 0 0 1 1 1 1 0 0 ...
##  $ Treat           : num  0 0 0 0 0 0 0 0 0 0 ...
##  $ ID              : Factor w/ 82 levels "100","110","111",..: 5 6 7 8 10 12 13 14 16 17 ...
## [1] "chr13.32843309"
## [1] "ATF7IP2"
## Linear mixed model fit by REML ['lmerMod']
## Formula: 
## gene ~ Fam1 + Fam2 + Mare + Age + snp * Treat * RNAseq_condition +  
##     (1 | ID)
##    Data: comb.df
## 
## REML criterion at convergence: 270.3
## 
## Scaled residuals: 
##      Min       1Q   Median       3Q      Max 
## -2.85467 -0.45671 -0.09696  0.42088  2.39706 
## 
## Random effects:
##  Groups   Name        Variance Std.Dev.
##  ID       (Intercept) 0.36704  0.6058  
##  Residual             0.09815  0.3133  
## Number of obs: 155, groups:  ID, 82
## 
## Fixed effects:
##                            Estimate Std. Error t value
## (Intercept)                 6.04132    0.44060  13.712
## Fam1                       -0.08214    0.19551  -0.420
## Fam2                        0.31560    0.20536   1.537
## Mare                       -0.07788    0.15724  -0.495
## Age                        -0.01078    0.02184  -0.494
## snp                         0.51816    0.16182   3.202
## Treat                       0.58483    0.12930   4.523
## RNAseq_condition            0.65742    0.26476   2.483
## snp:Treat                  -0.20237    0.10393  -1.947
## snp:RNAseq_condition       -0.47466    0.22671  -2.094
## Treat:RNAseq_condition     -0.32104    0.17377  -1.848
## snp:Treat:RNAseq_condition  0.31349    0.14930   2.100
## 
## Correlation of Fixed Effects:
##             (Intr) Fam1   Fam2   Mare   Age    snp    Treat  RNAsq_ snp:Tr
## Fam1        -0.112                                                        
## Fam2        -0.379  0.286                                                 
## Mare        -0.047 -0.078 -0.059                                          
## Age         -0.854 -0.072  0.207 -0.117                                   
## snp         -0.361  0.244  0.238 -0.045 -0.057                            
## Treat       -0.160 -0.001  0.017 -0.007 -0.011  0.316                     
## RNAsq_cndtn -0.429 -0.022  0.117  0.219  0.076  0.577  0.279              
## snp:Treat    0.140  0.003 -0.013  0.009  0.004 -0.367 -0.842 -0.236       
## snp:RNAsq_c  0.283 -0.018 -0.064 -0.185  0.007 -0.655 -0.222 -0.793  0.259
## Trt:RNAsq_c  0.133 -0.019 -0.029  0.007 -0.002 -0.241 -0.744 -0.380  0.627
## snp:Tr:RNA_ -0.108  0.009  0.017 -0.014  0.008  0.258  0.586  0.300 -0.696
##             s:RNA_ T:RNA_
## Fam1                     
## Fam2                     
## Mare                     
## Age                      
## snp                      
## Treat                    
## RNAsq_cndtn              
## snp:Treat                
## snp:RNAsq_c              
## Trt:RNAsq_c  0.303       
## snp:Tr:RNA_ -0.365 -0.796
## Linear mixed-effects model fit by maximum likelihood
##  Data: comb.df 
##        AIC      BIC    logLik
##   265.5925 308.2004 -118.7962
## 
## Random effects:
##  Formula: ~1 | ID
##         (Intercept)  Residual
## StdDev:    0.573363 0.3046528
## 
## Fixed effects: gene ~ Fam1 + Fam2 + Mare + Age + snp * Treat * RNAseq_condition 
##                                Value Std.Error DF   t-value p-value
## (Intercept)                 6.041429 0.4358705 74 13.860606  0.0000
## Fam1                       -0.082043 0.1933409 74 -0.424341  0.6725
## Fam2                        0.315543 0.2030659 74  1.553893  0.1245
## Mare                       -0.077932 0.1554730 74 -0.501256  0.6177
## Age                        -0.010775 0.0215954 74 -0.498971  0.6193
## snp                         0.518444 0.1605249 74  3.229682  0.0018
## Treat                       0.584665 0.1308585 69  4.467914  0.0000
## RNAseq_condition            0.657064 0.2627017 74  2.501178  0.0146
## snp:Treat                  -0.202656 0.1051815 69 -1.926724  0.0581
## snp:RNAseq_condition       -0.474616 0.2248707 74 -2.110617  0.0382
## Treat:RNAseq_condition     -0.320712 0.1758569 69 -1.823712  0.0725
## snp:Treat:RNAseq_condition  0.313455 0.1511076 69  2.074384  0.0418
##  Correlation: 
##                            (Intr) Fam1   Fam2   Mare   Age    snp   
## Fam1                       -0.112                                   
## Fam2                       -0.379  0.286                            
## Mare                       -0.047 -0.078 -0.059                     
## Age                        -0.854 -0.072  0.207 -0.117              
## snp                        -0.362  0.243  0.238 -0.045 -0.057       
## Treat                      -0.164 -0.001  0.017 -0.007 -0.012  0.323
## RNAseq_condition           -0.430 -0.022  0.117  0.218  0.076  0.577
## snp:Treat                   0.143  0.003 -0.013  0.010  0.004 -0.374
## snp:RNAseq_condition        0.284 -0.018 -0.065 -0.184  0.006 -0.656
## Treat:RNAseq_condition      0.136 -0.019 -0.030  0.007 -0.002 -0.246
## snp:Treat:RNAseq_condition -0.110  0.009  0.017 -0.014  0.008  0.263
##                            Treat  RNAsq_ snp:Tr s:RNA_ T:RNA_
## Fam1                                                         
## Fam2                                                         
## Mare                                                         
## Age                                                          
## snp                                                          
## Treat                                                        
## RNAseq_condition            0.285                            
## snp:Treat                  -0.842 -0.241                     
## snp:RNAseq_condition       -0.227 -0.794  0.264              
## Treat:RNAseq_condition     -0.744 -0.388  0.627  0.309       
## snp:Treat:RNAseq_condition  0.586  0.306 -0.696 -0.372 -0.796
## 
## Standardized Within-Group Residuals:
##         Min          Q1         Med          Q3         Max 
## -2.94791193 -0.47853398 -0.09107154  0.43859080  2.45418809 
## 
## Number of Observations: 155
## Number of Groups: 82 
## [1] "Fixed Effect Regression Estimates of Mixed Model with REML"
```

```
## [1] "Added Variable aka. Partial Regression Plots: for multiple linear regression (no random effects)"
```

```
## 'data.frame':    155 obs. of  9 variables:
##  $ gene            : num  7.63 7.92 7.58 7.29 8 ...
##  $ snp             : num  0 1 1 1 1 0 0 1 1 1 ...
##  $ Fam1            : num  1 0 0 0 1 1 1 1 1 0 ...
##  $ Fam2            : num  0 1 1 1 0 0 0 0 0 1 ...
##  $ Mare            : num  1 1 1 0 0 0 0 1 1 0 ...
##  $ Age             : num  18 18 13 18 19 17 20 19 15 15 ...
##  $ RNAseq_condition: num  0 1 0 0 1 1 1 1 0 0 ...
##  $ Treat           : num  0 0 0 0 0 0 0 0 0 0 ...
##  $ ID              : chr  "26" "29" "30" "31" ...
## 'data.frame':    155 obs. of  9 variables:
##  $ gene            : num  7.63 7.92 7.58 7.29 8 ...
##  $ snp             : num  0 1 1 1 1 0 0 1 1 1 ...
##  $ Fam1            : num  1 0 0 0 1 1 1 1 1 0 ...
##  $ Fam2            : num  0 1 1 1 0 0 0 0 0 1 ...
##  $ Mare            : num  1 1 1 0 0 0 0 1 1 0 ...
##  $ Age             : num  18 18 13 18 19 17 20 19 15 15 ...
##  $ RNAseq_condition: num  0 1 0 0 1 1 1 1 0 0 ...
##  $ Treat           : num  0 0 0 0 0 0 0 0 0 0 ...
##  $ ID              : Factor w/ 82 levels "100","110","111",..: 5 6 7 8 10 12 13 14 16 17 ...
## [1] "chr13.32843309"
## [1] "GLIPR1L2"
## Linear mixed model fit by REML ['lmerMod']
## Formula: 
## gene ~ Fam1 + Fam2 + Mare + Age + snp * Treat * RNAseq_condition +  
##     (1 | ID)
##    Data: comb.df
## 
## REML criterion at convergence: 108
## 
## Scaled residuals: 
##      Min       1Q   Median       3Q      Max 
## -2.19960 -0.48513 -0.03029  0.48080  2.03637 
## 
## Random effects:
##  Groups   Name        Variance Std.Dev.
##  ID       (Intercept) 0.07024  0.2650  
##  Residual             0.04586  0.2142  
## Number of obs: 155, groups:  ID, 82
## 
## Fixed effects:
##                             Estimate Std. Error t value
## (Intercept)                 8.131780   0.211735   38.41
## Fam1                       -0.324959   0.093196   -3.49
## Fam2                       -0.373073   0.097764   -3.82
## Mare                       -0.022087   0.074761   -0.30
## Age                        -0.004321   0.010366   -0.42
## snp                         0.013301   0.082092    0.16
## Treat                      -0.059593   0.087734   -0.68
## RNAseq_condition           -0.144231   0.134930   -1.07
## snp:Treat                   0.003297   0.070561    0.05
## snp:RNAseq_condition       -0.057418   0.114853   -0.50
## Treat:RNAseq_condition     -0.016000   0.117869   -0.14
## snp:Treat:RNAseq_condition  0.092922   0.101476    0.92
## 
## Correlation of Fixed Effects:
##             (Intr) Fam1   Fam2   Mare   Age    snp    Treat  RNAsq_ snp:Tr
## Fam1        -0.113                                                        
## Fam2        -0.377  0.284                                                 
## Mare        -0.045 -0.073 -0.056                                          
## Age         -0.840 -0.071  0.204 -0.119                                   
## snp         -0.385  0.227  0.230 -0.046 -0.056                            
## Treat       -0.224 -0.001  0.022 -0.010 -0.015  0.418                     
## RNAsq_cndtn -0.444 -0.010  0.125  0.201  0.071  0.587  0.368              
## snp:Treat    0.195  0.003 -0.017  0.012  0.005 -0.485 -0.841 -0.311       
## snp:RNAsq_c  0.303 -0.022 -0.070 -0.166  0.002 -0.663 -0.294 -0.797  0.343
## Trt:RNAsq_c  0.186 -0.025 -0.038  0.009 -0.003 -0.318 -0.744 -0.500  0.626
## snp:Tr:RNA_ -0.149  0.012  0.022 -0.019  0.010  0.341  0.585  0.394 -0.695
##             s:RNA_ T:RNA_
## Fam1                     
## Fam2                     
## Mare                     
## Age                      
## snp                      
## Treat                    
## RNAsq_cndtn              
## snp:Treat                
## snp:RNAsq_c              
## Trt:RNAsq_c  0.401       
## snp:Tr:RNA_ -0.485 -0.795
## Linear mixed-effects model fit by maximum likelihood
##  Data: comb.df 
##        AIC      BIC    logLik
##   88.30374 130.9117 -30.15187
## 
## Random effects:
##  Formula: ~1 | ID
##         (Intercept)  Residual
## StdDev:   0.2493323 0.2083527
## 
## Fixed effects: gene ~ Fam1 + Fam2 + Mare + Age + snp * Treat * RNAseq_condition 
##                                Value  Std.Error DF  t-value p-value
## (Intercept)                 8.132507 0.20945634 74 38.82674  0.0000
## Fam1                       -0.324861 0.09212063 74 -3.52648  0.0007
## Fam2                       -0.373140 0.09662494 74 -3.86174  0.0002
## Mare                       -0.021977 0.07388120 74 -0.29746  0.7669
## Age                        -0.004364 0.01024238 74 -0.42612  0.6713
## snp                         0.012755 0.08159070 74  0.15633  0.8762
## Treat                      -0.059639 0.08880925 69 -0.67154  0.5041
## RNAseq_condition           -0.144310 0.13415458 74 -1.07570  0.2856
## snp:Treat                   0.003871 0.07142958 69  0.05419  0.9569
## snp:RNAseq_condition       -0.056875 0.11414465 74 -0.49827  0.6198
## Treat:RNAseq_condition     -0.015931 0.11930960 69 -0.13353  0.8942
## snp:Treat:RNAseq_condition  0.092350 0.10273530 69  0.89892  0.3718
##  Correlation: 
##                            (Intr) Fam1   Fam2   Mare   Age    snp   
## Fam1                       -0.113                                   
## Fam2                       -0.377  0.284                            
## Mare                       -0.044 -0.073 -0.056                     
## Age                        -0.839 -0.071  0.204 -0.119              
## snp                        -0.387  0.225  0.229 -0.046 -0.056       
## Treat                      -0.230 -0.001  0.022 -0.010 -0.015  0.425
## RNAseq_condition           -0.445 -0.009  0.126  0.199  0.071  0.588
## snp:Treat                   0.199  0.004 -0.017  0.013  0.005 -0.494
## snp:RNAseq_condition        0.305 -0.023 -0.071 -0.164  0.002 -0.664
## Treat:RNAseq_condition      0.190 -0.025 -0.039  0.009 -0.003 -0.324
## snp:Treat:RNAseq_condition -0.152  0.012  0.022 -0.019  0.011  0.347
##                            Treat  RNAsq_ snp:Tr s:RNA_ T:RNA_
## Fam1                                                         
## Fam2                                                         
## Mare                                                         
## Age                                                          
## snp                                                          
## Treat                                                        
## RNAseq_condition            0.374                            
## snp:Treat                  -0.841 -0.316                     
## snp:RNAseq_condition       -0.299 -0.797  0.349              
## Treat:RNAseq_condition     -0.744 -0.509  0.626  0.408       
## snp:Treat:RNAseq_condition  0.585  0.401 -0.695 -0.494 -0.795
## 
## Standardized Within-Group Residuals:
##        Min         Q1        Med         Q3        Max 
## -2.2979689 -0.5024235 -0.0320058  0.5040562  2.1141252 
## 
## Number of Observations: 155
## Number of Groups: 82 
## [1] "Fixed Effect Regression Estimates of Mixed Model with REML"
```

```
## [1] "Added Variable aka. Partial Regression Plots: for multiple linear regression (no random effects)"
```

```
## 'data.frame':    155 obs. of  9 variables:
##  $ gene            : num  8.52 8.31 8.42 8.44 8.2 ...
##  $ snp             : num  0 1 1 1 1 0 0 1 1 1 ...
##  $ Fam1            : num  1 0 0 0 1 1 1 1 1 0 ...
##  $ Fam2            : num  0 1 1 1 0 0 0 0 0 1 ...
##  $ Mare            : num  1 1 1 0 0 0 0 1 1 0 ...
##  $ Age             : num  18 18 13 18 19 17 20 19 15 15 ...
##  $ RNAseq_condition: num  0 1 0 0 1 1 1 1 0 0 ...
##  $ Treat           : num  0 0 0 0 0 0 0 0 0 0 ...
##  $ ID              : chr  "26" "29" "30" "31" ...
## 'data.frame':    155 obs. of  9 variables:
##  $ gene            : num  8.52 8.31 8.42 8.44 8.2 ...
##  $ snp             : num  0 1 1 1 1 0 0 1 1 1 ...
##  $ Fam1            : num  1 0 0 0 1 1 1 1 1 0 ...
##  $ Fam2            : num  0 1 1 1 0 0 0 0 0 1 ...
##  $ Mare            : num  1 1 1 0 0 0 0 1 1 0 ...
##  $ Age             : num  18 18 13 18 19 17 20 19 15 15 ...
##  $ RNAseq_condition: num  0 1 0 0 1 1 1 1 0 0 ...
##  $ Treat           : num  0 0 0 0 0 0 0 0 0 0 ...
##  $ ID              : Factor w/ 82 levels "100","110","111",..: 5 6 7 8 10 12 13 14 16 17 ...
## [1] "chr13.32844446"
## [1] "DEXI"
## Linear mixed model fit by REML ['lmerMod']
## Formula: 
## gene ~ Fam1 + Fam2 + Mare + Age + snp * Treat * RNAseq_condition +  
##     (1 | ID)
##    Data: comb.df
## 
## REML criterion at convergence: -78.8
## 
## Scaled residuals: 
##      Min       1Q   Median       3Q      Max 
## -2.06056 -0.63654  0.06842  0.54524  2.11149 
## 
## Random effects:
##  Groups   Name        Variance Std.Dev.
##  ID       (Intercept) 0.007423 0.08616 
##  Residual             0.018736 0.13688 
## Number of obs: 155, groups:  ID, 82
## 
## Fixed effects:
##                             Estimate Std. Error t value
## (Intercept)                 8.240480   0.091153   90.40
## Fam1                        0.172040   0.040515    4.25
## Fam2                        0.142903   0.042054    3.40
## Mare                        0.006712   0.032049    0.21
## Age                        -0.005394   0.004488   -1.20
## snp                        -0.128019   0.041624   -3.08
## Treat                      -0.167421   0.057521   -2.91
## RNAseq_condition           -0.082477   0.066615   -1.24
## snp:Treat                   0.168634   0.045948    3.67
## snp:RNAseq_condition        0.052714   0.056251    0.94
## Treat:RNAseq_condition      0.120926   0.075938    1.59
## snp:Treat:RNAseq_condition -0.115131   0.065174   -1.77
## 
## Correlation of Fixed Effects:
##             (Intr) Fam1   Fam2   Mare   Age    snp    Treat  RNAsq_ snp:Tr
## Fam1        -0.106                                                        
## Fam2        -0.363  0.289                                                 
## Mare        -0.038 -0.072 -0.055                                          
## Age         -0.785 -0.105  0.164 -0.112                                   
## snp         -0.385  0.231  0.225 -0.063 -0.151                            
## Treat       -0.334  0.004  0.031 -0.014 -0.030  0.540                     
## RNAsq_cndtn -0.456  0.032  0.144  0.158  0.000  0.621  0.484              
## snp:Treat    0.290  0.000 -0.024  0.017  0.017 -0.619 -0.852 -0.413       
## snp:RNAsq_c  0.315 -0.054 -0.087 -0.121  0.064 -0.687 -0.393 -0.808  0.453
## Trt:RNAsq_c  0.274 -0.034 -0.049  0.013  0.007 -0.417 -0.758 -0.643  0.645
## snp:Tr:RNA_ -0.220  0.017  0.029 -0.024  0.004  0.439  0.600  0.510 -0.705
##             s:RNA_ T:RNA_
## Fam1                     
## Fam2                     
## Mare                     
## Age                      
## snp                      
## Treat                    
## RNAsq_cndtn              
## snp:Treat                
## snp:RNAsq_c              
## Trt:RNAsq_c  0.522       
## snp:Tr:RNA_ -0.629 -0.801
## Linear mixed-effects model fit by maximum likelihood
##  Data: comb.df 
##         AIC       BIC   logLik
##   -115.5515 -72.94352 71.77573
## 
## Random effects:
##  Formula: ~1 | ID
##         (Intercept)  Residual
## StdDev:  0.07838852 0.1334229
## 
## Fixed effects: gene ~ Fam1 + Fam2 + Mare + Age + snp * Treat * RNAseq_condition 
##                                Value  Std.Error DF  t-value p-value
## (Intercept)                 8.240842 0.09013720 74 91.42553  0.0000
## Fam1                        0.171533 0.03997346 74  4.29117  0.0001
## Fam2                        0.142748 0.04148467 74  3.44098  0.0010
## Mare                        0.006494 0.03160783 74  0.20546  0.8378
## Age                        -0.005355 0.00442549 74 -1.20992  0.2302
## snp                        -0.128647 0.04147503 74 -3.10178  0.0027
## Treat                      -0.168096 0.05830156 69 -2.88322  0.0052
## RNAseq_condition           -0.083551 0.06642389 74 -1.25785  0.2124
## snp:Treat                   0.169108 0.04657628 69  3.63077  0.0005
## snp:RNAseq_condition        0.053481 0.05606985 74  0.95383  0.3433
## Treat:RNAseq_condition      0.121919 0.07696603 69  1.58406  0.1178
## snp:Treat:RNAseq_condition -0.115784 0.06607857 69 -1.75222  0.0842
##  Correlation: 
##                            (Intr) Fam1   Fam2   Mare   Age    snp   
## Fam1                       -0.106                                   
## Fam2                       -0.363  0.289                            
## Mare                       -0.037 -0.071 -0.055                     
## Age                        -0.782 -0.105  0.164 -0.112              
## snp                        -0.390  0.228  0.223 -0.063 -0.151       
## Treat                      -0.342  0.004  0.031 -0.014 -0.030  0.548
## RNAseq_condition           -0.459  0.033  0.145  0.155  0.000  0.622
## snp:Treat                   0.297  0.000 -0.025  0.018  0.018 -0.629
## snp:RNAseq_condition        0.319 -0.054 -0.087 -0.119  0.063 -0.688
## Treat:RNAseq_condition      0.280 -0.034 -0.050  0.013  0.007 -0.423
## snp:Treat:RNAseq_condition -0.225  0.017  0.029 -0.024  0.004  0.446
##                            Treat  RNAsq_ snp:Tr s:RNA_ T:RNA_
## Fam1                                                         
## Fam2                                                         
## Mare                                                         
## Age                                                          
## snp                                                          
## Treat                                                        
## RNAseq_condition            0.491                            
## snp:Treat                  -0.852 -0.419                     
## snp:RNAseq_condition       -0.399 -0.808  0.460              
## Treat:RNAseq_condition     -0.758 -0.652  0.645  0.530       
## snp:Treat:RNAseq_condition  0.600  0.517 -0.705 -0.639 -0.800
## 
## Standardized Within-Group Residuals:
##         Min          Q1         Med          Q3         Max 
## -2.19674300 -0.66146680  0.09445234  0.56402186  2.24970959 
## 
## Number of Observations: 155
## Number of Groups: 82 
## [1] "Fixed Effect Regression Estimates of Mixed Model with REML"
```

```
## [1] "Added Variable aka. Partial Regression Plots: for multiple linear regression (no random effects)"
```

```
## 'data.frame':    155 obs. of  9 variables:
##  $ gene            : num  10.01 9.99 10.15 10 9.99 ...
##  $ snp             : num  0 1 1 1 1 0 0 1 1 1 ...
##  $ Fam1            : num  1 0 0 0 1 1 1 1 1 0 ...
##  $ Fam2            : num  0 1 1 1 0 0 0 0 0 1 ...
##  $ Mare            : num  1 1 1 0 0 0 0 1 1 0 ...
##  $ Age             : num  18 18 13 18 19 17 20 19 15 15 ...
##  $ RNAseq_condition: num  0 1 0 0 1 1 1 1 0 0 ...
##  $ Treat           : num  0 0 0 0 0 0 0 0 0 0 ...
##  $ ID              : chr  "26" "29" "30" "31" ...
## 'data.frame':    155 obs. of  9 variables:
##  $ gene            : num  10.01 9.99 10.15 10 9.99 ...
##  $ snp             : num  0 1 1 1 1 0 0 1 1 1 ...
##  $ Fam1            : num  1 0 0 0 1 1 1 1 1 0 ...
##  $ Fam2            : num  0 1 1 1 0 0 0 0 0 1 ...
##  $ Mare            : num  1 1 1 0 0 0 0 1 1 0 ...
##  $ Age             : num  18 18 13 18 19 17 20 19 15 15 ...
##  $ RNAseq_condition: num  0 1 0 0 1 1 1 1 0 0 ...
##  $ Treat           : num  0 0 0 0 0 0 0 0 0 0 ...
##  $ ID              : Factor w/ 82 levels "100","110","111",..: 5 6 7 8 10 12 13 14 16 17 ...
## [1] "chr13.32844446"
## [1] "NSUN2"
## Linear mixed model fit by REML ['lmerMod']
## Formula: 
## gene ~ Fam1 + Fam2 + Mare + Age + snp * Treat * RNAseq_condition +  
##     (1 | ID)
##    Data: comb.df
## 
## REML criterion at convergence: -215.2
## 
## Scaled residuals: 
##      Min       1Q   Median       3Q      Max 
## -1.77476 -0.48774  0.02677  0.51213  1.75796 
## 
## Random effects:
##  Groups   Name        Variance Std.Dev.
##  ID       (Intercept) 0.006476 0.08048 
##  Residual             0.005153 0.07178 
## Number of obs: 155, groups:  ID, 82
## 
## Fixed effects:
##                             Estimate Std. Error t value
## (Intercept)                10.144204   0.064953  156.18
## Fam1                       -0.041529   0.029414   -1.41
## Fam2                       -0.079965   0.030584   -2.61
## Mare                        0.001771   0.023360    0.08
## Age                        -0.002768   0.003279   -0.84
## snp                        -0.017101   0.027472   -0.62
## Treat                       0.134572   0.030532    4.41
## RNAseq_condition           -0.025362   0.043625   -0.58
## snp:Treat                   0.027916   0.024363    1.15
## snp:RNAseq_condition        0.011689   0.037053    0.32
## Treat:RNAseq_condition      0.019404   0.040316    0.48
## snp:Treat:RNAseq_condition -0.042533   0.034490   -1.23
## 
## Correlation of Fixed Effects:
##             (Intr) Fam1   Fam2   Mare   Age    snp    Treat  RNAsq_ snp:Tr
## Fam1        -0.105                                                        
## Fam2        -0.368  0.292                                                 
## Mare        -0.042 -0.079 -0.060                                          
## Age         -0.814 -0.106  0.170 -0.109                                   
## snp         -0.339  0.255  0.236 -0.064 -0.159                            
## Treat       -0.251  0.003  0.026 -0.012 -0.025  0.445                     
## RNAsq_cndtn -0.426  0.019  0.135  0.181  0.005  0.608  0.400              
## snp:Treat    0.219  0.000 -0.021  0.015  0.015 -0.507 -0.854 -0.342       
## snp:RNAsq_c  0.280 -0.050 -0.080 -0.145  0.072 -0.678 -0.324 -0.805  0.371
## Trt:RNAsq_c  0.208 -0.029 -0.042  0.011  0.006 -0.344 -0.757 -0.531  0.647
## snp:Tr:RNA_ -0.169  0.014  0.025 -0.021  0.003  0.361  0.603  0.422 -0.706
##             s:RNA_ T:RNA_
## Fam1                     
## Fam2                     
## Mare                     
## Age                      
## snp                      
## Treat                    
## RNAsq_cndtn              
## snp:Treat                
## snp:RNAsq_c              
## Trt:RNAsq_c  0.429       
## snp:Tr:RNA_ -0.513 -0.803
## Linear mixed-effects model fit by maximum likelihood
##  Data: comb.df 
##         AIC       BIC   logLik
##   -262.1847 -219.5768 145.0924
## 
## Random effects:
##  Formula: ~1 | ID
##         (Intercept)   Residual
## StdDev:  0.07529624 0.06997492
## 
## Fixed effects: gene ~ Fam1 + Fam2 + Mare + Age + snp * Treat * RNAseq_condition 
##                                Value  Std.Error DF   t-value p-value
## (Intercept)                10.144606 0.06416534 74 158.10102  0.0000
## Fam1                       -0.041632 0.02902285 74  -1.43446  0.1557
## Fam2                       -0.080002 0.03017343 74  -2.65139  0.0098
## Mare                        0.001684 0.02304260 74   0.07307  0.9420
## Age                        -0.002772 0.00323378 74  -0.85720  0.3941
## snp                        -0.017494 0.02729550 74  -0.64090  0.5236
## Treat                       0.134321 0.03095930 69   4.33862  0.0000
## RNAseq_condition           -0.025752 0.04337210 74  -0.59375  0.5545
## snp:Treat                   0.028295 0.02470630 69   1.14524  0.2561
## snp:RNAseq_condition        0.012079 0.03681684 74   0.32808  0.7438
## Treat:RNAseq_condition      0.019759 0.04087946 69   0.48335  0.6304
## snp:Treat:RNAseq_condition -0.042897 0.03498069 69  -1.22631  0.2242
##  Correlation: 
##                            (Intr) Fam1   Fam2   Mare   Age    snp   
## Fam1                       -0.105                                   
## Fam2                       -0.367  0.292                            
## Mare                       -0.042 -0.078 -0.060                     
## Age                        -0.812 -0.105  0.169 -0.109              
## snp                        -0.342  0.253  0.235 -0.064 -0.158       
## Treat                      -0.257  0.003  0.027 -0.013 -0.026  0.453
## RNAseq_condition           -0.428  0.020  0.136  0.179  0.005  0.609
## snp:Treat                   0.225  0.000 -0.021  0.015  0.015 -0.517
## snp:RNAseq_condition        0.282 -0.051 -0.081 -0.144  0.071 -0.679
## Treat:RNAseq_condition      0.214 -0.029 -0.043  0.011  0.006 -0.350
## snp:Treat:RNAseq_condition -0.173  0.015  0.025 -0.021  0.003  0.368
##                            Treat  RNAsq_ snp:Tr s:RNA_ T:RNA_
## Fam1                                                         
## Fam2                                                         
## Mare                                                         
## Age                                                          
## snp                                                          
## Treat                                                        
## RNAseq_condition            0.407                            
## snp:Treat                  -0.854 -0.348                     
## snp:RNAseq_condition       -0.330 -0.805  0.378              
## Treat:RNAseq_condition     -0.757 -0.541  0.646  0.437       
## snp:Treat:RNAseq_condition  0.603  0.430 -0.706 -0.523 -0.803
## 
## Standardized Within-Group Residuals:
##         Min          Q1         Med          Q3         Max 
## -1.82948061 -0.50946890  0.03685885  0.52026232  1.85992631 
## 
## Number of Observations: 155
## Number of Groups: 82 
## [1] "Fixed Effect Regression Estimates of Mixed Model with REML"
```

```
## [1] "Added Variable aka. Partial Regression Plots: for multiple linear regression (no random effects)"
```

```
## 'data.frame':    155 obs. of  9 variables:
##  $ gene            : num  7.27 6.57 6.05 6.37 6.02 ...
##  $ snp             : num  0 1 1 1 1 0 0 1 1 1 ...
##  $ Fam1            : num  1 0 0 0 1 1 1 1 1 0 ...
##  $ Fam2            : num  0 1 1 1 0 0 0 0 0 1 ...
##  $ Mare            : num  1 1 1 0 0 0 0 1 1 0 ...
##  $ Age             : num  18 18 13 18 19 17 20 19 15 15 ...
##  $ RNAseq_condition: num  0 1 0 0 1 1 1 1 0 0 ...
##  $ Treat           : num  0 0 0 0 0 0 0 0 0 0 ...
##  $ ID              : chr  "26" "29" "30" "31" ...
## 'data.frame':    155 obs. of  9 variables:
##  $ gene            : num  7.27 6.57 6.05 6.37 6.02 ...
##  $ snp             : num  0 1 1 1 1 0 0 1 1 1 ...
##  $ Fam1            : num  1 0 0 0 1 1 1 1 1 0 ...
##  $ Fam2            : num  0 1 1 1 0 0 0 0 0 1 ...
##  $ Mare            : num  1 1 1 0 0 0 0 1 1 0 ...
##  $ Age             : num  18 18 13 18 19 17 20 19 15 15 ...
##  $ RNAseq_condition: num  0 1 0 0 1 1 1 1 0 0 ...
##  $ Treat           : num  0 0 0 0 0 0 0 0 0 0 ...
##  $ ID              : Factor w/ 82 levels "100","110","111",..: 5 6 7 8 10 12 13 14 16 17 ...
## [1] "chr13.32844446"
## [1] "ATF7IP2"
## Linear mixed model fit by REML ['lmerMod']
## Formula: 
## gene ~ Fam1 + Fam2 + Mare + Age + snp * Treat * RNAseq_condition +  
##     (1 | ID)
##    Data: comb.df
## 
## REML criterion at convergence: 270.3
## 
## Scaled residuals: 
##     Min      1Q  Median      3Q     Max 
## -2.8552 -0.4467 -0.1023  0.4132  2.3997 
## 
## Random effects:
##  Groups   Name        Variance Std.Dev.
##  ID       (Intercept) 0.37222  0.6101  
##  Residual             0.09714  0.3117  
## Number of obs: 155, groups:  ID, 82
## 
## Fixed effects:
##                            Estimate Std. Error t value
## (Intercept)                 6.13087    0.43343  14.145
## Fam1                       -0.07060    0.19864  -0.355
## Fam2                        0.31238    0.20683   1.510
## Mare                       -0.08537    0.15827  -0.539
## Age                        -0.01722    0.02226  -0.774
## snp                         0.53191    0.17124   3.106
## Treat                       0.61763    0.13391   4.612
## RNAseq_condition            0.66942    0.26971   2.482
## snp:Treat                  -0.22763    0.10677  -2.132
## snp:RNAseq_condition       -0.48061    0.23107  -2.080
## Treat:RNAseq_condition     -0.35355    0.17684  -1.999
## snp:Treat:RNAseq_condition  0.33856    0.15090   2.244
## 
## Correlation of Fixed Effects:
##             (Intr) Fam1   Fam2   Mare   Age    snp    Treat  RNAsq_ snp:Tr
## Fam1        -0.104                                                        
## Fam2        -0.370  0.294                                                 
## Mare        -0.046 -0.085 -0.064                                          
## Age         -0.835 -0.106  0.174 -0.106                                   
## snp         -0.300  0.277  0.245 -0.064 -0.165                            
## Treat       -0.166  0.003  0.019 -0.009 -0.018  0.319                     
## RNAsq_cndtn -0.401  0.004  0.124  0.204  0.010  0.592  0.288              
## snp:Treat    0.146  0.000 -0.015  0.011  0.011 -0.362 -0.855 -0.247       
## snp:RNAsq_c  0.248 -0.046 -0.073 -0.169  0.079 -0.668 -0.232 -0.799  0.264
## Trt:RNAsq_c  0.139 -0.021 -0.030  0.008  0.004 -0.247 -0.757 -0.383  0.648
## snp:Tr:RNA_ -0.113  0.011  0.018 -0.015  0.002  0.258  0.605  0.305 -0.708
##             s:RNA_ T:RNA_
## Fam1                     
## Fam2                     
## Mare                     
## Age                      
## snp                      
## Treat                    
## RNAsq_cndtn              
## snp:Treat                
## snp:RNAsq_c              
## Trt:RNAsq_c  0.306       
## snp:Tr:RNA_ -0.364 -0.805
## Linear mixed-effects model fit by maximum likelihood
##  Data: comb.df 
##        AIC      BIC    logLik
##   265.7293 308.3373 -118.8647
## 
## Random effects:
##  Formula: ~1 | ID
##         (Intercept)  Residual
## StdDev:   0.5774635 0.3030654
## 
## Fixed effects: gene ~ Fam1 + Fam2 + Mare + Age + snp * Treat * RNAseq_condition 
##                                Value Std.Error DF   t-value p-value
## (Intercept)                 6.131030 0.4288012 74 14.298072  0.0000
## Fam1                       -0.070502 0.1964383 74 -0.358903  0.7207
## Fam2                        0.312327 0.2045324 74  1.527032  0.1310
## Mare                       -0.085414 0.1565025 74 -0.545765  0.5869
## Age                        -0.017222 0.0220072 74 -0.782558  0.4364
## snp                         0.532177 0.1698621 74  3.132995  0.0025
## Treat                       0.617479 0.1355120 69  4.556637  0.0000
## RNAseq_condition            0.669062 0.2676329 74  2.499923  0.0146
## snp:Treat                  -0.227886 0.1080505 69 -2.109066  0.0386
## snp:RNAseq_condition       -0.480530 0.2291973 74 -2.096580  0.0394
## Treat:RNAseq_condition     -0.353223 0.1789607 69 -1.973747  0.0524
## snp:Treat:RNAseq_condition  0.338498 0.1527232 69  2.216415  0.0300
##  Correlation: 
##                            (Intr) Fam1   Fam2   Mare   Age    snp   
## Fam1                       -0.104                                   
## Fam2                       -0.370  0.294                            
## Mare                       -0.046 -0.084 -0.064                     
## Age                        -0.834 -0.106  0.174 -0.107              
## snp                        -0.301  0.276  0.245 -0.064 -0.164       
## Treat                      -0.170  0.003  0.019 -0.009 -0.018  0.325
## RNAseq_condition           -0.402  0.005  0.125  0.203  0.010  0.593
## snp:Treat                   0.149  0.000 -0.015  0.011  0.011 -0.369
## snp:RNAseq_condition        0.249 -0.046 -0.073 -0.168  0.079 -0.668
## Treat:RNAseq_condition      0.142 -0.021 -0.031  0.008  0.004 -0.252
## snp:Treat:RNAseq_condition -0.116  0.011  0.018 -0.015  0.002  0.263
##                            Treat  RNAsq_ snp:Tr s:RNA_ T:RNA_
## Fam1                                                         
## Fam2                                                         
## Mare                                                         
## Age                                                          
## snp                                                          
## Treat                                                        
## RNAseq_condition            0.293                            
## snp:Treat                  -0.855 -0.252                     
## snp:RNAseq_condition       -0.236 -0.800  0.270              
## Treat:RNAseq_condition     -0.757 -0.390  0.648  0.312       
## snp:Treat:RNAseq_condition  0.605  0.310 -0.707 -0.372 -0.805
## 
## Standardized Within-Group Residuals:
##        Min         Q1        Med         Q3        Max 
## -2.9483994 -0.4736892 -0.1020008  0.4331361  2.4569697 
## 
## Number of Observations: 155
## Number of Groups: 82 
## [1] "Fixed Effect Regression Estimates of Mixed Model with REML"
```

```
## [1] "Added Variable aka. Partial Regression Plots: for multiple linear regression (no random effects)"
```

```
## 'data.frame':    155 obs. of  9 variables:
##  $ gene            : num  7.63 7.92 7.58 7.29 8 ...
##  $ snp             : num  0 1 1 1 1 0 0 1 1 1 ...
##  $ Fam1            : num  1 0 0 0 1 1 1 1 1 0 ...
##  $ Fam2            : num  0 1 1 1 0 0 0 0 0 1 ...
##  $ Mare            : num  1 1 1 0 0 0 0 1 1 0 ...
##  $ Age             : num  18 18 13 18 19 17 20 19 15 15 ...
##  $ RNAseq_condition: num  0 1 0 0 1 1 1 1 0 0 ...
##  $ Treat           : num  0 0 0 0 0 0 0 0 0 0 ...
##  $ ID              : chr  "26" "29" "30" "31" ...
## 'data.frame':    155 obs. of  9 variables:
##  $ gene            : num  7.63 7.92 7.58 7.29 8 ...
##  $ snp             : num  0 1 1 1 1 0 0 1 1 1 ...
##  $ Fam1            : num  1 0 0 0 1 1 1 1 1 0 ...
##  $ Fam2            : num  0 1 1 1 0 0 0 0 0 1 ...
##  $ Mare            : num  1 1 1 0 0 0 0 1 1 0 ...
##  $ Age             : num  18 18 13 18 19 17 20 19 15 15 ...
##  $ RNAseq_condition: num  0 1 0 0 1 1 1 1 0 0 ...
##  $ Treat           : num  0 0 0 0 0 0 0 0 0 0 ...
##  $ ID              : Factor w/ 82 levels "100","110","111",..: 5 6 7 8 10 12 13 14 16 17 ...
## [1] "chr13.32844446"
## [1] "GLIPR1L2"
## Linear mixed model fit by REML ['lmerMod']
## Formula: 
## gene ~ Fam1 + Fam2 + Mare + Age + snp * Treat * RNAseq_condition +  
##     (1 | ID)
##    Data: comb.df
## 
## REML criterion at convergence: 107.9
## 
## Scaled residuals: 
##      Min       1Q   Median       3Q      Max 
## -2.17163 -0.48334 -0.03442  0.47470  2.03206 
## 
## Random effects:
##  Groups   Name        Variance Std.Dev.
##  ID       (Intercept) 0.07019  0.2649  
##  Residual             0.04590  0.2142  
## Number of obs: 155, groups:  ID, 82
## 
## Fixed effects:
##                             Estimate Std. Error t value
## (Intercept)                 8.154194   0.207380   39.32
## Fam1                       -0.333222   0.094163   -3.54
## Fam2                       -0.381228   0.097937   -3.89
## Mare                       -0.020788   0.074832   -0.28
## Age                        -0.003975   0.010508   -0.38
## snp                        -0.011740   0.086533   -0.14
## Treat                      -0.062644   0.091309   -0.69
## RNAseq_condition           -0.167837   0.137202   -1.22
## snp:Treat                   0.005770   0.072850    0.08
## snp:RNAseq_condition       -0.034712   0.116703   -0.30
## Treat:RNAseq_condition     -0.012638   0.120573   -0.10
## snp:Treat:RNAseq_condition  0.090332   0.103096    0.88
## 
## Correlation of Fixed Effects:
##             (Intr) Fam1   Fam2   Mare   Age    snp    Treat  RNAsq_ snp:Tr
## Fam1        -0.105                                                        
## Fam2        -0.368  0.292                                                 
## Mare        -0.043 -0.080 -0.061                                          
## Age         -0.818 -0.106  0.171 -0.108                                   
## snp         -0.331  0.259  0.238 -0.064 -0.160                            
## Treat       -0.235  0.003  0.025 -0.012 -0.024  0.424                     
## RNAsq_cndtn -0.421  0.016  0.133  0.185  0.006  0.605  0.381              
## snp:Treat    0.206  0.000 -0.020  0.014  0.014 -0.483 -0.854 -0.327       
## snp:RNAsq_c  0.273 -0.050 -0.079 -0.150  0.073 -0.676 -0.308 -0.804  0.353
## Trt:RNAsq_c  0.196 -0.027 -0.040  0.011  0.006 -0.328 -0.757 -0.507  0.647
## snp:Tr:RNA_ -0.159  0.014  0.024 -0.020  0.003  0.344  0.603  0.403 -0.707
##             s:RNA_ T:RNA_
## Fam1                     
## Fam2                     
## Mare                     
## Age                      
## snp                      
## Treat                    
## RNAsq_cndtn              
## snp:Treat                
## snp:RNAsq_c              
## Trt:RNAsq_c  0.409       
## snp:Tr:RNA_ -0.488 -0.803
## Linear mixed-effects model fit by maximum likelihood
##  Data: comb.df 
##        AIC      BIC    logLik
##   88.33232 130.9403 -30.16616
## 
## Random effects:
##  Formula: ~1 | ID
##         (Intercept)  Residual
## StdDev:   0.2492401 0.2084317
## 
## Fixed effects: gene ~ Fam1 + Fam2 + Mare + Age + snp * Treat * RNAseq_condition 
##                                Value  Std.Error DF  t-value p-value
## (Intercept)                 8.154918 0.20516790 74 39.74753  0.0000
## Fam1                       -0.333131 0.09307549 74 -3.57915  0.0006
## Fam2                       -0.381305 0.09679628 74 -3.93925  0.0002
## Mare                       -0.020670 0.07395141 74 -0.27951  0.7806
## Age                        -0.004013 0.01038268 74 -0.38652  0.7002
## snp                        -0.012334 0.08600457 74 -0.14342  0.8864
## Treat                      -0.062791 0.09242489 69 -0.67938  0.4992
## RNAseq_condition           -0.167996 0.13643608 74 -1.23132  0.2221
## snp:Treat                   0.006413 0.07374371 69  0.08697  0.9309
## snp:RNAseq_condition       -0.034122 0.11599201 74 -0.29418  0.7694
## Treat:RNAseq_condition     -0.012456 0.12204449 69 -0.10206  0.9190
## snp:Treat:RNAseq_condition  0.089687 0.10437290 69  0.85930  0.3932
##  Correlation: 
##                            (Intr) Fam1   Fam2   Mare   Age    snp   
## Fam1                       -0.105                                   
## Fam2                       -0.368  0.292                            
## Mare                       -0.043 -0.080 -0.061                     
## Age                        -0.817 -0.106  0.170 -0.108              
## snp                        -0.334  0.258  0.237 -0.064 -0.159       
## Treat                      -0.241  0.003  0.025 -0.012 -0.024  0.431
## RNAseq_condition           -0.423  0.017  0.134  0.184  0.006  0.606
## snp:Treat                   0.211  0.000 -0.020  0.015  0.014 -0.491
## snp:RNAseq_condition        0.276 -0.050 -0.080 -0.148  0.073 -0.677
## Treat:RNAseq_condition      0.200 -0.028 -0.041  0.011  0.006 -0.333
## snp:Treat:RNAseq_condition -0.162  0.014  0.024 -0.020  0.003  0.350
##                            Treat  RNAsq_ snp:Tr s:RNA_ T:RNA_
## Fam1                                                         
## Fam2                                                         
## Mare                                                         
## Age                                                          
## snp                                                          
## Treat                                                        
## RNAseq_condition            0.388                            
## snp:Treat                  -0.854 -0.332                     
## snp:RNAseq_condition       -0.314 -0.804  0.359              
## Treat:RNAseq_condition     -0.757 -0.515  0.647  0.416       
## snp:Treat:RNAseq_condition  0.603  0.409 -0.707 -0.497 -0.803
## 
## Standardized Within-Group Residuals:
##         Min          Q1         Med          Q3         Max 
## -2.26792547 -0.49570441 -0.03443454  0.49892514  2.10947370 
## 
## Number of Observations: 155
## Number of Groups: 82 
## [1] "Fixed Effect Regression Estimates of Mixed Model with REML"
```

```
## [1] "Added Variable aka. Partial Regression Plots: for multiple linear regression (no random effects)"
```

```
## 'data.frame':    155 obs. of  9 variables:
##  $ gene            : num  8.52 8.31 8.42 8.44 8.2 ...
##  $ snp             : num  0 2 1 1 0 0 0 0 0 1 ...
##  $ Fam1            : num  1 0 0 0 1 1 1 1 1 0 ...
##  $ Fam2            : num  0 1 1 1 0 0 0 0 0 1 ...
##  $ Mare            : num  1 1 1 0 0 0 0 1 1 0 ...
##  $ Age             : num  18 18 13 18 19 17 20 19 15 15 ...
##  $ RNAseq_condition: num  0 1 0 0 1 1 1 1 0 0 ...
##  $ Treat           : num  0 0 0 0 0 0 0 0 0 0 ...
##  $ ID              : chr  "26" "29" "30" "31" ...
## 'data.frame':    155 obs. of  9 variables:
##  $ gene            : num  8.52 8.31 8.42 8.44 8.2 ...
##  $ snp             : num  0 2 1 1 0 0 0 0 0 1 ...
##  $ Fam1            : num  1 0 0 0 1 1 1 1 1 0 ...
##  $ Fam2            : num  0 1 1 1 0 0 0 0 0 1 ...
##  $ Mare            : num  1 1 1 0 0 0 0 1 1 0 ...
##  $ Age             : num  18 18 13 18 19 17 20 19 15 15 ...
##  $ RNAseq_condition: num  0 1 0 0 1 1 1 1 0 0 ...
##  $ Treat           : num  0 0 0 0 0 0 0 0 0 0 ...
##  $ ID              : Factor w/ 82 levels "100","110","111",..: 5 6 7 8 10 12 13 14 16 17 ...
## [1] "chr13.33460982"
## [1] "DEXI"
## Linear mixed model fit by REML ['lmerMod']
## Formula: 
## gene ~ Fam1 + Fam2 + Mare + Age + snp * Treat * RNAseq_condition +  
##     (1 | ID)
##    Data: comb.df
## 
## REML criterion at convergence: -68
## 
## Scaled residuals: 
##     Min      1Q  Median      3Q     Max 
## -2.6066 -0.5490  0.1166  0.5302  2.2128 
## 
## Random effects:
##  Groups   Name        Variance Std.Dev.
##  ID       (Intercept) 0.005729 0.07569 
##  Residual             0.022349 0.14949 
## Number of obs: 155, groups:  ID, 82
## 
## Fixed effects:
##                              Estimate Std. Error t value
## (Intercept)                 8.147e+00  8.573e-02   95.03
## Fam1                        1.978e-01  3.847e-02    5.14
## Fam2                        1.352e-01  4.402e-02    3.07
## Mare                       -2.434e-03  3.104e-02   -0.08
## Age                        -8.080e-03  4.488e-03   -1.80
## snp                         2.906e-02  6.788e-02    0.43
## Treat                       1.261e-02  3.675e-02    0.34
## RNAseq_condition           -3.231e-02  4.522e-02   -0.71
## snp:Treat                  -6.426e-05  8.191e-02    0.00
## snp:RNAseq_condition        4.480e-02  8.052e-02    0.56
## Treat:RNAseq_condition     -7.917e-03  5.471e-02   -0.14
## snp:Treat:RNAseq_condition -1.737e-02  9.999e-02   -0.17
## 
## Correlation of Fixed Effects:
##             (Intr) Fam1   Fam2   Mare   Age    snp    Treat  RNAsq_ snp:Tr
## Fam1        -0.041                                                        
## Fam2        -0.331  0.156                                                 
## Mare        -0.034  0.018 -0.055                                          
## Age         -0.911 -0.062  0.270 -0.166                                   
## snp         -0.108  0.057 -0.200  0.073 -0.049                            
## Treat       -0.208  0.017  0.007  0.015 -0.024  0.285                     
## RNAsq_cndtn -0.368 -0.050  0.127  0.102  0.127  0.262  0.426              
## snp:Treat    0.100 -0.045  0.012 -0.028  0.010 -0.650 -0.449 -0.190       
## snp:RNAsq_c  0.156 -0.017  0.017 -0.008 -0.030 -0.806 -0.240 -0.433  0.542
## Trt:RNAsq_c  0.154 -0.052 -0.035 -0.017  0.009 -0.189 -0.673 -0.648  0.303
## snp:Tr:RNA_ -0.081  0.052 -0.012  0.030 -0.012  0.535  0.368  0.281 -0.820
##             s:RNA_ T:RNA_
## Fam1                     
## Fam2                     
## Mare                     
## Age                      
## snp                      
## Treat                    
## RNAsq_cndtn              
## snp:Treat                
## snp:RNAsq_c              
## Trt:RNAsq_c  0.291       
## snp:Tr:RNA_ -0.655 -0.444
## Linear mixed-effects model fit by maximum likelihood
##  Data: comb.df 
##         AIC       BIC   logLik
##   -101.5646 -58.95669 64.78232
## 
## Random effects:
##  Formula: ~1 | ID
##         (Intercept)  Residual
## StdDev:  0.06719449 0.1457434
## 
## Fixed effects: gene ~ Fam1 + Fam2 + Mare + Age + snp * Treat * RNAseq_condition 
##                                Value  Std.Error DF  t-value p-value
## (Intercept)                 8.146914 0.08458681 74 96.31423  0.0000
## Fam1                        0.197442 0.03795282 74  5.20231  0.0000
## Fam2                        0.135111 0.04341548 74  3.11205  0.0026
## Mare                       -0.002647 0.03060947 74 -0.08648  0.9313
## Age                        -0.008051 0.00442313 74 -1.82015  0.0728
## snp                         0.028575 0.06770543 74  0.42204  0.6742
## Treat                       0.012317 0.03727840 69  0.33041  0.7421
## RNAseq_condition           -0.032953 0.04510329 74 -0.73062  0.4673
## snp:Treat                   0.000409 0.08307644 69  0.00492  0.9961
## snp:RNAseq_condition        0.045754 0.08033145 74  0.56956  0.5707
## Treat:RNAseq_condition     -0.007225 0.05548634 69 -0.13022  0.8968
## snp:Treat:RNAseq_condition -0.018395 0.10142400 69 -0.18137  0.8566
##  Correlation: 
##                            (Intr) Fam1   Fam2   Mare   Age    snp   
## Fam1                       -0.042                                   
## Fam2                       -0.331  0.155                            
## Mare                       -0.034  0.019 -0.055                     
## Age                        -0.910 -0.061  0.269 -0.167              
## snp                        -0.110  0.059 -0.198  0.073 -0.048       
## Treat                      -0.214  0.017  0.008  0.015 -0.025  0.290
## RNAseq_condition           -0.369 -0.048  0.127  0.102  0.125  0.263
## snp:Treat                   0.103 -0.045  0.012 -0.028  0.010 -0.660
## snp:RNAseq_condition        0.157 -0.019  0.017 -0.009 -0.029 -0.806
## Treat:RNAseq_condition      0.158 -0.052 -0.036 -0.018  0.009 -0.192
## snp:Treat:RNAseq_condition -0.083  0.053 -0.012  0.030 -0.012  0.543
##                            Treat  RNAsq_ snp:Tr s:RNA_ T:RNA_
## Fam1                                                         
## Fam2                                                         
## Mare                                                         
## Age                                                          
## snp                                                          
## Treat                                                        
## RNAseq_condition            0.433                            
## snp:Treat                  -0.449 -0.193                     
## snp:RNAseq_condition       -0.243 -0.433  0.551              
## Treat:RNAseq_condition     -0.673 -0.658  0.303  0.296       
## snp:Treat:RNAseq_condition  0.368  0.286 -0.820 -0.666 -0.445
## 
## Standardized Within-Group Residuals:
##        Min         Q1        Med         Q3        Max 
## -2.7477951 -0.5544818  0.1011390  0.5671619  2.3526023 
## 
## Number of Observations: 155
## Number of Groups: 82 
## [1] "Fixed Effect Regression Estimates of Mixed Model with REML"
```

```
## [1] "Added Variable aka. Partial Regression Plots: for multiple linear regression (no random effects)"
```

```
## 'data.frame':    155 obs. of  9 variables:
##  $ gene            : num  10.01 9.99 10.15 10 9.99 ...
##  $ snp             : num  0 2 1 1 0 0 0 0 0 1 ...
##  $ Fam1            : num  1 0 0 0 1 1 1 1 1 0 ...
##  $ Fam2            : num  0 1 1 1 0 0 0 0 0 1 ...
##  $ Mare            : num  1 1 1 0 0 0 0 1 1 0 ...
##  $ Age             : num  18 18 13 18 19 17 20 19 15 15 ...
##  $ RNAseq_condition: num  0 1 0 0 1 1 1 1 0 0 ...
##  $ Treat           : num  0 0 0 0 0 0 0 0 0 0 ...
##  $ ID              : chr  "26" "29" "30" "31" ...
## 'data.frame':    155 obs. of  9 variables:
##  $ gene            : num  10.01 9.99 10.15 10 9.99 ...
##  $ snp             : num  0 2 1 1 0 0 0 0 0 1 ...
##  $ Fam1            : num  1 0 0 0 1 1 1 1 1 0 ...
##  $ Fam2            : num  0 1 1 1 0 0 0 0 0 1 ...
##  $ Mare            : num  1 1 1 0 0 0 0 1 1 0 ...
##  $ Age             : num  18 18 13 18 19 17 20 19 15 15 ...
##  $ RNAseq_condition: num  0 1 0 0 1 1 1 1 0 0 ...
##  $ Treat           : num  0 0 0 0 0 0 0 0 0 0 ...
##  $ ID              : Factor w/ 82 levels "100","110","111",..: 5 6 7 8 10 12 13 14 16 17 ...
## [1] "chr13.33460982"
## [1] "NSUN2"
## Linear mixed model fit by REML ['lmerMod']
## Formula: 
## gene ~ Fam1 + Fam2 + Mare + Age + snp * Treat * RNAseq_condition +  
##     (1 | ID)
##    Data: comb.df
## 
## REML criterion at convergence: -216.7
## 
## Scaled residuals: 
##     Min      1Q  Median      3Q     Max 
## -1.7527 -0.4358 -0.0017  0.5100  1.7298 
## 
## Random effects:
##  Groups   Name        Variance Std.Dev.
##  ID       (Intercept) 0.006573 0.08107 
##  Residual             0.005164 0.07186 
## Number of obs: 155, groups:  ID, 82
## 
## Fixed effects:
##                             Estimate Std. Error t value
## (Intercept)                10.129034   0.062133  163.02
## Fam1                       -0.036236   0.027806   -1.30
## Fam2                       -0.080316   0.031998   -2.51
## Mare                       -0.001479   0.022570   -0.07
## Age                        -0.003048   0.003286   -0.93
## snp                         0.012291   0.043355    0.28
## Treat                       0.166337   0.017801    9.34
## RNAseq_condition           -0.007075   0.028967   -0.24
## snp:Treat                  -0.009589   0.039740   -0.24
## snp:RNAseq_condition       -0.019109   0.051403   -0.37
## Treat:RNAseq_condition     -0.032331   0.026561   -1.22
## snp:Treat:RNAseq_condition  0.035198   0.048438    0.73
## 
## Correlation of Fixed Effects:
##             (Intr) Fam1   Fam2   Mare   Age    snp    Treat  RNAsq_ snp:Tr
## Fam1        -0.034                                                        
## Fam2        -0.335  0.162                                                 
## Mare        -0.035  0.012 -0.058                                          
## Age         -0.927 -0.065  0.273 -0.160                                   
## snp         -0.084  0.035 -0.220  0.066 -0.050                            
## Treat       -0.138  0.014  0.006  0.012 -0.020  0.219                     
## RNAsq_cndtn -0.363 -0.089  0.122  0.107  0.152  0.259  0.326              
## snp:Treat    0.067 -0.037  0.009 -0.023  0.008 -0.502 -0.448 -0.144       
## snp:RNAsq_c  0.147  0.014  0.011  0.009 -0.042 -0.798 -0.184 -0.434  0.418
## Trt:RNAsq_c  0.104 -0.043 -0.029 -0.014  0.007 -0.144 -0.671 -0.499  0.302
## snp:Tr:RNA_ -0.054  0.043 -0.010  0.024 -0.010  0.414  0.368  0.215 -0.821
##             s:RNA_ T:RNA_
## Fam1                     
## Fam2                     
## Mare                     
## Age                      
## snp                      
## Treat                    
## RNAsq_cndtn              
## snp:Treat                
## snp:RNAsq_c              
## Trt:RNAsq_c  0.223       
## snp:Tr:RNA_ -0.504 -0.444
## Linear mixed-effects model fit by maximum likelihood
##  Data: comb.df 
##         AIC       BIC   logLik
##   -261.1265 -218.5185 144.5632
## 
## Random effects:
##  Formula: ~1 | ID
##         (Intercept)   Residual
## StdDev:  0.07587284 0.07004732
## 
## Fixed effects: gene ~ Fam1 + Fam2 + Mare + Age + snp * Treat * RNAseq_condition 
##                                Value  Std.Error DF   t-value p-value
## (Intercept)                10.129056 0.06130218 74 165.23158  0.0000
## Fam1                       -0.036285 0.02744186 74  -1.32225  0.1902
## Fam2                       -0.080309 0.03156948 74  -2.54389  0.0130
## Mare                       -0.001561 0.02226752 74  -0.07011  0.9443
## Age                        -0.003057 0.00324078 74  -0.94334  0.3486
## snp                         0.012515 0.04307031 74   0.29057  0.7722
## Treat                       0.166534 0.01805827 69   9.22206  0.0000
## RNAseq_condition           -0.007033 0.02877117 74  -0.24443  0.8076
## snp:Treat                  -0.009826 0.04031020 69  -0.24376  0.8081
## snp:RNAseq_condition       -0.019350 0.05106204 74  -0.37896  0.7058
## Treat:RNAseq_condition     -0.032391 0.02694122 69  -1.20229  0.2334
## snp:Treat:RNAseq_condition  0.035436 0.04913753 69   0.72117  0.4732
##  Correlation: 
##                            (Intr) Fam1   Fam2   Mare   Age    snp   
## Fam1                       -0.034                                   
## Fam2                       -0.335  0.161                            
## Mare                       -0.035  0.012 -0.058                     
## Age                        -0.926 -0.064  0.273 -0.161              
## snp                        -0.086  0.037 -0.219  0.067 -0.050       
## Treat                      -0.142  0.014  0.006  0.013 -0.021  0.223
## RNAseq_condition           -0.364 -0.087  0.123  0.106  0.150  0.259
## snp:Treat                   0.069 -0.038  0.010 -0.023  0.008 -0.512
## snp:RNAseq_condition        0.148  0.012  0.012  0.008 -0.041 -0.798
## Treat:RNAseq_condition      0.107 -0.044 -0.030 -0.014  0.008 -0.147
## snp:Treat:RNAseq_condition -0.056  0.044 -0.010  0.025 -0.010  0.422
##                            Treat  RNAsq_ snp:Tr s:RNA_ T:RNA_
## Fam1                                                         
## Fam2                                                         
## Mare                                                         
## Age                                                          
## snp                                                          
## Treat                                                        
## RNAseq_condition            0.333                            
## snp:Treat                  -0.448 -0.147                     
## snp:RNAseq_condition       -0.187 -0.434  0.426              
## Treat:RNAseq_condition     -0.671 -0.509  0.302  0.228       
## snp:Treat:RNAseq_condition  0.368  0.219 -0.821 -0.514 -0.444
## 
## Standardized Within-Group Residuals:
##          Min           Q1          Med           Q3          Max 
## -1.840729196 -0.452384720  0.007940998  0.526602485  1.830785100 
## 
## Number of Observations: 155
## Number of Groups: 82 
## [1] "Fixed Effect Regression Estimates of Mixed Model with REML"
```

```
## [1] "Added Variable aka. Partial Regression Plots: for multiple linear regression (no random effects)"
```

```
## 'data.frame':    155 obs. of  9 variables:
##  $ gene            : num  7.27 6.57 6.05 6.37 6.02 ...
##  $ snp             : num  0 2 1 1 0 0 0 0 0 1 ...
##  $ Fam1            : num  1 0 0 0 1 1 1 1 1 0 ...
##  $ Fam2            : num  0 1 1 1 0 0 0 0 0 1 ...
##  $ Mare            : num  1 1 1 0 0 0 0 1 1 0 ...
##  $ Age             : num  18 18 13 18 19 17 20 19 15 15 ...
##  $ RNAseq_condition: num  0 1 0 0 1 1 1 1 0 0 ...
##  $ Treat           : num  0 0 0 0 0 0 0 0 0 0 ...
##  $ ID              : chr  "26" "29" "30" "31" ...
## 'data.frame':    155 obs. of  9 variables:
##  $ gene            : num  7.27 6.57 6.05 6.37 6.02 ...
##  $ snp             : num  0 2 1 1 0 0 0 0 0 1 ...
##  $ Fam1            : num  1 0 0 0 1 1 1 1 1 0 ...
##  $ Fam2            : num  0 1 1 1 0 0 0 0 0 1 ...
##  $ Mare            : num  1 1 1 0 0 0 0 1 1 0 ...
##  $ Age             : num  18 18 13 18 19 17 20 19 15 15 ...
##  $ RNAseq_condition: num  0 1 0 0 1 1 1 1 0 0 ...
##  $ Treat           : num  0 0 0 0 0 0 0 0 0 0 ...
##  $ ID              : Factor w/ 82 levels "100","110","111",..: 5 6 7 8 10 12 13 14 16 17 ...
## [1] "chr13.33460982"
## [1] "ATF7IP2"
## Linear mixed model fit by REML ['lmerMod']
## Formula: 
## gene ~ Fam1 + Fam2 + Mare + Age + snp * Treat * RNAseq_condition +  
##     (1 | ID)
##    Data: comb.df
## 
## REML criterion at convergence: 274.6
## 
## Scaled residuals: 
##     Min      1Q  Median      3Q     Max 
## -2.3217 -0.4430 -0.1332  0.4123  2.0067 
## 
## Random effects:
##  Groups   Name        Variance Std.Dev.
##  ID       (Intercept) 0.40800  0.6387  
##  Residual             0.09807  0.3132  
## Number of obs: 155, groups:  ID, 82
## 
## Fixed effects:
##                             Estimate Std. Error t value
## (Intercept)                 6.563356   0.435604  15.067
## Fam1                       -0.257530   0.194128  -1.327
## Fam2                        0.212849   0.224194   0.949
## Mare                       -0.053141   0.158185  -0.336
## Age                        -0.004738   0.023139  -0.205
## snp                        -0.284699   0.279888  -1.017
## Treat                       0.295737   0.077981   3.792
## RNAseq_condition            0.147333   0.187516   0.786
## snp:Treat                   0.376511   0.174252   2.161
## snp:RNAseq_condition        0.183449   0.332476   0.552
## Treat:RNAseq_condition      0.060526   0.116508   0.519
## snp:Treat:RNAseq_condition -0.379710   0.212167  -1.790
## 
## Correlation of Fixed Effects:
##             (Intr) Fam1   Fam2   Mare   Age    snp    Treat  RNAsq_ snp:Tr
## Fam1        -0.029                                                        
## Fam2        -0.337  0.165                                                 
## Mare        -0.035  0.007 -0.060                                          
## Age         -0.935 -0.066  0.275 -0.157                                   
## snp         -0.071  0.016 -0.233  0.060 -0.049                            
## Treat       -0.086  0.010  0.004  0.009 -0.014  0.150                     
## RNAsq_cndtn -0.364 -0.120  0.116  0.108  0.170  0.259  0.222              
## snp:Treat    0.042 -0.026  0.007 -0.016  0.006 -0.345 -0.448 -0.098       
## snp:RNAsq_c  0.144  0.039  0.006  0.024 -0.051 -0.791 -0.125 -0.437  0.286
## Trt:RNAsq_c  0.065 -0.030 -0.021 -0.010  0.005 -0.097 -0.670 -0.341  0.300
## snp:Tr:RNA_ -0.034  0.030 -0.007  0.017 -0.007  0.284  0.368  0.146 -0.822
##             s:RNA_ T:RNA_
## Fam1                     
## Fam2                     
## Mare                     
## Age                      
## snp                      
## Treat                    
## RNAsq_cndtn              
## snp:Treat                
## snp:RNAsq_c              
## Trt:RNAsq_c  0.152       
## snp:Tr:RNA_ -0.344 -0.443
## Linear mixed-effects model fit by maximum likelihood
##  Data: comb.df 
##        AIC      BIC    logLik
##   273.1403 315.7483 -122.5702
## 
## Random effects:
##  Formula: ~1 | ID
##         (Intercept)  Residual
## StdDev:   0.6047835 0.3045074
## 
## Fixed effects: gene ~ Fam1 + Fam2 + Mare + Age + snp * Treat * RNAseq_condition 
##                                Value Std.Error DF   t-value p-value
## (Intercept)                 6.563632 0.4307599 74 15.237333  0.0000
## Fam1                       -0.257443 0.1920010 74 -1.340843  0.1841
## Fam2                        0.212740 0.2217114 74  0.959538  0.3404
## Mare                       -0.053148 0.1564316 74 -0.339754  0.7350
## Age                        -0.004726 0.0228786 74 -0.206559  0.8369
## snp                        -0.284511 0.2775536 74 -1.025069  0.3087
## Treat                       0.295247 0.0789280 69  3.740714  0.0004
## RNAseq_condition            0.147045 0.1859324 74  0.790854  0.4316
## snp:Treat                   0.376366 0.1763632 69  2.134037  0.0364
## snp:RNAseq_condition        0.182898 0.3296709 74  0.554790  0.5807
## Treat:RNAseq_condition      0.060808 0.1179182 69  0.515678  0.6077
## snp:Treat:RNAseq_condition -0.379161 0.2147453 69 -1.765632  0.0819
##  Correlation: 
##                            (Intr) Fam1   Fam2   Mare   Age    snp   
## Fam1                       -0.029                                   
## Fam2                       -0.337  0.165                            
## Mare                       -0.035  0.008 -0.060                     
## Age                        -0.935 -0.066  0.275 -0.157              
## snp                        -0.071  0.017 -0.232  0.060 -0.049       
## Treat                      -0.088  0.010  0.004  0.009 -0.015  0.153
## RNAseq_condition           -0.364 -0.119  0.116  0.108  0.169  0.259
## snp:Treat                   0.043 -0.026  0.007 -0.016  0.006 -0.352
## snp:RNAseq_condition        0.144  0.038  0.007  0.023 -0.051 -0.791
## Treat:RNAseq_condition      0.067 -0.031 -0.021 -0.010  0.005 -0.099
## snp:Treat:RNAseq_condition -0.035  0.031 -0.007  0.017 -0.007  0.290
##                            Treat  RNAsq_ snp:Tr s:RNA_ T:RNA_
## Fam1                                                         
## Fam2                                                         
## Mare                                                         
## Age                                                          
## snp                                                          
## Treat                                                        
## RNAseq_condition            0.227                            
## snp:Treat                  -0.448 -0.100                     
## snp:RNAseq_condition       -0.128 -0.437  0.292              
## Treat:RNAseq_condition     -0.670 -0.348  0.300  0.155       
## snp:Treat:RNAseq_condition  0.368  0.149 -0.822 -0.351 -0.443
## 
## Standardized Within-Group Residuals:
##        Min         Q1        Med         Q3        Max 
## -2.3967398 -0.4701403 -0.1424894  0.4341493  2.0568828 
## 
## Number of Observations: 155
## Number of Groups: 82 
## [1] "Fixed Effect Regression Estimates of Mixed Model with REML"
```

```
## [1] "Added Variable aka. Partial Regression Plots: for multiple linear regression (no random effects)"
```

```
## 'data.frame':    155 obs. of  9 variables:
##  $ gene            : num  7.63 7.92 7.58 7.29 8 ...
##  $ snp             : num  0 2 1 1 0 0 0 0 0 1 ...
##  $ Fam1            : num  1 0 0 0 1 1 1 1 1 0 ...
##  $ Fam2            : num  0 1 1 1 0 0 0 0 0 1 ...
##  $ Mare            : num  1 1 1 0 0 0 0 1 1 0 ...
##  $ Age             : num  18 18 13 18 19 17 20 19 15 15 ...
##  $ RNAseq_condition: num  0 1 0 0 1 1 1 1 0 0 ...
##  $ Treat           : num  0 0 0 0 0 0 0 0 0 0 ...
##  $ ID              : chr  "26" "29" "30" "31" ...
## 'data.frame':    155 obs. of  9 variables:
##  $ gene            : num  7.63 7.92 7.58 7.29 8 ...
##  $ snp             : num  0 2 1 1 0 0 0 0 0 1 ...
##  $ Fam1            : num  1 0 0 0 1 1 1 1 1 0 ...
##  $ Fam2            : num  0 1 1 1 0 0 0 0 0 1 ...
##  $ Mare            : num  1 1 1 0 0 0 0 1 1 0 ...
##  $ Age             : num  18 18 13 18 19 17 20 19 15 15 ...
##  $ RNAseq_condition: num  0 1 0 0 1 1 1 1 0 0 ...
##  $ Treat           : num  0 0 0 0 0 0 0 0 0 0 ...
##  $ ID              : Factor w/ 82 levels "100","110","111",..: 5 6 7 8 10 12 13 14 16 17 ...
## [1] "chr13.33460982"
## [1] "GLIPR1L2"
## Linear mixed model fit by REML ['lmerMod']
## Formula: 
## gene ~ Fam1 + Fam2 + Mare + Age + snp * Treat * RNAseq_condition +  
##     (1 | ID)
##    Data: comb.df
## 
## REML criterion at convergence: 93.7
## 
## Scaled residuals: 
##      Min       1Q   Median       3Q      Max 
## -2.29726 -0.45033 -0.02664  0.54481  2.24614 
## 
## Random effects:
##  Groups   Name        Variance Std.Dev.
##  ID       (Intercept) 0.06112  0.2472  
##  Residual             0.04371  0.2091  
## Number of obs: 155, groups:  ID, 82
## 
## Fixed effects:
##                             Estimate Std. Error t value
## (Intercept)                 8.258163   0.186901   44.18
## Fam1                       -0.326086   0.083615   -3.90
## Fam2                       -0.405547   0.096254   -4.21
## Mare                       -0.009949   0.067896   -0.15
## Age                        -0.006979   0.009890   -0.71
## snp                        -0.325066   0.129403   -2.51
## Treat                      -0.099422   0.051817   -1.92
## RNAseq_condition           -0.339932   0.086478   -3.93
## snp:Treat                   0.220365   0.115688    1.90
## snp:RNAseq_condition        0.560288   0.153439    3.65
## Treat:RNAseq_condition      0.137108   0.077325    1.77
## snp:Treat:RNAseq_condition -0.323004   0.140996   -2.29
## 
## Correlation of Fixed Effects:
##             (Intr) Fam1   Fam2   Mare   Age    snp    Treat  RNAsq_ snp:Tr
## Fam1        -0.033                                                        
## Fam2        -0.336  0.162                                                 
## Mare        -0.035  0.011 -0.058                                          
## Age         -0.928 -0.065  0.273 -0.160                                   
## snp         -0.083  0.034 -0.221  0.066 -0.050                            
## Treat       -0.133  0.014  0.006  0.012 -0.020  0.214                     
## RNAsq_cndtn -0.363 -0.092  0.122  0.107  0.153  0.259  0.318              
## snp:Treat    0.065 -0.036  0.009 -0.022  0.008 -0.490 -0.448 -0.141       
## snp:RNAsq_c  0.147  0.016  0.011  0.010 -0.043 -0.797 -0.179 -0.435  0.408
## Trt:RNAsq_c  0.101 -0.042 -0.029 -0.014  0.007 -0.140 -0.671 -0.487  0.301
## snp:Tr:RNA_ -0.052  0.042 -0.010  0.024 -0.010  0.404  0.368  0.210 -0.821
##             s:RNA_ T:RNA_
## Fam1                     
## Fam2                     
## Mare                     
## Age                      
## snp                      
## Treat                    
## RNAsq_cndtn              
## snp:Treat                
## snp:RNAsq_c              
## Trt:RNAsq_c  0.218       
## snp:Tr:RNA_ -0.491 -0.444
## Linear mixed-effects model fit by maximum likelihood
##  Data: comb.df 
##        AIC      BIC    logLik
##   75.41543 118.0234 -23.70772
## 
## Random effects:
##  Formula: ~1 | ID
##         (Intercept)  Residual
## StdDev:   0.2323519 0.2034218
## 
## Fixed effects: gene ~ Fam1 + Fam2 + Mare + Age + snp * Treat * RNAseq_condition 
##                                Value  Std.Error DF  t-value p-value
## (Intercept)                 8.258259 0.18471210 74 44.70882  0.0000
## Fam1                       -0.325986 0.08265607 74 -3.94388  0.0002
## Fam2                       -0.405501 0.09512580 74 -4.26278  0.0001
## Mare                       -0.009909 0.06709905 74 -0.14768  0.8830
## Age                        -0.007021 0.00977042 74 -0.71856  0.4747
## snp                        -0.324975 0.12862357 74 -2.52656  0.0137
## Treat                      -0.098814 0.05247150 69 -1.88319  0.0639
## RNAseq_condition           -0.339374 0.08594270 74 -3.94884  0.0002
## snp:Treat                   0.220255 0.11714113 69  1.88025  0.0643
## snp:RNAseq_condition        0.560084 0.15250419 74  3.67258  0.0005
## Treat:RNAseq_condition      0.136492 0.07829435 69  1.74332  0.0857
## snp:Treat:RNAseq_condition -0.322758 0.14277726 69 -2.26057  0.0269
##  Correlation: 
##                            (Intr) Fam1   Fam2   Mare   Age    snp   
## Fam1                       -0.034                                   
## Fam2                       -0.335  0.162                            
## Mare                       -0.035  0.012 -0.058                     
## Age                        -0.927 -0.065  0.273 -0.160              
## snp                        -0.084  0.035 -0.220  0.066 -0.050       
## Treat                      -0.137  0.014  0.006  0.012 -0.020  0.217
## RNAseq_condition           -0.363 -0.090  0.122  0.107  0.152  0.259
## snp:Treat                   0.067 -0.037  0.009 -0.023  0.008 -0.499
## snp:RNAseq_condition        0.147  0.014  0.011  0.010 -0.042 -0.798
## Treat:RNAseq_condition      0.103 -0.043 -0.029 -0.014  0.007 -0.143
## snp:Treat:RNAseq_condition -0.054  0.043 -0.010  0.024 -0.010  0.411
##                            Treat  RNAsq_ snp:Tr s:RNA_ T:RNA_
## Fam1                                                         
## Fam2                                                         
## Mare                                                         
## Age                                                          
## snp                                                          
## Treat                                                        
## RNAseq_condition            0.324                            
## snp:Treat                  -0.448 -0.143                     
## snp:RNAseq_condition       -0.182 -0.435  0.415              
## Treat:RNAseq_condition     -0.671 -0.496  0.301  0.222       
## snp:Treat:RNAseq_condition  0.368  0.213 -0.821 -0.500 -0.444
## 
## Standardized Within-Group Residuals:
##         Min          Q1         Med          Q3         Max 
## -2.38893495 -0.46482251 -0.03083088  0.55473224  2.33651163 
## 
## Number of Observations: 155
## Number of Groups: 82 
## [1] "Fixed Effect Regression Estimates of Mixed Model with REML"
```

```
## [1] "Added Variable aka. Partial Regression Plots: for multiple linear regression (no random effects)"
```

```
## 'data.frame':    155 obs. of  9 variables:
##  $ gene            : num  8.52 8.31 8.42 8.44 8.2 ...
##  $ snp             : num  0 0 1 0 1 0 0 1 1 1 ...
##  $ Fam1            : num  1 0 0 0 1 1 1 1 1 0 ...
##  $ Fam2            : num  0 1 1 1 0 0 0 0 0 1 ...
##  $ Mare            : num  1 1 1 0 0 0 0 1 1 0 ...
##  $ Age             : num  18 18 13 18 19 17 20 19 15 15 ...
##  $ RNAseq_condition: num  0 1 0 0 1 1 1 1 0 0 ...
##  $ Treat           : num  0 0 0 0 0 0 0 0 0 0 ...
##  $ ID              : chr  "26" "29" "30" "31" ...
## 'data.frame':    155 obs. of  9 variables:
##  $ gene            : num  8.52 8.31 8.42 8.44 8.2 ...
##  $ snp             : num  0 0 1 0 1 0 0 1 1 1 ...
##  $ Fam1            : num  1 0 0 0 1 1 1 1 1 0 ...
##  $ Fam2            : num  0 1 1 1 0 0 0 0 0 1 ...
##  $ Mare            : num  1 1 1 0 0 0 0 1 1 0 ...
##  $ Age             : num  18 18 13 18 19 17 20 19 15 15 ...
##  $ RNAseq_condition: num  0 1 0 0 1 1 1 1 0 0 ...
##  $ Treat           : num  0 0 0 0 0 0 0 0 0 0 ...
##  $ ID              : Factor w/ 82 levels "100","110","111",..: 5 6 7 8 10 12 13 14 16 17 ...
## [1] "chr13.33502488"
## [1] "DEXI"
## Linear mixed model fit by REML ['lmerMod']
## Formula: 
## gene ~ Fam1 + Fam2 + Mare + Age + snp * Treat * RNAseq_condition +  
##     (1 | ID)
##    Data: comb.df
## 
## REML criterion at convergence: -70.3
## 
## Scaled residuals: 
##      Min       1Q   Median       3Q      Max 
## -3.01291 -0.54688  0.07684  0.56915  2.10749 
## 
## Random effects:
##  Groups   Name        Variance Std.Dev.
##  ID       (Intercept) 0.005423 0.07364 
##  Residual             0.021569 0.14686 
## Number of obs: 155, groups:  ID, 82
## 
## Fixed effects:
##                             Estimate Std. Error t value
## (Intercept)                 8.220047   0.093127   88.27
## Fam1                        0.164260   0.039344    4.17
## Fam2                        0.133381   0.041940    3.18
## Mare                        0.007924   0.031326    0.25
## Age                        -0.007295   0.004349   -1.68
## snp                        -0.080775   0.036157   -2.23
## Treat                      -0.064970   0.053646   -1.21
## RNAseq_condition           -0.036256   0.061717   -0.59
## snp:Treat                   0.077584   0.043583    1.78
## snp:RNAseq_condition        0.012068   0.051820    0.23
## Treat:RNAseq_condition      0.048998   0.073172    0.67
## snp:Treat:RNAseq_condition -0.062285   0.063416   -0.98
## 
## Correlation of Fixed Effects:
##             (Intr) Fam1   Fam2   Mare   Age    snp    Treat  RNAsq_ snp:Tr
## Fam1        -0.123                                                        
## Fam2        -0.398  0.284                                                 
## Mare        -0.032 -0.061 -0.050                                          
## Age         -0.840 -0.026  0.241 -0.147                                   
## snp         -0.460  0.161  0.234 -0.017  0.051                            
## Treat       -0.301 -0.004  0.026  0.003 -0.019  0.523                     
## RNAsq_cndtn -0.461 -0.023  0.143  0.184  0.094  0.574  0.474              
## snp:Treat    0.243  0.008 -0.017 -0.002  0.008 -0.635 -0.799 -0.376       
## snp:RNAsq_c  0.315  0.013 -0.079 -0.150 -0.029 -0.667 -0.364 -0.765  0.443
## Trt:RNAsq_c  0.241 -0.026 -0.055 -0.013  0.003 -0.394 -0.734 -0.664  0.586
## snp:Tr:RNA_ -0.180  0.007  0.037  0.004  0.003  0.443  0.550  0.494 -0.688
##             s:RNA_ T:RNA_
## Fam1                     
## Fam2                     
## Mare                     
## Age                      
## snp                      
## Treat                    
## RNAsq_cndtn              
## snp:Treat                
## snp:RNAsq_c              
## Trt:RNAsq_c  0.509       
## snp:Tr:RNA_ -0.650 -0.751
## Linear mixed-effects model fit by maximum likelihood
##  Data: comb.df 
##         AIC       BIC  logLik
##   -107.5786 -64.97064 67.7893
## 
## Random effects:
##  Formula: ~1 | ID
##         (Intercept)  Residual
## StdDev:  0.06527504 0.1431842
## 
## Fixed effects: gene ~ Fam1 + Fam2 + Mare + Age + snp * Treat * RNAseq_condition 
##                                Value  Std.Error DF  t-value p-value
## (Intercept)                 8.220455 0.09200960 74 89.34345  0.0000
## Fam1                        0.163839 0.03880912 74  4.22166  0.0001
## Fam2                        0.133302 0.04135347 74  3.22348  0.0019
## Mare                        0.007676 0.03089014 74  0.24851  0.8044
## Age                        -0.007266 0.00428622 74 -1.69529  0.0942
## snp                        -0.081296 0.03604765 74 -2.25523  0.0271
## Treat                      -0.065638 0.05439659 69 -1.20665  0.2317
## RNAseq_condition           -0.037255 0.06157393 74 -0.60504  0.5470
## snp:Treat                   0.078051 0.04421191 69  1.76539  0.0819
## snp:RNAseq_condition        0.012826 0.05169015 74  0.24813  0.8047
## Treat:RNAseq_condition      0.049981 0.07417713 69  0.67381  0.5027
## snp:Treat:RNAseq_condition -0.063003 0.06432453 69 -0.97946  0.3308
##  Correlation: 
##                            (Intr) Fam1   Fam2   Mare   Age    snp   
## Fam1                       -0.123                                   
## Fam2                       -0.397  0.283                            
## Mare                       -0.032 -0.060 -0.049                     
## Age                        -0.838 -0.026  0.240 -0.147              
## snp                        -0.462  0.159  0.232 -0.017  0.050       
## Treat                      -0.309 -0.004  0.026  0.003 -0.019  0.531
## RNAseq_condition           -0.463 -0.021  0.143  0.182  0.093  0.575
## snp:Treat                   0.249  0.008 -0.017 -0.002  0.008 -0.645
## snp:RNAseq_condition        0.317  0.013 -0.079 -0.149 -0.029 -0.668
## Treat:RNAseq_condition      0.247 -0.027 -0.056 -0.013  0.003 -0.400
## snp:Treat:RNAseq_condition -0.185  0.008  0.038  0.004  0.003  0.450
##                            Treat  RNAsq_ snp:Tr s:RNA_ T:RNA_
## Fam1                                                         
## Fam2                                                         
## Mare                                                         
## Age                                                          
## snp                                                          
## Treat                                                        
## RNAseq_condition            0.481                            
## snp:Treat                  -0.799 -0.381                     
## snp:RNAseq_condition       -0.369 -0.765  0.450              
## Treat:RNAseq_condition     -0.734 -0.673  0.586  0.517       
## snp:Treat:RNAseq_condition  0.549  0.501 -0.688 -0.660 -0.750
## 
## Standardized Within-Group Residuals:
##        Min         Q1        Med         Q3        Max 
## -3.1774901 -0.5686236  0.0967636  0.5844383  2.2408557 
## 
## Number of Observations: 155
## Number of Groups: 82 
## [1] "Fixed Effect Regression Estimates of Mixed Model with REML"
```

```
## [1] "Added Variable aka. Partial Regression Plots: for multiple linear regression (no random effects)"
```

```
## 'data.frame':    155 obs. of  9 variables:
##  $ gene            : num  10.01 9.99 10.15 10 9.99 ...
##  $ snp             : num  0 0 1 0 1 0 0 1 1 1 ...
##  $ Fam1            : num  1 0 0 0 1 1 1 1 1 0 ...
##  $ Fam2            : num  0 1 1 1 0 0 0 0 0 1 ...
##  $ Mare            : num  1 1 1 0 0 0 0 1 1 0 ...
##  $ Age             : num  18 18 13 18 19 17 20 19 15 15 ...
##  $ RNAseq_condition: num  0 1 0 0 1 1 1 1 0 0 ...
##  $ Treat           : num  0 0 0 0 0 0 0 0 0 0 ...
##  $ ID              : chr  "26" "29" "30" "31" ...
## 'data.frame':    155 obs. of  9 variables:
##  $ gene            : num  10.01 9.99 10.15 10 9.99 ...
##  $ snp             : num  0 0 1 0 1 0 0 1 1 1 ...
##  $ Fam1            : num  1 0 0 0 1 1 1 1 1 0 ...
##  $ Fam2            : num  0 1 1 1 0 0 0 0 0 1 ...
##  $ Mare            : num  1 1 1 0 0 0 0 1 1 0 ...
##  $ Age             : num  18 18 13 18 19 17 20 19 15 15 ...
##  $ RNAseq_condition: num  0 1 0 0 1 1 1 1 0 0 ...
##  $ Treat           : num  0 0 0 0 0 0 0 0 0 0 ...
##  $ ID              : Factor w/ 82 levels "100","110","111",..: 5 6 7 8 10 12 13 14 16 17 ...
## [1] "chr13.33502488"
## [1] "NSUN2"
## Linear mixed model fit by REML ['lmerMod']
## Formula: 
## gene ~ Fam1 + Fam2 + Mare + Age + snp * Treat * RNAseq_condition +  
##     (1 | ID)
##    Data: comb.df
## 
## REML criterion at convergence: -214.6
## 
## Scaled residuals: 
##      Min       1Q   Median       3Q      Max 
## -1.77387 -0.50207 -0.01975  0.51385  1.74957 
## 
## Random effects:
##  Groups   Name        Variance Std.Dev.
##  ID       (Intercept) 0.006407 0.08004 
##  Residual             0.005174 0.07193 
## Number of obs: 155, groups:  ID, 82
## 
## Fixed effects:
##                              Estimate Std. Error t value
## (Intercept)                 1.016e+01  6.736e-02  150.88
## Fam1                       -4.565e-02  2.886e-02   -1.58
## Fam2                       -8.658e-02  3.091e-02   -2.80
## Mare                        7.728e-04  2.307e-02    0.03
## Age                        -3.085e-03  3.224e-03   -0.96
## snp                        -2.986e-02  2.361e-02   -1.26
## Treat                       1.404e-01  2.660e-02    5.28
## RNAseq_condition           -3.106e-02  3.971e-02   -0.78
## snp:Treat                   2.395e-02  2.150e-02    1.11
## snp:RNAseq_condition        1.672e-02  3.359e-02    0.50
## Treat:RNAseq_condition      1.699e-05  3.638e-02    0.00
## snp:Treat:RNAseq_condition -2.145e-02  3.132e-02   -0.68
## 
## Correlation of Fixed Effects:
##             (Intr) Fam1   Fam2   Mare   Age    snp    Treat  RNAsq_ snp:Tr
## Fam1        -0.124                                                        
## Fam2        -0.402  0.290                                                 
## Mare        -0.032 -0.069 -0.059                                          
## Age         -0.867 -0.027  0.245 -0.142                                   
## snp         -0.432  0.187  0.255 -0.023  0.063                            
## Treat       -0.209 -0.003  0.021  0.002 -0.016  0.404                     
## RNAsq_cndtn -0.440 -0.043  0.129  0.202  0.112  0.564  0.373              
## snp:Treat    0.169  0.007 -0.014 -0.001  0.007 -0.486 -0.801 -0.296       
## snp:RNAsq_c  0.290  0.021 -0.065 -0.168 -0.033 -0.659 -0.282 -0.760  0.341
## Trt:RNAsq_c  0.170 -0.022 -0.046 -0.010  0.002 -0.305 -0.732 -0.525  0.586
## snp:Tr:RNA_ -0.128  0.006  0.031  0.003  0.002  0.340  0.550  0.392 -0.687
##             s:RNA_ T:RNA_
## Fam1                     
## Fam2                     
## Mare                     
## Age                      
## snp                      
## Treat                    
## RNAsq_cndtn              
## snp:Treat                
## snp:RNAsq_c              
## Trt:RNAsq_c  0.399       
## snp:Tr:RNA_ -0.503 -0.754
## Linear mixed-effects model fit by maximum likelihood
##  Data: comb.df 
##         AIC       BIC   logLik
##   -262.4095 -219.8015 145.2047
## 
## Random effects:
##  Formula: ~1 | ID
##         (Intercept)   Residual
## StdDev:  0.07485316 0.07012738
## 
## Fixed effects: gene ~ Fam1 + Fam2 + Mare + Age + snp * Treat * RNAseq_condition 
##                                Value  Std.Error DF   t-value p-value
## (Intercept)                10.163757 0.06650002 74 152.83842  0.0000
## Fam1                       -0.045724 0.02847520 74  -1.60575  0.1126
## Fam2                       -0.086601 0.03048625 74  -2.84066  0.0058
## Mare                        0.000691 0.02275339 74   0.03036  0.9759
## Age                        -0.003095 0.00317848 74  -0.97388  0.3333
## snp                        -0.030027 0.02344469 74  -1.28076  0.2043
## Treat                       0.140377 0.02698064 69   5.20288  0.0000
## RNAseq_condition           -0.031199 0.03947564 74  -0.79034  0.4319
## snp:Treat                   0.024111 0.02181212 69   1.10538  0.2728
## snp:RNAseq_condition        0.016842 0.03336929 74   0.50470  0.6153
## Treat:RNAseq_condition      0.000117 0.03689558 69   0.00316  0.9975
## snp:Treat:RNAseq_condition -0.021549 0.03177194 69  -0.67824  0.4999
##  Correlation: 
##                            (Intr) Fam1   Fam2   Mare   Age    snp   
## Fam1                       -0.124                                   
## Fam2                       -0.402  0.289                            
## Mare                       -0.032 -0.068 -0.059                     
## Age                        -0.866 -0.027  0.245 -0.142              
## snp                        -0.434  0.186  0.254 -0.023  0.062       
## Treat                      -0.215 -0.003  0.022  0.002 -0.016  0.412
## RNAseq_condition           -0.441 -0.041  0.130  0.201  0.111  0.565
## snp:Treat                   0.174  0.007 -0.014 -0.001  0.007 -0.496
## snp:RNAseq_condition        0.291  0.020 -0.066 -0.167 -0.033 -0.660
## Treat:RNAseq_condition      0.174 -0.022 -0.046 -0.010  0.002 -0.311
## snp:Treat:RNAseq_condition -0.131  0.007  0.032  0.003  0.002  0.347
##                            Treat  RNAsq_ snp:Tr s:RNA_ T:RNA_
## Fam1                                                         
## Fam2                                                         
## Mare                                                         
## Age                                                          
## snp                                                          
## Treat                                                        
## RNAseq_condition            0.380                            
## snp:Treat                  -0.801 -0.301                     
## snp:RNAseq_condition       -0.288 -0.761  0.348              
## Treat:RNAseq_condition     -0.732 -0.535  0.586  0.407       
## snp:Treat:RNAseq_condition  0.550  0.400 -0.687 -0.513 -0.754
## 
## Standardized Within-Group Residuals:
##         Min          Q1         Med          Q3         Max 
## -1.86732228 -0.51806243 -0.01861077  0.53545211  1.85213839 
## 
## Number of Observations: 155
## Number of Groups: 82 
## [1] "Fixed Effect Regression Estimates of Mixed Model with REML"
```

```
## [1] "Added Variable aka. Partial Regression Plots: for multiple linear regression (no random effects)"
```

```
## 'data.frame':    155 obs. of  9 variables:
##  $ gene            : num  7.27 6.57 6.05 6.37 6.02 ...
##  $ snp             : num  0 0 1 0 1 0 0 1 1 1 ...
##  $ Fam1            : num  1 0 0 0 1 1 1 1 1 0 ...
##  $ Fam2            : num  0 1 1 1 0 0 0 0 0 1 ...
##  $ Mare            : num  1 1 1 0 0 0 0 1 1 0 ...
##  $ Age             : num  18 18 13 18 19 17 20 19 15 15 ...
##  $ RNAseq_condition: num  0 1 0 0 1 1 1 1 0 0 ...
##  $ Treat           : num  0 0 0 0 0 0 0 0 0 0 ...
##  $ ID              : chr  "26" "29" "30" "31" ...
## 'data.frame':    155 obs. of  9 variables:
##  $ gene            : num  7.27 6.57 6.05 6.37 6.02 ...
##  $ snp             : num  0 0 1 0 1 0 0 1 1 1 ...
##  $ Fam1            : num  1 0 0 0 1 1 1 1 1 0 ...
##  $ Fam2            : num  0 1 1 1 0 0 0 0 0 1 ...
##  $ Mare            : num  1 1 1 0 0 0 0 1 1 0 ...
##  $ Age             : num  18 18 13 18 19 17 20 19 15 15 ...
##  $ RNAseq_condition: num  0 1 0 0 1 1 1 1 0 0 ...
##  $ Treat           : num  0 0 0 0 0 0 0 0 0 0 ...
##  $ ID              : Factor w/ 82 levels "100","110","111",..: 5 6 7 8 10 12 13 14 16 17 ...
## [1] "chr13.33502488"
## [1] "ATF7IP2"
## Linear mixed model fit by REML ['lmerMod']
## Formula: 
## gene ~ Fam1 + Fam2 + Mare + Age + snp * Treat * RNAseq_condition +  
##     (1 | ID)
##    Data: comb.df
## 
## REML criterion at convergence: 264.3
## 
## Scaled residuals: 
##      Min       1Q   Median       3Q      Max 
## -2.91636 -0.46176 -0.08714  0.43486  2.31240 
## 
## Random effects:
##  Groups   Name        Variance Std.Dev.
##  ID       (Intercept) 0.3226   0.5680  
##  Residual             0.1001   0.3163  
## Number of obs: 155, groups:  ID, 82
## 
## Fixed effects:
##                              Estimate Std. Error t value
## (Intercept)                 5.8115663  0.4276660  13.589
## Fam1                       -0.0170151  0.1841492  -0.092
## Fam2                        0.4337305  0.1977266   2.194
## Mare                       -0.1130033  0.1474979  -0.766
## Age                        -0.0002948  0.0206897  -0.014
## snp                         0.5805357  0.1417390   4.096
## Treat                       0.4990492  0.1178227   4.236
## RNAseq_condition            0.5787386  0.2348742   2.464
## snp:Treat                  -0.1253729  0.0949425  -1.321
## snp:RNAseq_condition       -0.3804933  0.2005431  -1.897
## Treat:RNAseq_condition     -0.2270571  0.1613780  -1.407
## snp:Treat:RNAseq_condition  0.2322027  0.1383885   1.678
## 
## Correlation of Fixed Effects:
##             (Intr) Fam1   Fam2   Mare   Age    snp    Treat  RNAsq_ snp:Tr
## Fam1        -0.124                                                        
## Fam2        -0.403  0.293                                                 
## Mare        -0.033 -0.073 -0.064                                          
## Age         -0.881 -0.028  0.247 -0.139                                   
## snp         -0.418  0.203  0.266 -0.027  0.071                            
## Treat       -0.147 -0.002  0.016  0.002 -0.012  0.301                     
## RNAsq_cndtn -0.430 -0.057  0.117  0.214  0.123  0.556  0.282              
## snp:Treat    0.119  0.005 -0.010 -0.001  0.005 -0.360 -0.802 -0.224       
## snp:RNAsq_c  0.276  0.026 -0.054 -0.178 -0.035 -0.653 -0.211 -0.755  0.254
## Trt:RNAsq_c  0.120 -0.017 -0.035 -0.007  0.002 -0.227 -0.730 -0.399  0.586
## snp:Tr:RNA_ -0.091  0.005  0.024  0.002  0.002  0.252  0.550  0.299 -0.686
##             s:RNA_ T:RNA_
## Fam1                     
## Fam2                     
## Mare                     
## Age                      
## snp                      
## Treat                    
## RNAsq_cndtn              
## snp:Treat                
## snp:RNAsq_c              
## Trt:RNAsq_c  0.300       
## snp:Tr:RNA_ -0.375 -0.756
## Linear mixed-effects model fit by maximum likelihood
##  Data: comb.df 
##        AIC      BIC    logLik
##   258.0924 300.7004 -115.0462
## 
## Random effects:
##  Formula: ~1 | ID
##         (Intercept)  Residual
## StdDev:   0.5373101 0.3075888
## 
## Fixed effects: gene ~ Fam1 + Fam2 + Mare + Age + snp * Treat * RNAseq_condition 
##                                Value Std.Error DF   t-value p-value
## (Intercept)                 5.812236 0.4230575 74 13.738641  0.0000
## Fam1                       -0.017019 0.1821286 74 -0.093447  0.9258
## Fam2                        0.433479 0.1955327 74  2.216915  0.0297
## Mare                       -0.112987 0.1458645 74 -0.774600  0.4410
## Age                        -0.000293 0.0204570 74 -0.014316  0.9886
## snp                         0.580269 0.1405823 74  4.127608  0.0001
## Treat                       0.498426 0.1192306 69  4.180352  0.0001
## RNAseq_condition            0.578039 0.2331487 74  2.479273  0.0154
## snp:Treat                  -0.125144 0.0960904 69 -1.302355  0.1971
## snp:RNAseq_condition       -0.380150 0.1989625 74 -1.910663  0.0599
## Treat:RNAseq_condition     -0.226378 0.1632956 69 -1.386306  0.1701
## snp:Treat:RNAseq_condition  0.231866 0.1400578 69  1.655503  0.1024
##  Correlation: 
##                            (Intr) Fam1   Fam2   Mare   Age    snp   
## Fam1                       -0.124                                   
## Fam2                       -0.403  0.293                            
## Mare                       -0.033 -0.073 -0.064                     
## Age                        -0.880 -0.028  0.247 -0.140              
## snp                        -0.419  0.202  0.265 -0.027  0.070       
## Treat                      -0.150 -0.002  0.016  0.002 -0.012  0.307
## RNAseq_condition           -0.430 -0.056  0.117  0.213  0.123  0.557
## snp:Treat                   0.122  0.005 -0.011 -0.001  0.005 -0.367
## snp:RNAseq_condition        0.276  0.025 -0.055 -0.178 -0.035 -0.654
## Treat:RNAseq_condition      0.123 -0.017 -0.035 -0.008  0.002 -0.232
## snp:Treat:RNAseq_condition -0.093  0.005  0.024  0.002  0.002  0.257
##                            Treat  RNAsq_ snp:Tr s:RNA_ T:RNA_
## Fam1                                                         
## Fam2                                                         
## Mare                                                         
## Age                                                          
## snp                                                          
## Treat                                                        
## RNAseq_condition            0.288                            
## snp:Treat                  -0.802 -0.228                     
## snp:RNAseq_condition       -0.215 -0.755  0.259              
## Treat:RNAseq_condition     -0.730 -0.407  0.586  0.306       
## snp:Treat:RNAseq_condition  0.550  0.304 -0.686 -0.383 -0.756
## 
## Standardized Within-Group Residuals:
##        Min         Q1        Med         Q3        Max 
## -3.0150779 -0.4769883 -0.1034326  0.4454083  2.3638288 
## 
## Number of Observations: 155
## Number of Groups: 82 
## [1] "Fixed Effect Regression Estimates of Mixed Model with REML"
```

```
## [1] "Added Variable aka. Partial Regression Plots: for multiple linear regression (no random effects)"
```

```
## 'data.frame':    155 obs. of  9 variables:
##  $ gene            : num  7.63 7.92 7.58 7.29 8 ...
##  $ snp             : num  0 0 1 0 1 0 0 1 1 1 ...
##  $ Fam1            : num  1 0 0 0 1 1 1 1 1 0 ...
##  $ Fam2            : num  0 1 1 1 0 0 0 0 0 1 ...
##  $ Mare            : num  1 1 1 0 0 0 0 1 1 0 ...
##  $ Age             : num  18 18 13 18 19 17 20 19 15 15 ...
##  $ RNAseq_condition: num  0 1 0 0 1 1 1 1 0 0 ...
##  $ Treat           : num  0 0 0 0 0 0 0 0 0 0 ...
##  $ ID              : chr  "26" "29" "30" "31" ...
## 'data.frame':    155 obs. of  9 variables:
##  $ gene            : num  7.63 7.92 7.58 7.29 8 ...
##  $ snp             : num  0 0 1 0 1 0 0 1 1 1 ...
##  $ Fam1            : num  1 0 0 0 1 1 1 1 1 0 ...
##  $ Fam2            : num  0 1 1 1 0 0 0 0 0 1 ...
##  $ Mare            : num  1 1 1 0 0 0 0 1 1 0 ...
##  $ Age             : num  18 18 13 18 19 17 20 19 15 15 ...
##  $ RNAseq_condition: num  0 1 0 0 1 1 1 1 0 0 ...
##  $ Treat           : num  0 0 0 0 0 0 0 0 0 0 ...
##  $ ID              : Factor w/ 82 levels "100","110","111",..: 5 6 7 8 10 12 13 14 16 17 ...
## [1] "chr13.33502488"
## [1] "GLIPR1L2"
## Linear mixed model fit by REML ['lmerMod']
## Formula: 
## gene ~ Fam1 + Fam2 + Mare + Age + snp * Treat * RNAseq_condition +  
##     (1 | ID)
##    Data: comb.df
## 
## REML criterion at convergence: 102.4
## 
## Scaled residuals: 
##      Min       1Q   Median       3Q      Max 
## -2.15492 -0.45910  0.01539  0.45762  2.06475 
## 
## Random effects:
##  Groups   Name        Variance Std.Dev.
##  ID       (Intercept) 0.06704  0.2589  
##  Residual             0.04399  0.2097  
## Number of obs: 155, groups:  ID, 82
## 
## Fixed effects:
##                             Estimate Std. Error t value
## (Intercept)                 7.945176   0.211210   37.62
## Fam1                       -0.286705   0.090618   -3.16
## Fam2                       -0.319510   0.097106   -3.29
## Mare                       -0.036847   0.072463   -0.51
## Age                        -0.003124   0.010136   -0.31
## snp                         0.170240   0.073032    2.33
## Treat                       0.071847   0.077700    0.92
## RNAseq_condition           -0.044709   0.122439   -0.37
## snp:Treat                  -0.127171   0.062752   -2.03
## snp:RNAseq_condition       -0.143213   0.103768   -1.38
## Treat:RNAseq_condition     -0.121975   0.106308   -1.15
## snp:Treat:RNAseq_condition  0.195106   0.091425    2.13
## 
## Correlation of Fixed Effects:
##             (Intr) Fam1   Fam2   Mare   Age    snp    Treat  RNAsq_ snp:Tr
## Fam1        -0.124                                                        
## Fam2        -0.403  0.291                                                 
## Mare        -0.032 -0.070 -0.060                                          
## Age         -0.871 -0.027  0.246 -0.142                                   
## snp         -0.429  0.191  0.257 -0.024  0.065                            
## Treat       -0.195 -0.003  0.020  0.002 -0.015  0.382                     
## RNAsq_cndtn -0.437 -0.046  0.126  0.205  0.115  0.562  0.354              
## snp:Treat    0.158  0.006 -0.013 -0.001  0.006 -0.459 -0.801 -0.281       
## snp:RNAsq_c  0.286  0.022 -0.062 -0.170 -0.034 -0.658 -0.268 -0.759  0.323
## Trt:RNAsq_c  0.159 -0.021 -0.044 -0.009  0.002 -0.289 -0.731 -0.500  0.586
## snp:Tr:RNA_ -0.119  0.006  0.030  0.003  0.002  0.321  0.550  0.373 -0.687
##             s:RNA_ T:RNA_
## Fam1                     
## Fam2                     
## Mare                     
## Age                      
## snp                      
## Treat                    
## RNAsq_cndtn              
## snp:Treat                
## snp:RNAsq_c              
## Trt:RNAsq_c  0.379       
## snp:Tr:RNA_ -0.476 -0.754
## Linear mixed-effects model fit by maximum likelihood
##  Data: comb.df 
##        AIC      BIC    logLik
##   81.55558 124.1635 -26.77779
## 
## Random effects:
##  Formula: ~1 | ID
##         (Intercept)  Residual
## StdDev:   0.2435469 0.2040826
## 
## Fixed effects: gene ~ Fam1 + Fam2 + Mare + Age + snp * Treat * RNAseq_condition 
##                                Value  Std.Error DF  t-value p-value
## (Intercept)                 7.945122 0.20885382 74 38.04154  0.0000
## Fam1                       -0.286487 0.08956867 74 -3.19852  0.0020
## Fam2                       -0.319458 0.09596146 74 -3.32902  0.0014
## Mare                       -0.036761 0.07161193 74 -0.51334  0.6092
## Age                        -0.003169 0.01001363 74 -0.31643  0.7526
## snp                         0.170412 0.07253604 74  2.34934  0.0215
## Treat                       0.072589 0.07866609 69  0.92275  0.3594
## RNAseq_condition           -0.044053 0.12173355 74 -0.36188  0.7185
## snp:Treat                  -0.127319 0.06354652 69 -2.00355  0.0490
## snp:RNAseq_condition       -0.143354 0.10310522 74 -1.39037  0.1686
## Treat:RNAseq_condition     -0.122682 0.10761699 69 -1.13998  0.2582
## snp:Treat:RNAseq_condition  0.195251 0.09257838 69  2.10903  0.0386
##  Correlation: 
##                            (Intr) Fam1   Fam2   Mare   Age    snp   
## Fam1                       -0.124                                   
## Fam2                       -0.402  0.290                            
## Mare                       -0.032 -0.069 -0.060                     
## Age                        -0.870 -0.027  0.245 -0.142              
## snp                        -0.430  0.190  0.257 -0.024  0.064       
## Treat                      -0.200 -0.003  0.021  0.002 -0.015  0.389
## RNAseq_condition           -0.438 -0.045  0.127  0.204  0.114  0.563
## snp:Treat                   0.162  0.006 -0.013 -0.001  0.007 -0.468
## snp:RNAseq_condition        0.287  0.021 -0.063 -0.170 -0.033 -0.658
## Treat:RNAseq_condition      0.163 -0.021 -0.044 -0.010  0.002 -0.294
## snp:Treat:RNAseq_condition -0.122  0.006  0.030  0.003  0.002  0.327
##                            Treat  RNAsq_ snp:Tr s:RNA_ T:RNA_
## Fam1                                                         
## Fam2                                                         
## Mare                                                         
## Age                                                          
## snp                                                          
## Treat                                                        
## RNAseq_condition            0.360                            
## snp:Treat                  -0.801 -0.286                     
## snp:RNAseq_condition       -0.272 -0.759  0.329              
## Treat:RNAseq_condition     -0.731 -0.508  0.586  0.385       
## snp:Treat:RNAseq_condition  0.550  0.380 -0.687 -0.485 -0.754
## 
## Standardized Within-Group Residuals:
##          Min           Q1          Med           Q3          Max 
## -2.241004733 -0.480366018  0.006721474  0.469639138  2.143373887 
## 
## Number of Observations: 155
## Number of Groups: 82 
## [1] "Fixed Effect Regression Estimates of Mixed Model with REML"
```

```
## [1] "Added Variable aka. Partial Regression Plots: for multiple linear regression (no random effects)"
```

```
## 'data.frame':    155 obs. of  9 variables:
##  $ gene            : num  8.52 8.31 8.42 8.44 8.2 ...
##  $ snp             : num  0 1 0 0 0 0 0 0 1 1 ...
##  $ Fam1            : num  1 0 0 0 1 1 1 1 1 0 ...
##  $ Fam2            : num  0 1 1 1 0 0 0 0 0 1 ...
##  $ Mare            : num  1 1 1 0 0 0 0 1 1 0 ...
##  $ Age             : num  18 18 13 18 19 17 20 19 15 15 ...
##  $ RNAseq_condition: num  0 1 0 0 1 1 1 1 0 0 ...
##  $ Treat           : num  0 0 0 0 0 0 0 0 0 0 ...
##  $ ID              : chr  "26" "29" "30" "31" ...
## 'data.frame':    155 obs. of  9 variables:
##  $ gene            : num  8.52 8.31 8.42 8.44 8.2 ...
##  $ snp             : num  0 1 0 0 0 0 0 0 1 1 ...
##  $ Fam1            : num  1 0 0 0 1 1 1 1 1 0 ...
##  $ Fam2            : num  0 1 1 1 0 0 0 0 0 1 ...
##  $ Mare            : num  1 1 1 0 0 0 0 1 1 0 ...
##  $ Age             : num  18 18 13 18 19 17 20 19 15 15 ...
##  $ RNAseq_condition: num  0 1 0 0 1 1 1 1 0 0 ...
##  $ Treat           : num  0 0 0 0 0 0 0 0 0 0 ...
##  $ ID              : Factor w/ 82 levels "100","110","111",..: 5 6 7 8 10 12 13 14 16 17 ...
## [1] "chr28.3692072"
## [1] "DEXI"
## Linear mixed model fit by REML ['lmerMod']
## Formula: 
## gene ~ Fam1 + Fam2 + Mare + Age + snp * Treat * RNAseq_condition +  
##     (1 | ID)
##    Data: comb.df
## 
## REML criterion at convergence: -78.1
## 
## Scaled residuals: 
##      Min       1Q   Median       3Q      Max 
## -2.31660 -0.53495  0.04509  0.61428  1.99810 
## 
## Random effects:
##  Groups   Name        Variance Std.Dev.
##  ID       (Intercept) 0.007897 0.08887 
##  Residual             0.018953 0.13767 
## Number of obs: 155, groups:  ID, 82
## 
## Fixed effects:
##                             Estimate Std. Error t value
## (Intercept)                 8.216015   0.090732   90.55
## Fam1                        0.190103   0.038915    4.89
## Fam2                        0.145335   0.042280    3.44
## Mare                       -0.018956   0.031823   -0.60
## Age                        -0.007618   0.004522   -1.68
## snp                        -0.163072   0.049363   -3.30
## Treat                      -0.061296   0.038199   -1.60
## RNAseq_condition           -0.073240   0.048284   -1.52
## snp:Treat                   0.183921   0.054903    3.35
## snp:RNAseq_condition        0.138937   0.083718    1.66
## Treat:RNAseq_condition      0.063615   0.053779    1.18
## snp:Treat:RNAseq_condition -0.207404   0.095861   -2.16
## 
## Correlation of Fixed Effects:
##             (Intr) Fam1   Fam2   Mare   Age    snp    Treat  RNAsq_ snp:Tr
## Fam1        -0.033                                                        
## Fam2        -0.377  0.204                                                 
## Mare        -0.108  0.000  0.052                                          
## Age         -0.893 -0.060  0.235 -0.126                                   
## snp         -0.332 -0.003  0.202  0.166  0.070                            
## Treat       -0.219 -0.006  0.004 -0.005  0.007  0.355                     
## RNAsq_cndtn -0.440 -0.078  0.180  0.202  0.155  0.463  0.402              
## snp:Treat    0.177  0.006 -0.013 -0.004 -0.046 -0.648 -0.607 -0.262       
## snp:RNAsq_c  0.263  0.069 -0.121 -0.122 -0.115 -0.600 -0.210 -0.557  0.386
## Trt:RNAsq_c  0.175 -0.031 -0.037 -0.005 -0.017 -0.259 -0.710 -0.597  0.432
## snp:Tr:RNA_ -0.112  0.016  0.027  0.007  0.033  0.375  0.348  0.325 -0.573
##             s:RNA_ T:RNA_
## Fam1                     
## Fam2                     
## Mare                     
## Age                      
## snp                      
## Treat                    
## RNAsq_cndtn              
## snp:Treat                
## snp:RNAsq_c              
## Trt:RNAsq_c  0.329       
## snp:Tr:RNA_ -0.617 -0.525
## Linear mixed-effects model fit by maximum likelihood
##  Data: comb.df 
##         AIC       BIC  logLik
##   -111.9884 -69.38044 69.9942
## 
## Random effects:
##  Formula: ~1 | ID
##         (Intercept)  Residual
## StdDev:  0.08099679 0.1342026
## 
## Fixed effects: gene ~ Fam1 + Fam2 + Mare + Age + snp * Treat * RNAseq_condition 
##                                Value  Std.Error DF  t-value p-value
## (Intercept)                 8.215588 0.08958383 74 91.70838  0.0000
## Fam1                        0.189769 0.03839762 74  4.94222  0.0000
## Fam2                        0.145342 0.04170281 74  3.48519  0.0008
## Mare                       -0.019155 0.03138851 74 -0.61027  0.5436
## Age                        -0.007583 0.00445795 74 -1.70093  0.0932
## snp                        -0.162640 0.04922162 74 -3.30424  0.0015
## Treat                      -0.061332 0.03876238 69 -1.58225  0.1182
## RNAseq_condition           -0.073414 0.04808591 74 -1.52673  0.1311
## snp:Treat                   0.183456 0.05562962 69  3.29781  0.0015
## snp:RNAseq_condition        0.138596 0.08342461 74  1.66133  0.1009
## Treat:RNAseq_condition      0.063829 0.05454465 69  1.17022  0.2459
## snp:Treat:RNAseq_condition -0.207117 0.09721908 69 -2.13042  0.0367
##  Correlation: 
##                            (Intr) Fam1   Fam2   Mare   Age    snp   
## Fam1                       -0.033                                   
## Fam2                       -0.377  0.204                            
## Mare                       -0.107  0.000  0.052                     
## Age                        -0.892 -0.060  0.235 -0.127              
## snp                        -0.334 -0.003  0.200  0.165  0.071       
## Treat                      -0.225 -0.006  0.004 -0.005  0.007  0.360
## RNAseq_condition           -0.440 -0.076  0.180  0.200  0.154  0.462
## snp:Treat                   0.181  0.006 -0.013 -0.005 -0.046 -0.657
## snp:RNAseq_condition        0.264  0.067 -0.121 -0.121 -0.115 -0.600
## Treat:RNAseq_condition      0.179 -0.032 -0.038 -0.005 -0.017 -0.263
## snp:Treat:RNAseq_condition -0.115  0.016  0.027  0.007  0.033  0.380
##                            Treat  RNAsq_ snp:Tr s:RNA_ T:RNA_
## Fam1                                                         
## Fam2                                                         
## Mare                                                         
## Age                                                          
## snp                                                          
## Treat                                                        
## RNAseq_condition            0.410                            
## snp:Treat                  -0.607 -0.267                     
## snp:RNAseq_condition       -0.213 -0.557  0.392              
## Treat:RNAseq_condition     -0.711 -0.608  0.432  0.334       
## snp:Treat:RNAseq_condition  0.347  0.330 -0.573 -0.627 -0.525
## 
## Standardized Within-Group Residuals:
##         Min          Q1         Med          Q3         Max 
## -2.46892726 -0.56300840  0.04941785  0.64355310  2.12934669 
## 
## Number of Observations: 155
## Number of Groups: 82 
## [1] "Fixed Effect Regression Estimates of Mixed Model with REML"
```

```
## [1] "Added Variable aka. Partial Regression Plots: for multiple linear regression (no random effects)"
```

```
## 'data.frame':    155 obs. of  9 variables:
##  $ gene            : num  10.01 9.99 10.15 10 9.99 ...
##  $ snp             : num  0 1 0 0 0 0 0 0 1 1 ...
##  $ Fam1            : num  1 0 0 0 1 1 1 1 1 0 ...
##  $ Fam2            : num  0 1 1 1 0 0 0 0 0 1 ...
##  $ Mare            : num  1 1 1 0 0 0 0 1 1 0 ...
##  $ Age             : num  18 18 13 18 19 17 20 19 15 15 ...
##  $ RNAseq_condition: num  0 1 0 0 1 1 1 1 0 0 ...
##  $ Treat           : num  0 0 0 0 0 0 0 0 0 0 ...
##  $ ID              : chr  "26" "29" "30" "31" ...
## 'data.frame':    155 obs. of  9 variables:
##  $ gene            : num  10.01 9.99 10.15 10 9.99 ...
##  $ snp             : num  0 1 0 0 0 0 0 0 1 1 ...
##  $ Fam1            : num  1 0 0 0 1 1 1 1 1 0 ...
##  $ Fam2            : num  0 1 1 1 0 0 0 0 0 1 ...
##  $ Mare            : num  1 1 1 0 0 0 0 1 1 0 ...
##  $ Age             : num  18 18 13 18 19 17 20 19 15 15 ...
##  $ RNAseq_condition: num  0 1 0 0 1 1 1 1 0 0 ...
##  $ Treat           : num  0 0 0 0 0 0 0 0 0 0 ...
##  $ ID              : Factor w/ 82 levels "100","110","111",..: 5 6 7 8 10 12 13 14 16 17 ...
## [1] "chr28.3692072"
## [1] "NSUN2"
## Linear mixed model fit by REML ['lmerMod']
## Formula: 
## gene ~ Fam1 + Fam2 + Mare + Age + snp * Treat * RNAseq_condition +  
##     (1 | ID)
##    Data: comb.df
## 
## REML criterion at convergence: -218.8
## 
## Scaled residuals: 
##      Min       1Q   Median       3Q      Max 
## -1.90382 -0.40171 -0.01164  0.53171  1.76024 
## 
## Random effects:
##  Groups   Name        Variance Std.Dev.
##  ID       (Intercept) 0.006356 0.07973 
##  Residual             0.005154 0.07179 
## Number of obs: 155, groups:  ID, 82
## 
## Fixed effects:
##                             Estimate Std. Error t value
## (Intercept)                10.111441   0.064006  157.98
## Fam1                       -0.038827   0.027570   -1.41
## Fam2                       -0.067649   0.030054   -2.25
## Mare                        0.003622   0.022624    0.16
## Age                        -0.002737   0.003227   -0.85
## snp                         0.019370   0.031674    0.61
## Treat                       0.153085   0.019948    7.67
## RNAseq_condition           -0.001155   0.031512   -0.04
## snp:Treat                   0.024888   0.029070    0.86
## snp:RNAseq_condition       -0.016086   0.054292   -0.30
## Treat:RNAseq_condition     -0.004505   0.028214   -0.16
## snp:Treat:RNAseq_condition -0.052389   0.050322   -1.04
## 
## Correlation of Fixed Effects:
##             (Intr) Fam1   Fam2   Mare   Age    snp    Treat  RNAsq_ snp:Tr
## Fam1        -0.031                                                        
## Fam2        -0.379  0.208                                                 
## Mare        -0.110 -0.004  0.048                                          
## Age         -0.902 -0.063  0.234 -0.125                                   
## snp         -0.314  0.000  0.217  0.182  0.057                            
## Treat       -0.163 -0.005  0.003 -0.004  0.006  0.293                     
## RNAsq_cndtn -0.433 -0.099  0.178  0.217  0.162  0.472  0.323              
## snp:Treat    0.137  0.005 -0.011 -0.004 -0.039 -0.547 -0.603 -0.211       
## snp:RNAsq_c  0.252  0.083 -0.120 -0.131 -0.111 -0.592 -0.172 -0.557  0.323
## Trt:RNAsq_c  0.132 -0.027 -0.032 -0.004 -0.014 -0.214 -0.707 -0.486  0.427
## snp:Tr:RNA_ -0.088  0.014  0.023  0.006  0.028  0.320  0.348  0.266 -0.578
##             s:RNA_ T:RNA_
## Fam1                     
## Fam2                     
## Mare                     
## Age                      
## snp                      
## Treat                    
## RNAsq_cndtn              
## snp:Treat                
## snp:RNAsq_c              
## Trt:RNAsq_c  0.271       
## snp:Tr:RNA_ -0.506 -0.527
## Linear mixed-effects model fit by maximum likelihood
##  Data: comb.df 
##         AIC       BIC   logLik
##   -263.2356 -220.6276 145.6178
## 
## Random effects:
##  Formula: ~1 | ID
##         (Intercept)   Residual
## StdDev:  0.07461569 0.06996077
## 
## Fixed effects: gene ~ Fam1 + Fam2 + Mare + Age + snp * Treat * RNAseq_condition 
##                                Value  Std.Error DF   t-value p-value
## (Intercept)                10.111792 0.06319285 74 160.01480  0.0000
## Fam1                       -0.038868 0.02721327 74  -1.42826  0.1574
## Fam2                       -0.067692 0.02965811 74  -2.28240  0.0253
## Mare                        0.003491 0.02232520 74   0.15637  0.8762
## Age                        -0.002746 0.00318310 74  -0.86272  0.3911
## snp                         0.018901 0.03150666 74   0.59991  0.5504
## Treat                       0.152998 0.02023663 69   7.56045  0.0000
## RNAseq_condition           -0.001437 0.03129408 74  -0.04592  0.9635
## snp:Treat                   0.025320 0.02946022 69   0.85945  0.3931
## snp:RNAseq_condition       -0.015462 0.05394409 74  -0.28664  0.7752
## Treat:RNAseq_condition     -0.004275 0.02861260 69  -0.14942  0.8817
## snp:Treat:RNAseq_condition -0.052962 0.05103105 69  -1.03785  0.3030
##  Correlation: 
##                            (Intr) Fam1   Fam2   Mare   Age    snp   
## Fam1                       -0.031                                   
## Fam2                       -0.379  0.208                            
## Mare                       -0.109 -0.004  0.048                     
## Age                        -0.902 -0.063  0.234 -0.125              
## snp                        -0.315  0.000  0.215  0.181  0.058       
## Treat                      -0.167 -0.005  0.003 -0.005  0.006  0.299
## RNAseq_condition           -0.433 -0.097  0.178  0.216  0.162  0.472
## snp:Treat                   0.140  0.005 -0.011 -0.004 -0.040 -0.556
## snp:RNAseq_condition        0.253  0.082 -0.120 -0.130 -0.112 -0.593
## Treat:RNAseq_condition      0.136 -0.027 -0.033 -0.004 -0.015 -0.218
## snp:Treat:RNAseq_condition -0.090  0.014  0.023  0.006  0.029  0.325
##                            Treat  RNAsq_ snp:Tr s:RNA_ T:RNA_
## Fam1                                                         
## Fam2                                                         
## Mare                                                         
## Age                                                          
## snp                                                          
## Treat                                                        
## RNAseq_condition            0.330                            
## snp:Treat                  -0.603 -0.216                     
## snp:RNAseq_condition       -0.175 -0.557  0.328              
## Treat:RNAseq_condition     -0.707 -0.496  0.427  0.276       
## snp:Treat:RNAseq_condition  0.348  0.271 -0.578 -0.516 -0.527
## 
## Standardized Within-Group Residuals:
##         Min          Q1         Med          Q3         Max 
## -1.98986739 -0.41060220 -0.01795672  0.55215317  1.83254278 
## 
## Number of Observations: 155
## Number of Groups: 82 
## [1] "Fixed Effect Regression Estimates of Mixed Model with REML"
```

```
## [1] "Added Variable aka. Partial Regression Plots: for multiple linear regression (no random effects)"
```

```
## 'data.frame':    155 obs. of  9 variables:
##  $ gene            : num  7.27 6.57 6.05 6.37 6.02 ...
##  $ snp             : num  0 1 0 0 0 0 0 0 1 1 ...
##  $ Fam1            : num  1 0 0 0 1 1 1 1 1 0 ...
##  $ Fam2            : num  0 1 1 1 0 0 0 0 0 1 ...
##  $ Mare            : num  1 1 1 0 0 0 0 1 1 0 ...
##  $ Age             : num  18 18 13 18 19 17 20 19 15 15 ...
##  $ RNAseq_condition: num  0 1 0 0 1 1 1 1 0 0 ...
##  $ Treat           : num  0 0 0 0 0 0 0 0 0 0 ...
##  $ ID              : chr  "26" "29" "30" "31" ...
## 'data.frame':    155 obs. of  9 variables:
##  $ gene            : num  7.27 6.57 6.05 6.37 6.02 ...
##  $ snp             : num  0 1 0 0 0 0 0 0 1 1 ...
##  $ Fam1            : num  1 0 0 0 1 1 1 1 1 0 ...
##  $ Fam2            : num  0 1 1 1 0 0 0 0 0 1 ...
##  $ Mare            : num  1 1 1 0 0 0 0 1 1 0 ...
##  $ Age             : num  18 18 13 18 19 17 20 19 15 15 ...
##  $ RNAseq_condition: num  0 1 0 0 1 1 1 1 0 0 ...
##  $ Treat           : num  0 0 0 0 0 0 0 0 0 0 ...
##  $ ID              : Factor w/ 82 levels "100","110","111",..: 5 6 7 8 10 12 13 14 16 17 ...
## [1] "chr28.3692072"
## [1] "ATF7IP2"
## Linear mixed model fit by REML ['lmerMod']
## Formula: 
## gene ~ Fam1 + Fam2 + Mare + Age + snp * Treat * RNAseq_condition +  
##     (1 | ID)
##    Data: comb.df
## 
## REML criterion at convergence: 269
## 
## Scaled residuals: 
##     Min      1Q  Median      3Q     Max 
## -2.8930 -0.4574 -0.1257  0.4109  2.5092 
## 
## Random effects:
##  Groups   Name        Variance Std.Dev.
##  ID       (Intercept) 0.3558   0.5965  
##  Residual             0.1025   0.3202  
## Number of obs: 155, groups:  ID, 82
## 
## Fixed effects:
##                             Estimate Std. Error t value
## (Intercept)                 6.663143   0.426962  15.606
## Fam1                       -0.162448   0.184231  -0.882
## Fam2                        0.162578   0.201392   0.807
## Mare                       -0.059976   0.151613  -0.396
## Age                        -0.013337   0.021692  -0.615
## snp                         0.010094   0.193724   0.052
## Treat                       0.420123   0.089075   4.717
## RNAseq_condition            0.005473   0.197268   0.028
## snp:Treat                  -0.121996   0.131318  -0.929
## snp:RNAseq_condition        0.652581   0.337670   1.933
## Treat:RNAseq_condition     -0.105437   0.126476  -0.834
## snp:Treat:RNAseq_condition  0.312698   0.225705   1.385
## 
## Correlation of Fixed Effects:
##             (Intr) Fam1   Fam2   Mare   Age    snp    Treat  RNAsq_ snp:Tr
## Fam1        -0.029                                                        
## Fam2        -0.380  0.211                                                 
## Mare        -0.111 -0.008  0.045                                          
## Age         -0.909 -0.065  0.234 -0.123                                   
## snp         -0.298  0.003  0.231  0.197  0.043                            
## Treat       -0.110 -0.004  0.002 -0.003  0.004  0.217                     
## RNAsq_cndtn -0.429 -0.119  0.174  0.231  0.168  0.485  0.231              
## snp:Treat    0.095  0.004 -0.008 -0.003 -0.029 -0.411 -0.599 -0.151       
## snp:RNAsq_c  0.242  0.096 -0.117 -0.138 -0.106 -0.580 -0.125 -0.555  0.239
## Trt:RNAsq_c  0.090 -0.020 -0.024 -0.003 -0.011 -0.158 -0.704 -0.351  0.422
## snp:Tr:RNA_ -0.062  0.010  0.017  0.005  0.021  0.242  0.349  0.194 -0.582
##             s:RNA_ T:RNA_
## Fam1                     
## Fam2                     
## Mare                     
## Age                      
## snp                      
## Treat                    
## RNAsq_cndtn              
## snp:Treat                
## snp:RNAsq_c              
## Trt:RNAsq_c  0.198       
## snp:Tr:RNA_ -0.369 -0.528
## Linear mixed-effects model fit by maximum likelihood
##  Data: comb.df 
##        AIC      BIC    logLik
##   267.0338 309.6417 -119.5169
## 
## Random effects:
##  Formula: ~1 | ID
##         (Intercept) Residual
## StdDev:   0.5644142 0.311339
## 
## Fixed effects: gene ~ Fam1 + Fam2 + Mare + Age + snp * Treat * RNAseq_condition 
##                                Value Std.Error DF   t-value p-value
## (Intercept)                 6.662660 0.4222784 74 15.777886  0.0000
## Fam1                       -0.162472 0.1822016 74 -0.891716  0.3754
## Fam2                        0.162489 0.1991497 74  0.815915  0.4172
## Mare                       -0.059857 0.1499248 74 -0.399249  0.6909
## Age                        -0.013324 0.0214477 74 -0.621221  0.5364
## snp                         0.011870 0.1923426 74  0.061714  0.9510
## Treat                       0.420330 0.0901724 69  4.661406  0.0000
## RNAseq_condition            0.005495 0.1956289 74  0.028091  0.9777
## snp:Treat                  -0.123764 0.1328714 69 -0.931458  0.3549
## snp:RNAseq_condition        0.651001 0.3349652 74  1.943489  0.0558
## Treat:RNAseq_condition     -0.105413 0.1280131 69 -0.823455  0.4131
## snp:Treat:RNAseq_condition  0.314236 0.2284437 69  1.375550  0.1734
##  Correlation: 
##                            (Intr) Fam1   Fam2   Mare   Age    snp   
## Fam1                       -0.029                                   
## Fam2                       -0.380  0.211                            
## Mare                       -0.111 -0.008  0.045                     
## Age                        -0.908 -0.065  0.234 -0.123              
## snp                        -0.299  0.003  0.231  0.196  0.044       
## Treat                      -0.112 -0.004  0.002 -0.003  0.005  0.221
## RNAseq_condition           -0.429 -0.118  0.174  0.230  0.167  0.484
## snp:Treat                   0.097  0.004 -0.008 -0.003 -0.030 -0.419
## snp:RNAseq_condition        0.243  0.096 -0.117 -0.138 -0.107 -0.580
## Treat:RNAseq_condition      0.092 -0.020 -0.024 -0.003 -0.011 -0.161
## snp:Treat:RNAseq_condition -0.063  0.011  0.018  0.005  0.022  0.246
##                            Treat  RNAsq_ snp:Tr s:RNA_ T:RNA_
## Fam1                                                         
## Fam2                                                         
## Mare                                                         
## Age                                                          
## snp                                                          
## Treat                                                        
## RNAseq_condition            0.236                            
## snp:Treat                  -0.600 -0.155                     
## snp:RNAseq_condition       -0.128 -0.555  0.243              
## Treat:RNAseq_condition     -0.704 -0.358  0.423  0.202       
## snp:Treat:RNAseq_condition  0.349  0.198 -0.582 -0.376 -0.528
## 
## Standardized Within-Group Residuals:
##        Min         Q1        Med         Q3        Max 
## -2.9875082 -0.4640750 -0.1327202  0.4206953  2.5730156 
## 
## Number of Observations: 155
## Number of Groups: 82 
## [1] "Fixed Effect Regression Estimates of Mixed Model with REML"
```

```
## [1] "Added Variable aka. Partial Regression Plots: for multiple linear regression (no random effects)"
```

```
## 'data.frame':    155 obs. of  9 variables:
##  $ gene            : num  7.63 7.92 7.58 7.29 8 ...
##  $ snp             : num  0 1 0 0 0 0 0 0 1 1 ...
##  $ Fam1            : num  1 0 0 0 1 1 1 1 1 0 ...
##  $ Fam2            : num  0 1 1 1 0 0 0 0 0 1 ...
##  $ Mare            : num  1 1 1 0 0 0 0 1 1 0 ...
##  $ Age             : num  18 18 13 18 19 17 20 19 15 15 ...
##  $ RNAseq_condition: num  0 1 0 0 1 1 1 1 0 0 ...
##  $ Treat           : num  0 0 0 0 0 0 0 0 0 0 ...
##  $ ID              : chr  "26" "29" "30" "31" ...
## 'data.frame':    155 obs. of  9 variables:
##  $ gene            : num  7.63 7.92 7.58 7.29 8 ...
##  $ snp             : num  0 1 0 0 0 0 0 0 1 1 ...
##  $ Fam1            : num  1 0 0 0 1 1 1 1 1 0 ...
##  $ Fam2            : num  0 1 1 1 0 0 0 0 0 1 ...
##  $ Mare            : num  1 1 1 0 0 0 0 1 1 0 ...
##  $ Age             : num  18 18 13 18 19 17 20 19 15 15 ...
##  $ RNAseq_condition: num  0 1 0 0 1 1 1 1 0 0 ...
##  $ Treat           : num  0 0 0 0 0 0 0 0 0 0 ...
##  $ ID              : Factor w/ 82 levels "100","110","111",..: 5 6 7 8 10 12 13 14 16 17 ...
## [1] "chr28.3692072"
## [1] "GLIPR1L2"
## Linear mixed model fit by REML ['lmerMod']
## Formula: 
## gene ~ Fam1 + Fam2 + Mare + Age + snp * Treat * RNAseq_condition +  
##     (1 | ID)
##    Data: comb.df
## 
## REML criterion at convergence: 97.9
## 
## Scaled residuals: 
##      Min       1Q   Median       3Q      Max 
## -2.37125 -0.41705 -0.06204  0.55116  1.72661 
## 
## Random effects:
##  Groups   Name        Variance Std.Dev.
##  ID       (Intercept) 0.06066  0.2463  
##  Residual             0.04612  0.2147  
## Number of obs: 155, groups:  ID, 82
## 
## Fixed effects:
##                             Estimate Std. Error t value
## (Intercept)                 8.041153   0.195772   41.07
## Fam1                       -0.316030   0.084341   -3.75
## Fam2                       -0.320511   0.091960   -3.49
## Mare                        0.015544   0.069224    0.22
## Age                        -0.005268   0.009875   -0.53
## snp                         0.198279   0.096303    2.06
## Treat                      -0.065218   0.059676   -1.09
## RNAseq_condition           -0.151153   0.095943   -1.58
## snp:Treat                   0.009400   0.087039    0.11
## snp:RNAseq_condition        0.036550   0.165227    0.22
## Treat:RNAseq_condition      0.089458   0.084429    1.06
## snp:Treat:RNAseq_condition -0.098019   0.150593   -0.65
## 
## Correlation of Fixed Effects:
##             (Intr) Fam1   Fam2   Mare   Age    snp    Treat  RNAsq_ snp:Tr
## Fam1        -0.031                                                        
## Fam2        -0.379  0.208                                                 
## Mare        -0.110 -0.005  0.048                                          
## Age         -0.903 -0.063  0.234 -0.125                                   
## snp         -0.312  0.000  0.218  0.183  0.056                            
## Treat       -0.160 -0.005  0.003 -0.004  0.006  0.289                     
## RNAsq_cndtn -0.432 -0.100  0.178  0.218  0.163  0.473  0.317              
## snp:Treat    0.134  0.005 -0.011 -0.004 -0.039 -0.539 -0.602 -0.208       
## snp:RNAsq_c  0.252  0.084 -0.120 -0.131 -0.111 -0.591 -0.169 -0.557  0.318
## Trt:RNAsq_c  0.129 -0.026 -0.032 -0.004 -0.014 -0.211 -0.707 -0.478  0.426
## snp:Tr:RNA_ -0.087  0.014  0.023  0.006  0.028  0.315  0.348  0.262 -0.578
##             s:RNA_ T:RNA_
## Fam1                     
## Fam2                     
## Mare                     
## Age                      
## snp                      
## Treat                    
## RNAsq_cndtn              
## snp:Treat                
## snp:RNAsq_c              
## Trt:RNAsq_c  0.266       
## snp:Tr:RNA_ -0.498 -0.527
## Linear mixed-effects model fit by maximum likelihood
##  Data: comb.df 
##        AIC      BIC    logLik
##   80.15238 122.7603 -26.07619
## 
## Random effects:
##  Formula: ~1 | ID
##         (Intercept) Residual
## StdDev:   0.2315339 0.208849
## 
## Fixed effects: gene ~ Fam1 + Fam2 + Mare + Age + snp * Treat * RNAseq_condition 
##                                Value  Std.Error DF  t-value p-value
## (Intercept)                 8.042281 0.19363186 74 41.53387  0.0000
## Fam1                       -0.315861 0.08340381 74 -3.78713  0.0003
## Fam2                       -0.320726 0.09091895 74 -3.52760  0.0007
## Mare                        0.015474 0.06843995 74  0.22610  0.8217
## Age                        -0.005310 0.00976081 74 -0.54398  0.5881
## snp                         0.196677 0.09585029 74  2.05191  0.0437
## Treat                      -0.065510 0.06041736 69 -1.08430  0.2820
## RNAseq_condition           -0.151596 0.09535555 74 -1.58979  0.1161
## snp:Treat                   0.010936 0.08804367 69  0.12421  0.9015
## snp:RNAseq_condition        0.038248 0.16428825 74  0.23281  0.8166
## Treat:RNAseq_condition      0.089765 0.08545310 69  1.05046  0.2972
## snp:Treat:RNAseq_condition -0.099570 0.15241413 69 -0.65329  0.5157
##  Correlation: 
##                            (Intr) Fam1   Fam2   Mare   Age    snp   
## Fam1                       -0.031                                   
## Fam2                       -0.379  0.208                            
## Mare                       -0.110 -0.004  0.048                     
## Age                        -0.902 -0.063  0.234 -0.125              
## snp                        -0.314  0.000  0.217  0.182  0.057       
## Treat                      -0.163 -0.005  0.003 -0.004  0.006  0.294
## RNAseq_condition           -0.433 -0.099  0.178  0.217  0.162  0.472
## snp:Treat                   0.137  0.005 -0.011 -0.004 -0.039 -0.547
## snp:RNAseq_condition        0.252  0.083 -0.120 -0.131 -0.111 -0.592
## Treat:RNAseq_condition      0.132 -0.027 -0.032 -0.004 -0.014 -0.214
## snp:Treat:RNAseq_condition -0.089  0.014  0.023  0.006  0.028  0.320
##                            Treat  RNAsq_ snp:Tr s:RNA_ T:RNA_
## Fam1                                                         
## Fam2                                                         
## Mare                                                         
## Age                                                          
## snp                                                          
## Treat                                                        
## RNAseq_condition            0.323                            
## snp:Treat                  -0.603 -0.211                     
## snp:RNAseq_condition       -0.172 -0.557  0.323              
## Treat:RNAseq_condition     -0.707 -0.486  0.427  0.271       
## snp:Treat:RNAseq_condition  0.348  0.266 -0.578 -0.506 -0.527
## 
## Standardized Within-Group Residuals:
##         Min          Q1         Med          Q3         Max 
## -2.48243194 -0.43032521 -0.07495933  0.57503975  1.79010647 
## 
## Number of Observations: 155
## Number of Groups: 82 
## [1] "Fixed Effect Regression Estimates of Mixed Model with REML"
```

```
## [1] "Added Variable aka. Partial Regression Plots: for multiple linear regression (no random effects)"
```

```
## 'data.frame':    155 obs. of  9 variables:
##  $ gene            : num  8.52 8.31 8.42 8.44 8.2 ...
##  $ snp             : num  0 0 0 0 0 0 0 0 0 0 ...
##  $ Fam1            : num  1 0 0 0 1 1 1 1 1 0 ...
##  $ Fam2            : num  0 1 1 1 0 0 0 0 0 1 ...
##  $ Mare            : num  1 1 1 0 0 0 0 1 1 0 ...
##  $ Age             : num  18 18 13 18 19 17 20 19 15 15 ...
##  $ RNAseq_condition: num  0 1 0 0 1 1 1 1 0 0 ...
##  $ Treat           : num  0 0 0 0 0 0 0 0 0 0 ...
##  $ ID              : chr  "26" "29" "30" "31" ...
## 'data.frame':    155 obs. of  9 variables:
##  $ gene            : num  8.52 8.31 8.42 8.44 8.2 ...
##  $ snp             : num  0 0 0 0 0 0 0 0 0 0 ...
##  $ Fam1            : num  1 0 0 0 1 1 1 1 1 0 ...
##  $ Fam2            : num  0 1 1 1 0 0 0 0 0 1 ...
##  $ Mare            : num  1 1 1 0 0 0 0 1 1 0 ...
##  $ Age             : num  18 18 13 18 19 17 20 19 15 15 ...
##  $ RNAseq_condition: num  0 1 0 0 1 1 1 1 0 0 ...
##  $ Treat           : num  0 0 0 0 0 0 0 0 0 0 ...
##  $ ID              : Factor w/ 82 levels "100","110","111",..: 5 6 7 8 10 12 13 14 16 17 ...
## [1] "chr21.52625145"
## [1] "DEXI"
## Linear mixed model fit by REML ['lmerMod']
## Formula: 
## gene ~ Fam1 + Fam2 + Mare + Age + snp * Treat * RNAseq_condition +  
##     (1 | ID)
##    Data: comb.df
## 
## REML criterion at convergence: -66.1
## 
## Scaled residuals: 
##      Min       1Q   Median       3Q      Max 
## -2.44197 -0.58652  0.06862  0.55319  2.10218 
## 
## Random effects:
##  Groups   Name        Variance Std.Dev.
##  ID       (Intercept) 0.006611 0.08131 
##  Residual             0.021997 0.14831 
## Number of obs: 155, groups:  ID, 82
## 
## Fixed effects:
##                              Estimate Std. Error t value
## (Intercept)                 8.1196487  0.0892400   90.99
## Fam1                        0.1957694  0.0400397    4.89
## Fam2                        0.1718561  0.0425541    4.04
## Mare                       -0.0124313  0.0317523   -0.39
## Age                        -0.0067252  0.0045303   -1.48
## snp                         0.0168574  0.0516094    0.33
## Treat                       0.0007581  0.0391939    0.02
## RNAseq_condition            0.0038373  0.0487853    0.08
## snp:Treat                   0.0316627  0.0601748    0.53
## snp:RNAseq_condition       -0.0407477  0.0811547   -0.50
## Treat:RNAseq_condition     -0.0157777  0.0583485   -0.27
## snp:Treat:RNAseq_condition  0.0057749  0.0974490    0.06
## 
## Correlation of Fixed Effects:
##             (Intr) Fam1   Fam2   Mare   Age    snp    Treat  RNAsq_ snp:Tr
## Fam1        -0.091                                                        
## Fam2        -0.370  0.246                                                 
## Mare        -0.027  0.012 -0.012                                          
## Age         -0.903 -0.031  0.240 -0.145                                   
## snp         -0.256  0.105  0.174 -0.101  0.051                            
## Treat       -0.227  0.010  0.017 -0.013 -0.006  0.359                     
## RNAsq_cndtn -0.338 -0.056  0.118  0.037  0.095  0.366  0.423              
## snp:Treat    0.158 -0.028 -0.024  0.034 -0.027 -0.681 -0.555 -0.245       
## snp:RNAsq_c  0.111  0.037 -0.050  0.132 -0.005 -0.626 -0.228 -0.554  0.431
## Trt:RNAsq_c  0.169 -0.045 -0.047  0.006 -0.005 -0.249 -0.672 -0.652  0.374
## snp:Tr:RNA_ -0.107  0.040  0.036 -0.019  0.022  0.425  0.343  0.372 -0.618
##             s:RNA_ T:RNA_
## Fam1                     
## Fam2                     
## Mare                     
## Age                      
## snp                      
## Treat                    
## RNAsq_cndtn              
## snp:Treat                
## snp:RNAsq_c              
## Trt:RNAsq_c  0.373       
## snp:Tr:RNA_ -0.651 -0.559
## Linear mixed-effects model fit by maximum likelihood
##  Data: comb.df 
##         AIC       BIC   logLik
##   -99.56226 -56.95431 63.78113
## 
## Random effects:
##  Formula: ~1 | ID
##         (Intercept)  Residual
## StdDev:  0.07303745 0.1445429
## 
## Fixed effects: gene ~ Fam1 + Fam2 + Mare + Age + snp * Treat * RNAseq_condition 
##                                Value  Std.Error DF  t-value p-value
## (Intercept)                 8.119561 0.08811285 74 92.14956  0.0000
## Fam1                        0.195407 0.03951581 74  4.94504  0.0000
## Fam2                        0.171803 0.04197794 74  4.09269  0.0001
## Mare                       -0.012652 0.03132644 74 -0.40387  0.6875
## Age                        -0.006693 0.00446688 74 -1.49845  0.1383
## snp                         0.016644 0.05151125 74  0.32312  0.7475
## Treat                       0.000462 0.03974447 69  0.01162  0.9908
## RNAseq_condition            0.003359 0.04866168 74  0.06902  0.9452
## snp:Treat                   0.031864 0.06095004 69  0.52278  0.6028
## snp:RNAseq_condition       -0.040408 0.08094953 74 -0.49918  0.6191
## Treat:RNAseq_condition     -0.015241 0.05914400 69 -0.25770  0.7974
## snp:Treat:RNAseq_condition  0.005334 0.09878523 69  0.05399  0.9571
##  Correlation: 
##                            (Intr) Fam1   Fam2   Mare   Age    snp   
## Fam1                       -0.091                                   
## Fam2                       -0.370  0.246                            
## Mare                       -0.026  0.013 -0.012                     
## Age                        -0.902 -0.030  0.239 -0.145              
## snp                        -0.259  0.105  0.173 -0.101  0.051       
## Treat                      -0.233  0.010  0.017 -0.013 -0.006  0.364
## RNAseq_condition           -0.340 -0.054  0.118  0.037  0.094  0.366
## snp:Treat                   0.162 -0.028 -0.024  0.034 -0.027 -0.689
## snp:RNAseq_condition        0.113  0.035 -0.051  0.131 -0.006 -0.627
## Treat:RNAseq_condition      0.173 -0.046 -0.048  0.006 -0.005 -0.253
## snp:Treat:RNAseq_condition -0.110  0.041  0.036 -0.020  0.022  0.430
##                            Treat  RNAsq_ snp:Tr s:RNA_ T:RNA_
## Fam1                                                         
## Fam2                                                         
## Mare                                                         
## Age                                                          
## snp                                                          
## Treat                                                        
## RNAseq_condition            0.429                            
## snp:Treat                  -0.555 -0.249                     
## snp:RNAseq_condition       -0.231 -0.555  0.437              
## Treat:RNAseq_condition     -0.673 -0.662  0.374  0.378       
## snp:Treat:RNAseq_condition  0.343  0.377 -0.618 -0.660 -0.559
## 
## Standardized Within-Group Residuals:
##         Min          Q1         Med          Q3         Max 
## -2.56741265 -0.60667338  0.06401093  0.58439093  2.23552109 
## 
## Number of Observations: 155
## Number of Groups: 82 
## [1] "Fixed Effect Regression Estimates of Mixed Model with REML"
```

```
## [1] "Added Variable aka. Partial Regression Plots: for multiple linear regression (no random effects)"
```

```
## 'data.frame':    155 obs. of  9 variables:
##  $ gene            : num  10.01 9.99 10.15 10 9.99 ...
##  $ snp             : num  0 0 0 0 0 0 0 0 0 0 ...
##  $ Fam1            : num  1 0 0 0 1 1 1 1 1 0 ...
##  $ Fam2            : num  0 1 1 1 0 0 0 0 0 1 ...
##  $ Mare            : num  1 1 1 0 0 0 0 1 1 0 ...
##  $ Age             : num  18 18 13 18 19 17 20 19 15 15 ...
##  $ RNAseq_condition: num  0 1 0 0 1 1 1 1 0 0 ...
##  $ Treat           : num  0 0 0 0 0 0 0 0 0 0 ...
##  $ ID              : chr  "26" "29" "30" "31" ...
## 'data.frame':    155 obs. of  9 variables:
##  $ gene            : num  10.01 9.99 10.15 10 9.99 ...
##  $ snp             : num  0 0 0 0 0 0 0 0 0 0 ...
##  $ Fam1            : num  1 0 0 0 1 1 1 1 1 0 ...
##  $ Fam2            : num  0 1 1 1 0 0 0 0 0 1 ...
##  $ Mare            : num  1 1 1 0 0 0 0 1 1 0 ...
##  $ Age             : num  18 18 13 18 19 17 20 19 15 15 ...
##  $ RNAseq_condition: num  0 1 0 0 1 1 1 1 0 0 ...
##  $ Treat           : num  0 0 0 0 0 0 0 0 0 0 ...
##  $ ID              : Factor w/ 82 levels "100","110","111",..: 5 6 7 8 10 12 13 14 16 17 ...
## [1] "chr21.52625145"
## [1] "NSUN2"
## Linear mixed model fit by REML ['lmerMod']
## Formula: 
## gene ~ Fam1 + Fam2 + Mare + Age + snp * Treat * RNAseq_condition +  
##     (1 | ID)
##    Data: comb.df
## 
## REML criterion at convergence: -224.6
## 
## Scaled residuals: 
##      Min       1Q   Median       3Q      Max 
## -1.85174 -0.54085  0.03495  0.53567  1.81030 
## 
## Random effects:
##  Groups   Name        Variance Std.Dev.
##  ID       (Intercept) 0.005791 0.07610 
##  Residual             0.005072 0.07122 
## Number of obs: 155, groups:  ID, 82
## 
## Fixed effects:
##                             Estimate Std. Error t value
## (Intercept)                10.091183   0.060339  167.24
## Fam1                       -0.022462   0.027135   -0.83
## Fam2                       -0.058909   0.028980   -2.03
## Mare                       -0.002502   0.021594   -0.12
## Age                        -0.002352   0.003100   -0.76
## snp                         0.059580   0.030980    1.92
## Treat                       0.171475   0.018939    9.05
## RNAseq_condition           -0.019365   0.029658   -0.65
## snp:Treat                  -0.021265   0.029431   -0.72
## snp:RNAseq_condition        0.033074   0.049352    0.67
## Treat:RNAseq_condition     -0.011836   0.028314   -0.42
## snp:Treat:RNAseq_condition -0.029502   0.047251   -0.62
## 
## Correlation of Fixed Effects:
##             (Intr) Fam1   Fam2   Mare   Age    snp    Treat  RNAsq_ snp:Tr
## Fam1        -0.086                                                        
## Fam2        -0.373  0.249                                                 
## Mare        -0.034  0.010 -0.013                                          
## Age         -0.916 -0.033  0.241 -0.138                                   
## snp         -0.232  0.105  0.185 -0.097  0.043                            
## Treat       -0.163  0.009  0.014 -0.011 -0.005  0.299                     
## RNAsq_cndtn -0.324 -0.087  0.107  0.046  0.105  0.364  0.339              
## snp:Treat    0.118 -0.023 -0.020  0.028 -0.023 -0.570 -0.554 -0.198       
## snp:RNAsq_c  0.081  0.062 -0.036  0.138  0.007 -0.614 -0.187 -0.546  0.356
## Trt:RNAsq_c  0.123 -0.039 -0.041  0.005 -0.004 -0.207 -0.669 -0.529  0.372
## snp:Tr:RNA_ -0.082  0.034  0.031 -0.017  0.019  0.359  0.345  0.304 -0.624
##             s:RNA_ T:RNA_
## Fam1                     
## Fam2                     
## Mare                     
## Age                      
## snp                      
## Treat                    
## RNAsq_cndtn              
## snp:Treat                
## snp:RNAsq_c              
## Trt:RNAsq_c  0.305       
## snp:Tr:RNA_ -0.527 -0.562
## Linear mixed-effects model fit by maximum likelihood
##  Data: comb.df 
##         AIC       BIC   logLik
##   -270.0545 -227.4465 149.0272
## 
## Random effects:
##  Formula: ~1 | ID
##         (Intercept)   Residual
## StdDev:  0.07107172 0.06944294
## 
## Fixed effects: gene ~ Fam1 + Fam2 + Mare + Age + snp * Treat * RNAseq_condition 
##                                Value  Std.Error DF   t-value p-value
## (Intercept)                10.091391 0.05953154 74 169.51335  0.0000
## Fam1                       -0.022515 0.02677134 74  -0.84100  0.4031
## Fam2                       -0.058929 0.02858169 74  -2.06176  0.0427
## Mare                       -0.002578 0.02129927 74  -0.12105  0.9040
## Age                        -0.002362 0.00305671 74  -0.77279  0.4421
## snp                         0.059251 0.03083146 74   1.92177  0.0585
## Treat                       0.171491 0.01921774 69   8.92355  0.0000
## RNAseq_condition           -0.019578 0.02947823 74  -0.66414  0.5087
## snp:Treat                  -0.020932 0.02983958 69  -0.70147  0.4854
## snp:RNAseq_condition        0.033587 0.04905069 74   0.68474  0.4956
## Treat:RNAseq_condition     -0.011632 0.02872311 69  -0.40498  0.6867
## snp:Treat:RNAseq_condition -0.030056 0.04793549 69  -0.62700  0.5327
##  Correlation: 
##                            (Intr) Fam1   Fam2   Mare   Age    snp   
## Fam1                       -0.087                                   
## Fam2                       -0.372  0.249                            
## Mare                       -0.034  0.010 -0.012                     
## Age                        -0.915 -0.033  0.241 -0.139              
## snp                        -0.234  0.105  0.184 -0.098  0.044       
## Treat                      -0.168  0.009  0.015 -0.011 -0.005  0.304
## RNAseq_condition           -0.325 -0.084  0.108  0.045  0.105  0.364
## snp:Treat                   0.121 -0.024 -0.020  0.029 -0.023 -0.580
## snp:RNAseq_condition        0.084  0.060 -0.037  0.137  0.006 -0.615
## Treat:RNAseq_condition      0.127 -0.039 -0.041  0.005 -0.004 -0.210
## snp:Treat:RNAseq_condition -0.084  0.035  0.031 -0.017  0.019  0.365
##                            Treat  RNAsq_ snp:Tr s:RNA_ T:RNA_
## Fam1                                                         
## Fam2                                                         
## Mare                                                         
## Age                                                          
## snp                                                          
## Treat                                                        
## RNAseq_condition            0.346                            
## snp:Treat                  -0.554 -0.202                     
## snp:RNAseq_condition       -0.190 -0.547  0.362              
## Treat:RNAseq_condition     -0.670 -0.539  0.372  0.311       
## snp:Treat:RNAseq_condition  0.345  0.310 -0.623 -0.538 -0.562
## 
## Standardized Within-Group Residuals:
##         Min          Q1         Med          Q3         Max 
## -1.94722580 -0.56697712  0.03336466  0.54877172  1.91086771 
## 
## Number of Observations: 155
## Number of Groups: 82 
## [1] "Fixed Effect Regression Estimates of Mixed Model with REML"
```

```
## [1] "Added Variable aka. Partial Regression Plots: for multiple linear regression (no random effects)"
```

```
## 'data.frame':    155 obs. of  9 variables:
##  $ gene            : num  7.27 6.57 6.05 6.37 6.02 ...
##  $ snp             : num  0 0 0 0 0 0 0 0 0 0 ...
##  $ Fam1            : num  1 0 0 0 1 1 1 1 1 0 ...
##  $ Fam2            : num  0 1 1 1 0 0 0 0 0 1 ...
##  $ Mare            : num  1 1 1 0 0 0 0 1 1 0 ...
##  $ Age             : num  18 18 13 18 19 17 20 19 15 15 ...
##  $ RNAseq_condition: num  0 1 0 0 1 1 1 1 0 0 ...
##  $ Treat           : num  0 0 0 0 0 0 0 0 0 0 ...
##  $ ID              : chr  "26" "29" "30" "31" ...
## 'data.frame':    155 obs. of  9 variables:
##  $ gene            : num  7.27 6.57 6.05 6.37 6.02 ...
##  $ snp             : num  0 0 0 0 0 0 0 0 0 0 ...
##  $ Fam1            : num  1 0 0 0 1 1 1 1 1 0 ...
##  $ Fam2            : num  0 1 1 1 0 0 0 0 0 1 ...
##  $ Mare            : num  1 1 1 0 0 0 0 1 1 0 ...
##  $ Age             : num  18 18 13 18 19 17 20 19 15 15 ...
##  $ RNAseq_condition: num  0 1 0 0 1 1 1 1 0 0 ...
##  $ Treat           : num  0 0 0 0 0 0 0 0 0 0 ...
##  $ ID              : Factor w/ 82 levels "100","110","111",..: 5 6 7 8 10 12 13 14 16 17 ...
## [1] "chr21.52625145"
## [1] "ATF7IP2"
## Linear mixed model fit by REML ['lmerMod']
## Formula: 
## gene ~ Fam1 + Fam2 + Mare + Age + snp * Treat * RNAseq_condition +  
##     (1 | ID)
##    Data: comb.df
## 
## REML criterion at convergence: 274
## 
## Scaled residuals: 
##     Min      1Q  Median      3Q     Max 
## -2.9112 -0.4767 -0.1188  0.4615  2.5339 
## 
## Random effects:
##  Groups   Name        Variance Std.Dev.
##  ID       (Intercept) 0.40015  0.6326  
##  Residual             0.09877  0.3143  
## Number of obs: 155, groups:  ID, 82
## 
## Fixed effects:
##                             Estimate Std. Error t value
## (Intercept)                 6.674443   0.438094  15.235
## Fam1                       -0.242308   0.196882  -1.231
## Fam2                        0.118985   0.211134   0.564
## Mare                       -0.003126   0.157142  -0.020
## Age                        -0.007672   0.022685  -0.338
## snp                        -0.336745   0.200732  -1.678
## Treat                       0.316482   0.084038   3.766
## RNAseq_condition            0.058165   0.196448   0.296
## snp:Treat                   0.158329   0.131930   1.200
## snp:RNAseq_condition        0.330586   0.327186   1.010
## Treat:RNAseq_condition     -0.040296   0.126076  -0.320
## snp:Treat:RNAseq_condition  0.087247   0.210260   0.415
## 
## Correlation of Fixed Effects:
##             (Intr) Fam1   Fam2   Mare   Age    snp    Treat  RNAsq_ snp:Tr
## Fam1        -0.082                                                        
## Fam2        -0.373  0.252                                                 
## Mare        -0.040  0.008 -0.013                                          
## Age         -0.925 -0.036  0.243 -0.133                                   
## snp         -0.212  0.104  0.196 -0.092  0.034                            
## Treat       -0.100  0.006  0.010 -0.008 -0.003  0.210                     
## RNAsq_cndtn -0.315 -0.118  0.093  0.054  0.115  0.363  0.229              
## snp:Treat    0.075 -0.016 -0.014  0.020 -0.016 -0.403 -0.554 -0.134       
## snp:RNAsq_c  0.054  0.088 -0.021  0.141  0.020 -0.595 -0.128 -0.535  0.245
## Trt:RNAsq_c  0.077 -0.027 -0.028  0.004 -0.003 -0.145 -0.667 -0.361  0.370
## snp:Tr:RNA_ -0.053  0.024  0.022 -0.011  0.013  0.256  0.348  0.209 -0.628
##             s:RNA_ T:RNA_
## Fam1                     
## Fam2                     
## Mare                     
## Age                      
## snp                      
## Treat                    
## RNAsq_cndtn              
## snp:Treat                
## snp:RNAsq_c              
## Trt:RNAsq_c  0.209       
## snp:Tr:RNA_ -0.359 -0.565
## Linear mixed-effects model fit by maximum likelihood
##  Data: comb.df 
##        AIC      BIC    logLik
##   272.3249 314.9328 -122.1624
## 
## Random effects:
##  Formula: ~1 | ID
##         (Intercept)  Residual
## StdDev:   0.5989225 0.3055785
## 
## Fixed effects: gene ~ Fam1 + Fam2 + Mare + Age + snp * Treat * RNAseq_condition 
##                                Value Std.Error DF   t-value p-value
## (Intercept)                 6.674456 0.4332852 74 15.404304  0.0000
## Fam1                       -0.242245 0.1947302 74 -1.244002  0.2174
## Fam2                        0.118870 0.2087998 74  0.569299  0.5709
## Mare                       -0.003157 0.1554099 74 -0.020313  0.9838
## Age                        -0.007663 0.0224314 74 -0.341615  0.7336
## snp                        -0.335803 0.1992905 74 -1.684993  0.0962
## Treat                       0.316339 0.0850551 69  3.719223  0.0004
## RNAseq_condition            0.057930 0.1948621 74  0.297290  0.7671
## snp:Treat                   0.157371 0.1334846 69  1.178948  0.2425
## snp:RNAseq_condition        0.329725 0.3245329 74  1.015998  0.3129
## Treat:RNAseq_condition     -0.040077 0.1275883 69 -0.314112  0.7544
## snp:Treat:RNAseq_condition  0.088128 0.2127863 69  0.414163  0.6800
##  Correlation: 
##                            (Intr) Fam1   Fam2   Mare   Age    snp   
## Fam1                       -0.082                                   
## Fam2                       -0.373  0.251                            
## Mare                       -0.040  0.008 -0.013                     
## Age                        -0.925 -0.036  0.243 -0.133              
## snp                        -0.213  0.104  0.195 -0.093  0.034       
## Treat                      -0.103  0.006  0.010 -0.008 -0.004  0.214
## RNAseq_condition           -0.315 -0.117  0.093  0.054  0.114  0.363
## snp:Treat                   0.076 -0.016 -0.014  0.020 -0.016 -0.411
## snp:RNAseq_condition        0.054  0.087 -0.022  0.141  0.019 -0.596
## Treat:RNAseq_condition      0.078 -0.027 -0.029  0.004 -0.003 -0.148
## snp:Treat:RNAseq_condition -0.054  0.024  0.022 -0.012  0.013  0.261
##                            Treat  RNAsq_ snp:Tr s:RNA_ T:RNA_
## Fam1                                                         
## Fam2                                                         
## Mare                                                         
## Age                                                          
## snp                                                          
## Treat                                                        
## RNAseq_condition            0.234                            
## snp:Treat                  -0.554 -0.137                     
## snp:RNAseq_condition       -0.131 -0.535  0.250              
## Treat:RNAseq_condition     -0.667 -0.368  0.370  0.213       
## snp:Treat:RNAseq_condition  0.348  0.213 -0.628 -0.366 -0.565
## 
## Standardized Within-Group Residuals:
##        Min         Q1        Med         Q3        Max 
## -3.0037405 -0.4888443 -0.1226847  0.4770809  2.5968984 
## 
## Number of Observations: 155
## Number of Groups: 82 
## [1] "Fixed Effect Regression Estimates of Mixed Model with REML"
```

```
## [1] "Added Variable aka. Partial Regression Plots: for multiple linear regression (no random effects)"
```

```
## 'data.frame':    155 obs. of  9 variables:
##  $ gene            : num  7.63 7.92 7.58 7.29 8 ...
##  $ snp             : num  0 0 0 0 0 0 0 0 0 0 ...
##  $ Fam1            : num  1 0 0 0 1 1 1 1 1 0 ...
##  $ Fam2            : num  0 1 1 1 0 0 0 0 0 1 ...
##  $ Mare            : num  1 1 1 0 0 0 0 1 1 0 ...
##  $ Age             : num  18 18 13 18 19 17 20 19 15 15 ...
##  $ RNAseq_condition: num  0 1 0 0 1 1 1 1 0 0 ...
##  $ Treat           : num  0 0 0 0 0 0 0 0 0 0 ...
##  $ ID              : chr  "26" "29" "30" "31" ...
## 'data.frame':    155 obs. of  9 variables:
##  $ gene            : num  7.63 7.92 7.58 7.29 8 ...
##  $ snp             : num  0 0 0 0 0 0 0 0 0 0 ...
##  $ Fam1            : num  1 0 0 0 1 1 1 1 1 0 ...
##  $ Fam2            : num  0 1 1 1 0 0 0 0 0 1 ...
##  $ Mare            : num  1 1 1 0 0 0 0 1 1 0 ...
##  $ Age             : num  18 18 13 18 19 17 20 19 15 15 ...
##  $ RNAseq_condition: num  0 1 0 0 1 1 1 1 0 0 ...
##  $ Treat           : num  0 0 0 0 0 0 0 0 0 0 ...
##  $ ID              : Factor w/ 82 levels "100","110","111",..: 5 6 7 8 10 12 13 14 16 17 ...
## [1] "chr21.52625145"
## [1] "GLIPR1L2"
## Linear mixed model fit by REML ['lmerMod']
## Formula: 
## gene ~ Fam1 + Fam2 + Mare + Age + snp * Treat * RNAseq_condition +  
##     (1 | ID)
##    Data: comb.df
## 
## REML criterion at convergence: 103.2
## 
## Scaled residuals: 
##      Min       1Q   Median       3Q      Max 
## -2.14268 -0.44864 -0.01285  0.52810  1.90413 
## 
## Random effects:
##  Groups   Name        Variance Std.Dev.
##  ID       (Intercept) 0.06871  0.2621  
##  Residual             0.04525  0.2127  
## Number of obs: 155, groups:  ID, 82
## 
## Fixed effects:
##                             Estimate Std. Error t value
## (Intercept)                 8.079838   0.199177   40.57
## Fam1                       -0.310828   0.089574   -3.47
## Fam2                       -0.345198   0.095768   -3.60
## Mare                       -0.025695   0.071338   -0.36
## Age                        -0.003637   0.010257   -0.35
## snp                         0.127431   0.099390    1.28
## Treat                      -0.053244   0.056650   -0.94
## RNAseq_condition           -0.130351   0.095589   -1.36
## snp:Treat                  -0.012991   0.088272   -0.15
## snp:RNAseq_condition       -0.170706   0.159092   -1.07
## Treat:RNAseq_condition      0.003342   0.084773    0.04
## snp:Treat:RNAseq_condition  0.172689   0.141444    1.22
## 
## Correlation of Fixed Effects:
##             (Intr) Fam1   Fam2   Mare   Age    snp    Treat  RNAsq_ snp:Tr
## Fam1        -0.085                                                        
## Fam2        -0.373  0.250                                                 
## Mare        -0.036  0.009 -0.013                                          
## Age         -0.919 -0.034  0.242 -0.137                                   
## snp         -0.227  0.105  0.188 -0.096  0.041                            
## Treat       -0.148  0.008  0.014 -0.010 -0.005  0.280                     
## RNAsq_cndtn -0.321 -0.094  0.104  0.048  0.108  0.364  0.316              
## snp:Treat    0.108 -0.022 -0.019  0.027 -0.021 -0.536 -0.554 -0.184       
## snp:RNAsq_c  0.074  0.068 -0.033  0.139  0.010 -0.610 -0.175 -0.544  0.333
## Trt:RNAsq_c  0.112 -0.036 -0.038  0.005 -0.004 -0.194 -0.669 -0.493  0.371
## snp:Tr:RNA_ -0.075  0.032  0.029 -0.016  0.017  0.339  0.346  0.284 -0.625
##             s:RNA_ T:RNA_
## Fam1                     
## Fam2                     
## Mare                     
## Age                      
## snp                      
## Treat                    
## RNAsq_cndtn              
## snp:Treat                
## snp:RNAsq_c              
## Trt:RNAsq_c  0.285       
## snp:Tr:RNA_ -0.491 -0.563
## Linear mixed-effects model fit by maximum likelihood
##  Data: comb.df 
##        AIC      BIC    logLik
##   85.70916 128.3171 -28.85458
## 
## Random effects:
##  Formula: ~1 | ID
##         (Intercept)  Residual
## StdDev:   0.2466719 0.2069186
## 
## Fixed effects: gene ~ Fam1 + Fam2 + Mare + Age + snp * Treat * RNAseq_condition 
##                                Value  Std.Error DF  t-value p-value
## (Intercept)                 8.080658 0.19693093 74 41.03295  0.0000
## Fam1                       -0.310736 0.08856480 74 -3.50858  0.0008
## Fam2                       -0.345366 0.09466529 74 -3.64829  0.0005
## Mare                       -0.025562 0.07052179 74 -0.36247  0.7180
## Age                        -0.003682 0.01013641 74 -0.36325  0.7175
## snp                         0.125868 0.09891702 74  1.27246  0.2072
## Treat                      -0.053301 0.05735194 69 -0.92936  0.3559
## RNAseq_condition           -0.130512 0.09502807 74 -1.37340  0.1738
## snp:Treat                  -0.011455 0.08931114 69 -0.12826  0.8983
## snp:RNAseq_condition       -0.169090 0.15815114 74 -1.06917  0.2885
## Treat:RNAseq_condition      0.003449 0.08580523 69  0.04020  0.9680
## snp:Treat:RNAseq_condition  0.171103 0.14317206 69  1.19508  0.2361
##  Correlation: 
##                            (Intr) Fam1   Fam2   Mare   Age    snp   
## Fam1                       -0.086                                   
## Fam2                       -0.373  0.250                            
## Mare                       -0.035  0.009 -0.013                     
## Age                        -0.918 -0.034  0.242 -0.137              
## snp                        -0.228  0.105  0.187 -0.097  0.042       
## Treat                      -0.152  0.008  0.014 -0.010 -0.005  0.285
## RNAseq_condition           -0.322 -0.093  0.104  0.047  0.107  0.364
## snp:Treat                   0.110 -0.022 -0.019  0.027 -0.022 -0.544
## snp:RNAseq_condition        0.076  0.067 -0.034  0.138  0.009 -0.611
## Treat:RNAseq_condition      0.115 -0.037 -0.039  0.005 -0.004 -0.197
## snp:Treat:RNAseq_condition -0.077  0.033  0.030 -0.016  0.018  0.344
##                            Treat  RNAsq_ snp:Tr s:RNA_ T:RNA_
## Fam1                                                         
## Fam2                                                         
## Mare                                                         
## Age                                                          
## snp                                                          
## Treat                                                        
## RNAseq_condition            0.321                            
## snp:Treat                  -0.554 -0.187                     
## snp:RNAseq_condition       -0.178 -0.544  0.338              
## Treat:RNAseq_condition     -0.669 -0.502  0.371  0.290       
## snp:Treat:RNAseq_condition  0.346  0.289 -0.624 -0.500 -0.563
## 
## Standardized Within-Group Residuals:
##         Min          Q1         Med          Q3         Max 
## -2.23927621 -0.46973690 -0.01744856  0.55784032  1.97951407 
## 
## Number of Observations: 155
## Number of Groups: 82 
## [1] "Fixed Effect Regression Estimates of Mixed Model with REML"
```

```
## [1] "Added Variable aka. Partial Regression Plots: for multiple linear regression (no random effects)"
```
