# Supplementary material for: eQTL discovery and their association with severe equine asthma in European Warmblood horses
Source: BMC Genomics. 2018 Aug 2;19:581. doi: 10.1186/s12864-018-4938-9 (PMC6090848; doi:10.1186/s12864-018-4938-9)
Supplement: Supplementary file 3 — Association of DEXI and NSUN2 gene expression to RAO disease status. Html output of an R markdown document. The file contains two multiple logistic regressions and one simple logistic regression showing the association between DEXI gene expression and disease status. Multiple logistic regression with known confounders as independent variables, and the simple logistic regression only has the independent variable of interest (DEXI or NSUN2 gene expression) as the single covariate. An R markdown document that generated this html file is available on GitHub: https://github.com/VCMason. (HTML 844 kb) [file 12864_2018_4938_MOESM3_ESM.html]

DEXI and NSUN2 Gene Expression Associated To DiseaseStatus


# DEXI and NSUN2 Gene Expression Associated To DiseaseStatus

#### *Victor Mason*

#### *11 Januar 2018*

## Read in data

```
mck.genes <- read.table(file="D:\\LinuxShare\\Programs\\MatrixEQTL\\FormatDataIN\\MCK1_no559443_1056195_Fix111\\DESeq.NCBI.MCK1.m20.wVSD.AgeAsNumericFix111.tx.tab", sep="\t", header=TRUE)
mck.covs <- read.table(file="D:\\LinuxShare\\Programs\\MatrixEQTL\\FormatDataIN\\MCK1_no559443_1056195_Fix111\\RAO_database.VCM.v4.MCK1.no559443.MatrixeQTL.ForR.csv", sep=",", header=TRUE)

mck.genes.DEXI <- as.data.frame(mck.genes["DEXI",])
mck.genes.NSUN2 <- as.data.frame(mck.genes["NSUN2",])

df <- rbind(mck.genes.DEXI, mck.covs)
dim(df)
```

```
## [1]  6 73
```

```
t.df <- as.data.frame(t(df))
dim(t.df)
```

```
## [1] 73  6
```

```
df2 <- rbind(mck.genes.NSUN2, mck.covs)
dim(df2)
```

```
## [1]  6 73
```

```
t.df2 <- as.data.frame(t(df2))
dim(t.df2)
```

```
## [1] 73  6
```

## Fit logistic regression without surrogate variables

### No significant surrogate variables were estimated by SVA

### Model:

DiseaseStatus ~ GeneExpression + Fam1 + Fam2 + Mare + Age

DiseaseStatus = RNAseq\_condition = 0 (healthy), or 1 (RAO horse)  
GeneExpression = DESeq2 normalized and variance stabilized gene expression values for genes not removed by the KS test for both treatments MCK and HDE  
Fam1 = 0 (unrelated horses) or 1 (Family 1 horses)  
Fam2 = 0 (unrelated horses) or 1 (Family 2 horses)  
Mare = 0 (male (stallion or gelding)) or1 (Mare)  
Age = year of sample collection - year of birth (a quantitative variable)  
Genotype = snp = 0,1,2  
Treatment = Treat = 0 (MCK), or 1 (HDE) No significant surrogate variables estimated by SVA

## DEXI

```
mod2 <- glm(RNAseq_condition ~ ., data=t.df, family=binomial(link="logit"))
summary(mod2)
```

```
## 
## Call:
## glm(formula = RNAseq_condition ~ ., family = binomial(link = "logit"), 
##     data = t.df)
## 
## Deviance Residuals: 
##     Min       1Q   Median       3Q      Max  
## -1.6689  -1.0237  -0.7518   1.0721   1.8521  
## 
## Coefficients:
##             Estimate Std. Error z value Pr(>|z|)
## (Intercept)  2.71247   12.81953   0.212    0.832
## DEXI        -0.13653    1.58031  -0.086    0.931
## Fam1         0.32649    0.78550   0.416    0.678
## Fam2        -0.84657    0.82313  -1.028    0.304
## Mare        -0.76109    0.50897  -1.495    0.135
## Age         -0.08263    0.07786  -1.061    0.289
## 
## (Dispersion parameter for binomial family taken to be 1)
## 
##     Null deviance: 100.527  on 72  degrees of freedom
## Residual deviance:  94.696  on 67  degrees of freedom
## AIC: 106.7
## 
## Number of Fisher Scoring iterations: 4
```

```
library(car)
avPlots(mod2)
```

## Fit simple logistic regression without known covariates or surrogate variables

### Model:

DiseaseStatus ~ GeneExpression ##DEXI

```
mod3 <- glm(RNAseq_condition ~ DEXI, data=t.df, family=binomial(link="logit"))
summary(mod3)
```

```
## 
## Call:
## glm(formula = RNAseq_condition ~ DEXI, family = binomial(link = "logit"), 
##     data = t.df)
## 
## Deviance Residuals: 
##    Min      1Q  Median      3Q     Max  
## -1.147  -1.103  -1.066   1.258   1.295  
## 
## Coefficients:
##             Estimate Std. Error z value Pr(>|z|)
## (Intercept)   1.6905     9.0706   0.186    0.852
## DEXI         -0.2334     1.1243  -0.208    0.836
## 
## (Dispersion parameter for binomial family taken to be 1)
## 
##     Null deviance: 100.53  on 72  degrees of freedom
## Residual deviance: 100.48  on 71  degrees of freedom
## AIC: 104.48
## 
## Number of Fisher Scoring iterations: 3
```

```
library(car)
avPlots(mod3)
```

## Fit logistic regression without surrogate variables

### No significant surrogate variables were estimated by SVA

### Model:

DiseaseStatus ~ GeneExpression + Fam1 + Fam2 + Mare + Age

## NSUN2

```
mod2 <- glm(RNAseq_condition ~ ., data=t.df2, family=binomial(link="logit"))
summary(mod2)
```

```
## 
## Call:
## glm(formula = RNAseq_condition ~ ., family = binomial(link = "logit"), 
##     data = t.df2)
## 
## Deviance Residuals: 
##     Min       1Q   Median       3Q      Max  
## -1.6499  -1.0283  -0.7105   1.0804   1.8465  
## 
## Coefficients:
##             Estimate Std. Error z value Pr(>|z|)
## (Intercept) 10.52294   26.90599   0.391    0.696
## NSUN2       -0.88623    2.67117  -0.332    0.740
## Fam1         0.23830    0.66086   0.361    0.718
## Fam2        -0.94837    0.72278  -1.312    0.189
## Mare        -0.76913    0.51050  -1.507    0.132
## Age         -0.08078    0.07718  -1.047    0.295
## 
## (Dispersion parameter for binomial family taken to be 1)
## 
##     Null deviance: 100.527  on 72  degrees of freedom
## Residual deviance:  94.593  on 67  degrees of freedom
## AIC: 106.59
## 
## Number of Fisher Scoring iterations: 4
```

```
library(car)
avPlots(mod2)
```

## Fit simple logistic regression without known covariates or surrogate variables

### Model:

DiseaseStatus ~ GeneExpression ##NSUN2

```
mod3 <- glm(RNAseq_condition ~ NSUN2, data=t.df2, family=binomial(link="logit"))
summary(mod3)
```

```
## 
## Call:
## glm(formula = RNAseq_condition ~ NSUN2, family = binomial(link = "logit"), 
##     data = t.df2)
## 
## Deviance Residuals: 
##    Min      1Q  Median      3Q     Max  
## -1.121  -1.097  -1.085   1.260   1.282  
## 
## Coefficients:
##             Estimate Std. Error z value Pr(>|z|)
## (Intercept)   2.2382    24.1116   0.093    0.926
## NSUN2        -0.2419     2.3995  -0.101    0.920
## 
## (Dispersion parameter for binomial family taken to be 1)
## 
##     Null deviance: 100.53  on 72  degrees of freedom
## Residual deviance: 100.52  on 71  degrees of freedom
## AIC: 104.52
## 
## Number of Fisher Scoring iterations: 3
```

```
library(car)
avPlots(mod3)
```
