# Supplementary material for: eQTL discovery and their association with severe equine asthma in European Warmblood horses
Source: BMC Genomics. 2018 Aug 2;19:581. doi: 10.1186/s12864-018-4938-9 (PMC6090848; doi:10.1186/s12864-018-4938-9)
Supplement: Supplementary file 4 — Association of ATF7IP2, and GLIPR1L2 gene expression to RAO disease status. Html output of an R markdown document. The file contains one multiple logistic regression for each gene ATF7IP2, and GLIPR1L2. These models quantify the association between gene expression in ATF7IP2, or GLIPR1L2 and disease status. Four significant surrogate variables were calculated for the HDE treatment by SVA and therefore none were included in the model. An R markdown document that generated this html file is available on GitHub: https://github.com/VCMason. (HTML 866 kb) [file 12864_2018_4938_MOESM4_ESM.html]

ATF7IP2, and GLIPR1L2 Gene Expression Associated To Disease Status


# ATF7IP2, and GLIPR1L2 Gene Expression Associated To Disease Status

#### *Victor Mason*

#### *11 Januar 2018*

## Read in data

```
all.genes <- read.table(file="D:\\LinuxShare\\Programs\\MatrixEQTL\\FormatDataIN\\HDE9_no559443_1056195_Fix111\\DESeq.NCBI.HDE9.m24.wVSD.AgeAsNumericFix111.tx.tab", sep="\t", header=TRUE, check.names = FALSE)
all.covs <- read.table(file="D:\\LinuxShare\\Programs\\MatrixEQTL\\FormatDataIN\\HDE9_no559443_1056195_Fix111\\RAO_database.VCM.v4.HDE9.no559443.MatrixeQTL.w4SVLeek.ForR.csv", sep=",", header=TRUE, check.names = FALSE)
```

## Construct dataframe for analysis

```
MakeDF <- function(genename, common.covs, all.covs)
{

g <- as.data.frame(all.genes[genename,])

c <- all.covs[,match(colnames(g), colnames(all.covs))] #match individuals with expression data in HDE individuals with covariate data.
c <- c[rownames(c) %in% common.covs,]

df <- rbind(g, c)
dim(df)
t.df <- as.data.frame(t(df))
dim(t.df)

return(t.df)

}
```

## Fit multiple logistic regression

### Plot added variable / Partial regression plots

### Model:

DiseaseStatus ~ GeneExpression

DiseaseStatus = RNAseq\_condition = 0 (healthy), or 1 (RAO horse) GeneExpression = normalized and variance stabilized gene expression value for genes not removed by the KS test for both treatments MCK and HDE HDE gene expression only

```
FitModel <- function(t.df, gene)
{

mod <- glm(RNAseq_condition ~ ., data=t.df, family=binomial(link="logit"))
print(summary(mod))

library(coefplot2)
coefplot2(mod)

library(car)
avPlots(mod)

}
```

## Make DFs for each gene and covariates

## Fit Model for each gene

```
genes <- c("ATF7IP2", "GLIPR1L2")  #"DEXI", 
common.covs <- c("Fam1", "Fam2", "Mare", "Age", "SV1", "SV2", "SV3", "SV4", "RNAseq_condition")

for ( gene in genes )
{

print(gene)
comb.df <- MakeDF(gene, common.covs, all.covs)

FitModel(comb.df, gene)

}
```

```
## [1] "ATF7IP2"
## 
## Call:
## glm(formula = RNAseq_condition ~ ., family = binomial(link = "logit"), 
##     data = t.df)
## 
## Deviance Residuals: 
##     Min       1Q   Median       3Q      Max  
## -1.5116  -0.9843  -0.5085   1.0404   2.0713  
## 
## Coefficients:
##             Estimate Std. Error z value Pr(>|z|)  
## (Intercept)  0.25608    3.71021   0.069   0.9450  
## ATF7IP2      0.28885    0.46534   0.621   0.5348  
## Fam1        -0.26130    0.90530  -0.289   0.7729  
## Fam2        -1.36153    0.78713  -1.730   0.0837 .
## Mare        -0.72114    0.50015  -1.442   0.1493  
## Age         -0.10393    0.07993  -1.300   0.1935  
## SV1         -0.84887    2.72263  -0.312   0.7552  
## SV2         -0.95020    2.60089  -0.365   0.7149  
## SV3          6.05791    3.18345   1.903   0.0570 .
## SV4         -4.22751    2.77945  -1.521   0.1283  
## ---
## Signif. codes:  0 '***' 0.001 '**' 0.01 '*' 0.05 '.' 0.1 ' ' 1
## 
## (Dispersion parameter for binomial family taken to be 1)
## 
##     Null deviance: 113.237  on 81  degrees of freedom
## Residual deviance:  99.171  on 72  degrees of freedom
## AIC: 119.17
## 
## Number of Fisher Scoring iterations: 4
```

```
## Loading required package: coda
```

```
## [1] "GLIPR1L2"
## 
## Call:
## glm(formula = RNAseq_condition ~ ., family = binomial(link = "logit"), 
##     data = t.df)
## 
## Deviance Residuals: 
##     Min       1Q   Median       3Q      Max  
## -1.5837  -1.0246  -0.4898   1.0755   2.0655  
## 
## Coefficients:
##             Estimate Std. Error z value Pr(>|z|)  
## (Intercept) 10.93610   11.71426   0.934   0.3505  
## GLIPR1L2    -1.08086    1.46945  -0.736   0.4620  
## Fam1        -0.51935    0.90377  -0.575   0.5655  
## Fam2        -1.48866    0.83391  -1.785   0.0742 .
## Mare        -0.71356    0.49930  -1.429   0.1530  
## Age         -0.11478    0.07814  -1.469   0.1419  
## SV1         -1.04578    2.57426  -0.406   0.6846  
## SV2          0.14635    2.36564   0.062   0.9507  
## SV3          4.26634    4.20487   1.015   0.3103  
## SV4         -4.22920    2.77200  -1.526   0.1271  
## ---
## Signif. codes:  0 '***' 0.001 '**' 0.01 '*' 0.05 '.' 0.1 ' ' 1
## 
## (Dispersion parameter for binomial family taken to be 1)
## 
##     Null deviance: 113.237  on 81  degrees of freedom
## Residual deviance:  99.017  on 72  degrees of freedom
## AIC: 119.02
## 
## Number of Fisher Scoring iterations: 4
```
